# iTRAQ-based quantitative proteome and phosphoprotein characterization reveals the central metabolism changes involved in wheat grain development

**DOI:** 10.1186/1471-2164-15-1029

**Published:** 2014-11-27

**Authors:** Chaoying Ma, Jianwen Zhou, Guanxing Chen, Yanwei Bian, Dongwen Lv, Xiaohui Li, Zhimin Wang, Yueming Yan

**Affiliations:** College of Life Sciences, Capital Normal University, Beijing, 100048 China; College of Agriculture and Biotechnology, China Agricultural University, Beijing, 100094 China

**Keywords:** Wheat, Grain proteome, iTRAQ, Phosphoproteins, qRT-PCR

## Abstract

**Background:**

Wheat (*Triticum aestivum* L.) is an economically important grain crop. Two-dimensional gel-based approaches are limited by the low identification rate of proteins and lack of accurate protein quantitation. The recently developed isobaric tag for relative and absolute quantitation (iTRAQ) method allows sensitive and accurate protein quantification. Here, we performed the first iTRAQ-based quantitative proteome and phosphorylated proteins analyses during wheat grain development.

**Results:**

The proteome profiles and phosphoprotein characterization of the metabolic proteins during grain development of the elite Chinese bread wheat cultivar Yanyou 361 were studied using the iTRAQ-based quantitative proteome approach, TiO_2_ microcolumns, and liquid chromatography-tandem mass spectrometry (LC-MS/MS). Among 1,146 non-redundant proteins identified, 421 showed at least 2-fold differences in abundance, and they were identified as differentially expressed proteins (DEPs), including 256 upregulated and 165 downregulated proteins. Of the 421 DEPs, six protein expression patterns were identified, most of which were up, down, and up-down expression patterns. The 421 DEPs were classified into nine functional categories mainly involved in different metabolic processes and located in the membrane and cytoplasm. Hierarchical clustering analysis indicated that the DEPs involved in starch biosynthesis, storage proteins, and defense/stress-related proteins significantly accumulated at the late grain development stages, while those related to protein synthesis/assembly/degradation and photosynthesis showed an opposite expression model during grain development. Quantitative real-time polymerase chain reaction (qRT-PCR) analysis of 12 representative genes encoding different metabolic proteins showed certain transcriptional and translational expression differences during grain development. Phosphorylated proteins analyses demonstrated that 23 DEPs such as AGPase, sucrose synthase, Hsp90, and serpins were phosphorylated in the developing grains and were mainly involved in starch biosynthesis and stress/defense.

**Conclusions:**

Our results revealed a complex quantitative proteome and phosphorylation profile during wheat grain development. Numerous DEPs are involved in grain starch and protein syntheses as well as adverse defense, which set an important basis for wheat yield and quality. Particularly, some key DEPs involved in starch biosynthesis and stress/defense were phosphorylated, suggesting their roles in wheat grain development.

**Electronic supplementary material:**

The online version of this article (doi:10.1186/1471-2164-15-1029) contains supplementary material, which is available to authorized users.

## Background

Wheat (*Triticum aestivum* L., 2n =6× =42, AABBDD) is one of the most important grain crops in the world. With a global output of 681 million tons in 2011, bread wheat accounts for 20% of the calories consumed by humans [1]. It is well known that wheat grain proteins are classified into two major categories: non-prolamins, including water-soluble albumins, and salt-soluble globulins and prolamins, including gliadins and glutenins. Gliadins and glutenins are the major storage proteins in wheat endosperm, are closely related to the viscoelastic properties of dough, and influence the processing and rheological properties of wheat flour [2]. Albumins and globulins, which include various enzymes and their inhibitors that play important roles in plant growth and development, are soluble proteins [3]. These proteins are relatively well balanced for human nutrition because of their high level of essential amino acids such as lysine, tryptophan, and methionine [4].

The development of the caryopsis can be broadly divided into three main phases: division and expansion, grain filling and maturation, and desiccation [5]. Proteins and carbohydrates that accumulate during seed development are essential reserves to support germination and early seedling growth as well as a major source of food for humans and animals. Because the quality of wheat is largely determined by events occurring during grain development, understanding protein synthesis and regulation during grain development is of fundamental importance for the improvement of wheat quality. Starch and storage proteins are two major components stored in wheat grains. They show similar patterns of accumulation and their deposition is observed during the grain-filling period between about 14 and 28 days post-anthesis (DPA) in the Hereward cultivar [5]. Based on the diameter, mature wheat endosperm contains A-type (diameter greater than 10 *μ*m), B-type (5–9 *μ*m in diameter), and C-type (diameter less than 5 *μ*m) starch granules. However, the C-type granules are so small that they are hardly isolated from the B-type, which commonly leads to them being classified as B-type granules [6]. The A-type granules, which continue to increase in size from the early stage in grain development until maturation, constitute approximately 70% of the volume and 10% of the total number of granules [7], while B and C-type granules that increase in size during the middle and late stages, respectively, account for about 30% of the volume and 90% of the total number of granules [6, 7].

Transcriptomic and proteomic approaches have provided insights into the mechanism of reserve accumulation during wheat grain development [8, 9]. However, poor correlation was observed between the accumulation of a large proportion of proteins and their corresponding mRNAs expression profiles in wheat [10] as well as among other plant species such as *Arabidopsis*[11] and rice [12]. Hence, proteome approaches provide a more valuable tool for monitoring developmental profiles and have been widely used in studies of maize [13] and rice [14]. Extensive research has been conducted to investigate protein synthesis and accumulation, and a number of proteomic studies have focused on identifying the array of proteins in developing wheat grains. Recent studies of different grain developing stages [10, 15–17] conducted using proteomic approaches have provided valuable information for the interpretation of the biochemical processes of wheat grain development, and most of them have been performed by two-dimensional electrophoresis (2-DE) [5, 10] and two-dimensional differential gel electrophoresis (2D-DIGE) [10, 18]. However, 2D-gel–based approaches have several limitations such as low identification rate of proteins, lack of accurate quantification of diffident proteins, low reproducibility and difficult separation of hydrophobic proteins [19, 20]. As a powerful technique to perform quantitative proteome analysis, isobaric tag for relative and absolute quantitation (iTRAQ) allows identification of more numerous proteins and can provide more reliable quantitative information than traditional 2-DE analysis [21]. In addition, with a sufficient number of proteins it is possible to conduct pathway and protein-protein interaction analyses [22]. The first shotgun proteomics study in wheat by iTRAQ was performed for investigating the protein responses to drought [23]. Recently, a metabolic pathway of wheat seedling growth under hydrogen peroxide stress was revealed by iTRAQ-based quantitative proteomic analysis [22]. To date, however, quantitative proteomics studies on wheat grain development based on iTRAQ analysis have not been reported.

It is well known that protein phosphorylation is one of the most common and important post-translational modifications (PTMs). A large number of biological processes are regulated by protein phosphorylation, including metabolism, transcription and translation, protein degradation, homeostasis, and cellular signaling and communication [24]. Recently, phosphoproteomic analysis has been carried out to elucidate plant growth, development, and diverse response mechanisms in different plants such as *Arabidopsis thaliana*[25], maize [26], rice [27], *Brachypodium distachyon*[28], and wheat [29]. These investigations showed that many stress-related proteins were phosphorylated under diverse conditions, suggesting the important role of protein phosphorylation in response to various stresses. In addition, phosphorylation can also activate some enzymes involved in starch synthesis and increase the physical interactions between those enzymes [30, 31].

In this study, we performed the first iTRAQ-based quantitative proteome and protein phosphorylation analysis during grain development of elite Chinese bread wheat cultivar Yanyou 361. Our results have revealed the central metabolism changes involved in wheat grain development and provide new insights into the grain development pathways, particularly for starch and protein synthesis as well as adverse defense mechanisms.

## Results

### Physiological changes of flag leaves during grain development

The dynamic changes of relative water content (RWC), total chlorophyll content, water soluble content (WSC), and proline content of flag leaf at five grain developmental stages (7, 14, 18, 21, and 28 DPA) of the Yanyou 361 cultivar are shown in Additional file 1: Figure S1. The results showed that RWC gradually decreased along with grain development, while total chlorophyll content increased from 14 to 18 DPA and then decreased dramatically until grain maturation. WSC and proline contents gradually increased during the grain developmental process.

### Grain and starch granule development

**Figure 1:**
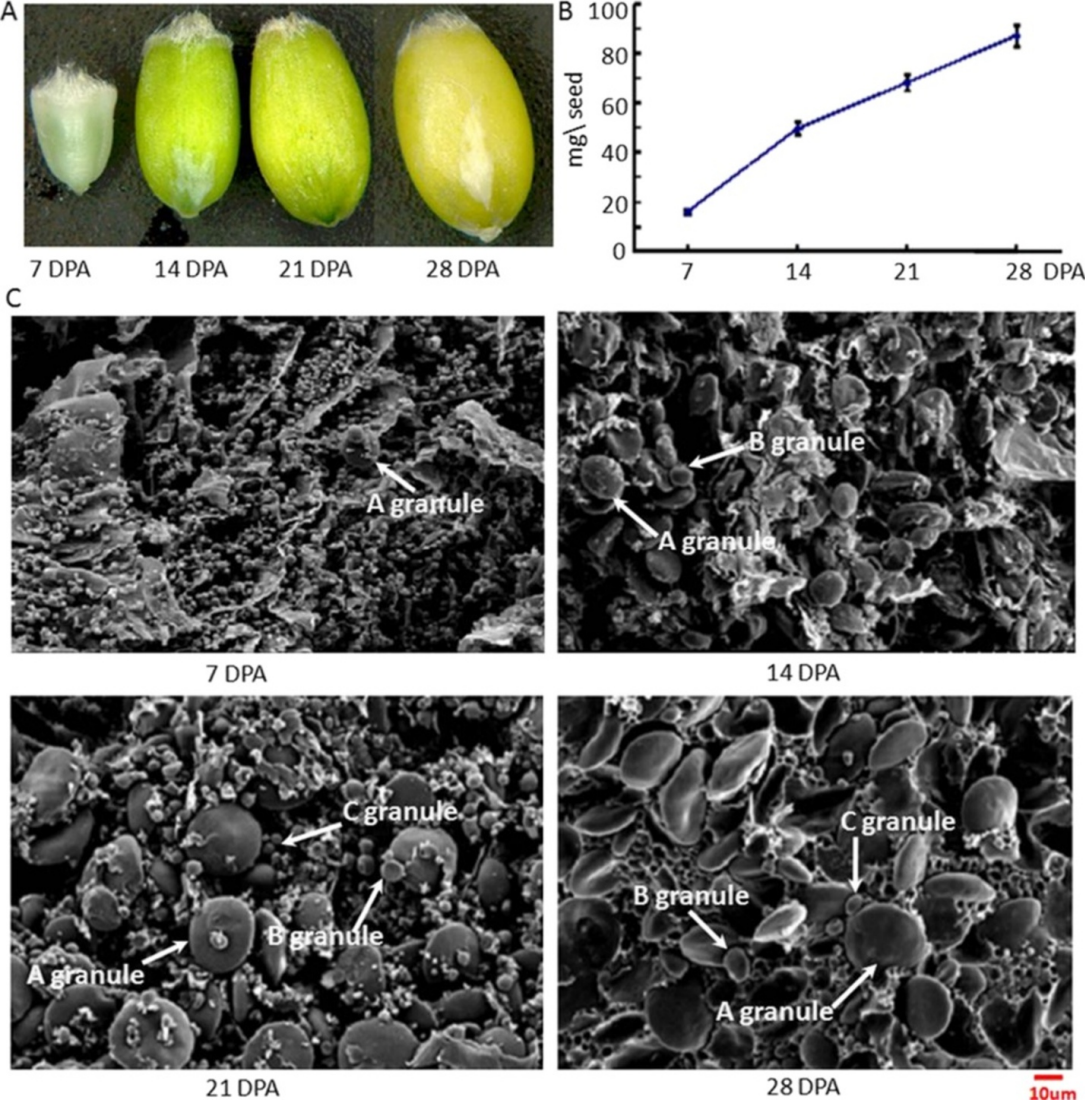
**Grain development during four stages after anthesis in bread wheat cultivar Yanyou 361. A**: Grain morphological development of four stages. **B**: Grain weight changes during grain development. **C**: SEM observation of starch granules from developing grains. The **A**, **B** and **C** starch granules are indicated and the red line represents 10 *μ*m.

Grain morphological changes and scanning electronic microscopy (SEM) images of starch granules during grain development in the Yanyou 361 cultivar are shown in Figure 1. Grain size increased gradually along with grain development (Figure 1A), and grain fresh weight increased by about 2-fold at 14 DPA compared to 7 DPA (Figure 1B). SEM observation indicated that the rapid increase in size and accumulation of A and B starch granules occurred in the early stages of grain development (Figure 1C). A large number of A-type starch granules occurred at 7 DPA, which rapidly enlarged at 14 and 21 DPA, with most of them reaching about 15–20 *μ*m in diameter at 28 DPA. The B-type starch granules were first observed at 14 DPA, and then enlarged slowly until grain maturation. The C-type granules with diameters less than 5 *μ*m were observed at 21 DPA and their size only slightly changed at 28 DPA (Figure 1C).

### Protein identification and quantification

In this study, iTRAQ-based quantitative proteome characterization during grain development of the Yanyou 361 cultivar was investigated to reveal the central metabolic proteins involved in wheat grain development. A global profiling of quantitative proteome was obtained on whole developing caryopses from 7, 14, 21, and 28 DPA using three biological replicates. Overall, more than 11,000 unique peptides (Additional file 2: Table S1), corresponding to 1,815 proteins were identified (Additional file 3: Table S2), of which 1,744 proteins with quantitative information were elicited. According to false discovery rate (FDR) <0.01 and unique peptide numbers ≥2, 1,164 non-redundant proteins were obtained (detailed information provided in Additional file 2: Table S1 and Additional file 3: Table S2). Before comparison analysis of proteins expression levels, Pearson correlation between three replicates was performed to determine the analytical reproducibility (Additional file 4: Figure S2). A 45°-diagonal line was obtained with little variation throughout the detection range, which demonstrates the expected distribution without obvious changes between the biological replicates.

### Protein expression profiles during different grain development

**Figure 2:**
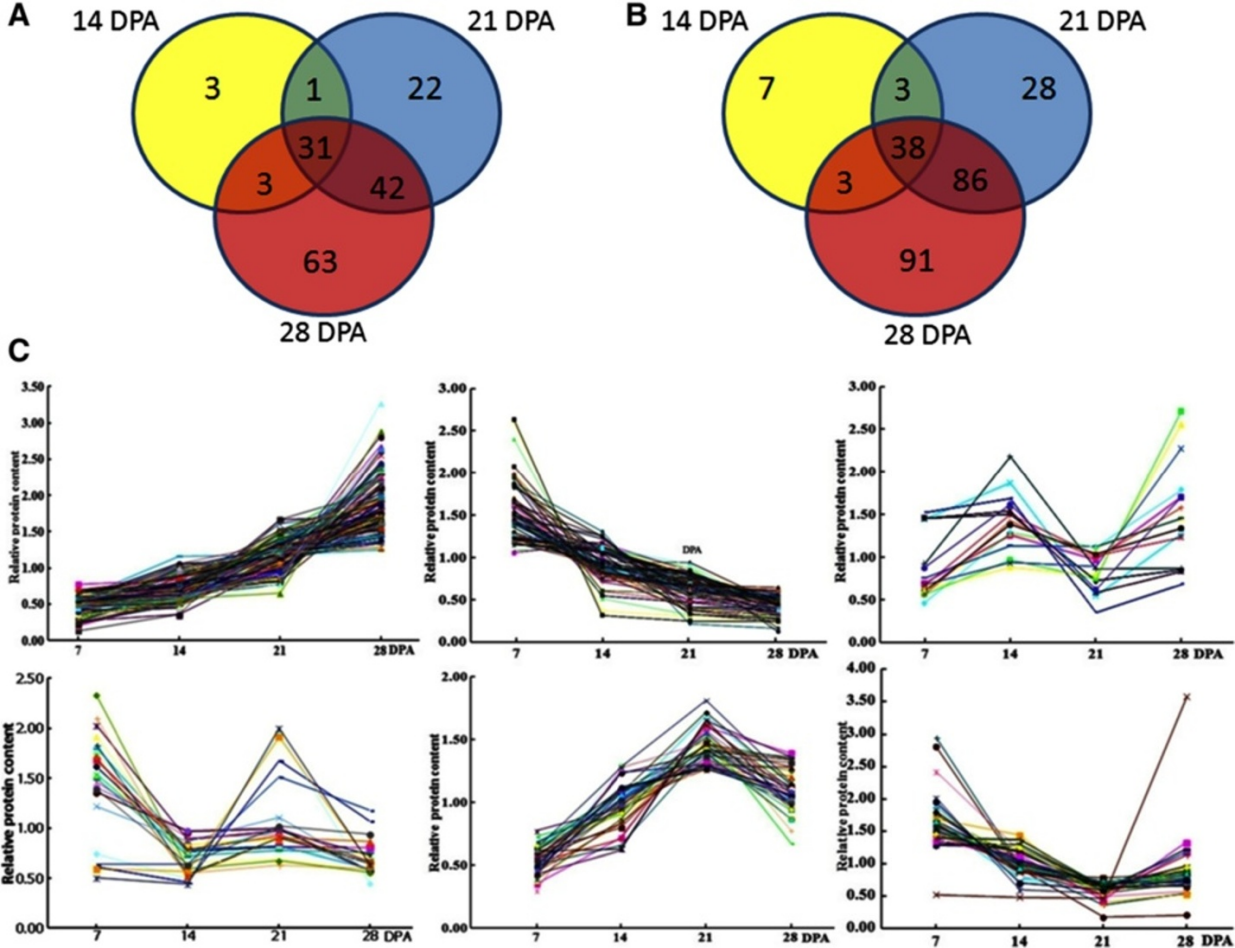
**Venn diagrams and expression of 421 DEPs identified by iTRAQ in wheat grains during different development stages.** The numbers of DEPs with down-regulation **(A)** and up-regulation **(B)** under given development stages are shown in different segments. Six distinct expression patterns of 421 DEPs at different development stages are shown in **(C)**.

A 2-fold cut-off was used to implicate significant changes in the abundance of differentially expressed proteins (DEPs) during grain development. Of 1,146 non-redundant proteins identified, 421 showed more than 2-fold changes (p ≤0.05) in protein expression level in at least one of the development stages and, therefore, they were identified as DEPs. Their detailed information is provided in Additional file 5: Table S3-A, and 10 representative protein MS spectra are shown in Additional file 6: Figure S3. The distributions of 421 DEPs and their overlapping during different grain developmental stages illustrated using the Venn diagram analysis are shown in Figure 2. A total of 165 DEPs were down-regulated under at least one development stage (Figure 2A), including 31DEPs at all development stages, 46 DEPs at two stages (one at 14 and 21 DPA, three at 14 and 28 DPA, and 42 at 21 and 28 DPA), while three, 22, and 63 DEPs particularly down-regulated at 14 DPA, 21 DPA, and 28 DPA, respectively. As indicated in Figure 2B, among 256 up-regulated DEPs, 38 DEPs were shared by all development stages, 92 DEPs shared by two stages (three at 14 and 21 DPA, three at 14 and 28 DPA, and 86 at 21 and 28 DPA), while 7, 28, and 91 DEPs particularly up-regulated at 14 DPA, 21 DPA, and 28 DPA, respectively.

In general, six clearly different expression patterns during grain development were generalized among 421 DEPs (Figure 2C): 180 DEPs (42.76%) in up-regulation, 88 (20.90%) in down-regulation, 70 (16.63%) in up- to down-regulation, 43 (10.21%) in down- to up-regulation, 24 (5.7%) in down- to up- to down-regulation, and 16 (3.8%) in up- to down- to up-regulation.

### Functional classification and sub-cellular localization of DEPs

**Figure 3:**
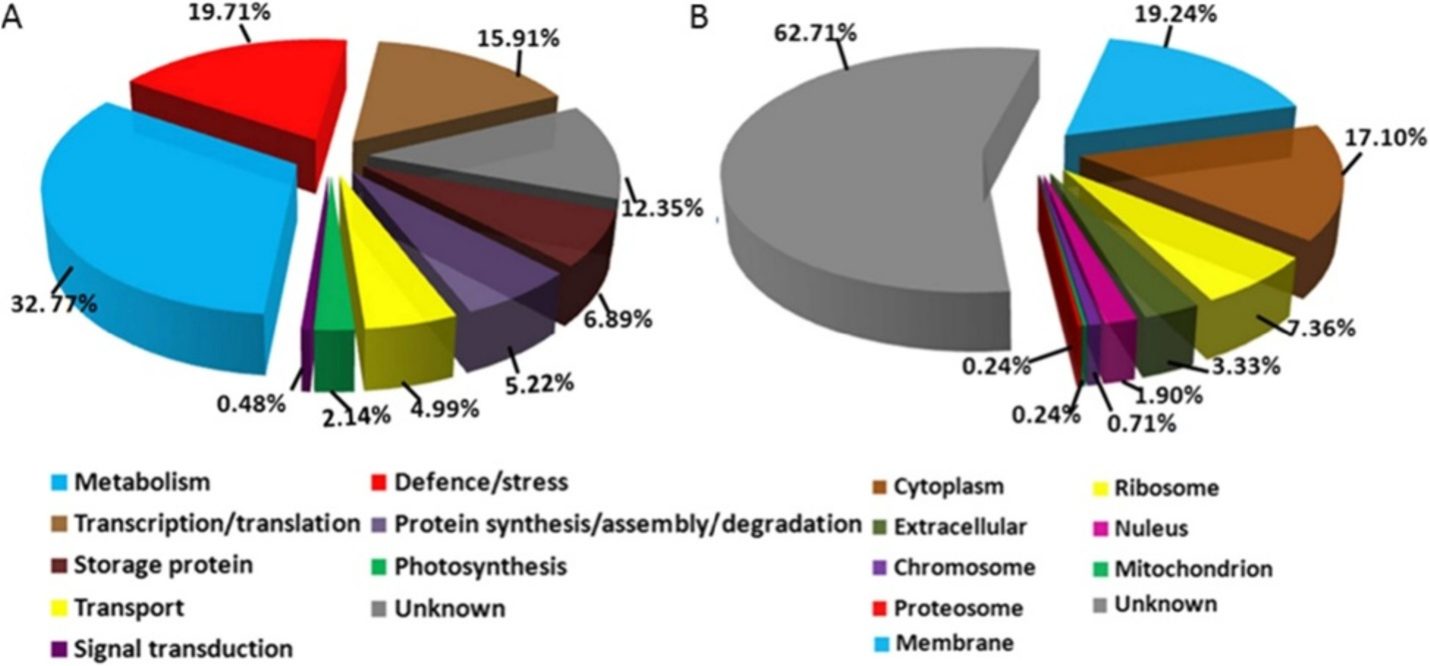
**Functional classifications (A) and sub-cellular localization (B) of 421 DEPs identified in the developing grains.**

According to the molecular functions listed on the UniProt and Gene Ontology website, the 421 DEPs were classified into nine functional categories (Figure 3A, Additional file 5: Table S3-A). These nine functional protein categories were involved in metabolism (32.77%), stress/defense (19.71%), transcription/translation (15.91%), storage proteins (6.89%), protein synthesis/assembly (5.22%), transportation (4.99%), photosynthesis (2.14%), signal transduction (0.48%), and unknown function (12.35%). A Fischer’s enrichment analysis was carried out on the subsets characterized by different expression patterns with those of the whole list of quantified proteins as reference list. As a result (Additional file 7: Figure S4A-C), "protein maturation" (GO:0051604, FDR: 1.50E - 6) was significantly over-represented in up-regulation subset, "translation" (GO:0006412, FDR: 1.9E - 10) was significantly over-represented in down-regulation subset, "biosynthetic process" (GO:0009058, FDR:2.5E - 3) and "cellular protein modification process" (GO:0006464, FDR:2.5E - 3) were significantly over-represented in up-to-down-regulation subset. There was no statistically significant result in other three subsets.

Subcellular localizations of the 421 identified proteins were predicted using Gene Ontology (Additional file 5: Table S3-A) and WoLF PSORT (Additional file 5: Table S3-B). As result showed in Figure 3B, 19.24% of DEPs were located in the plasma membrane, 17.1% in the cytoplasm, and 7.36% in the ribosome, but 62.71% were unknown.

### Differential protein expression during grain development

**Figure 4:**
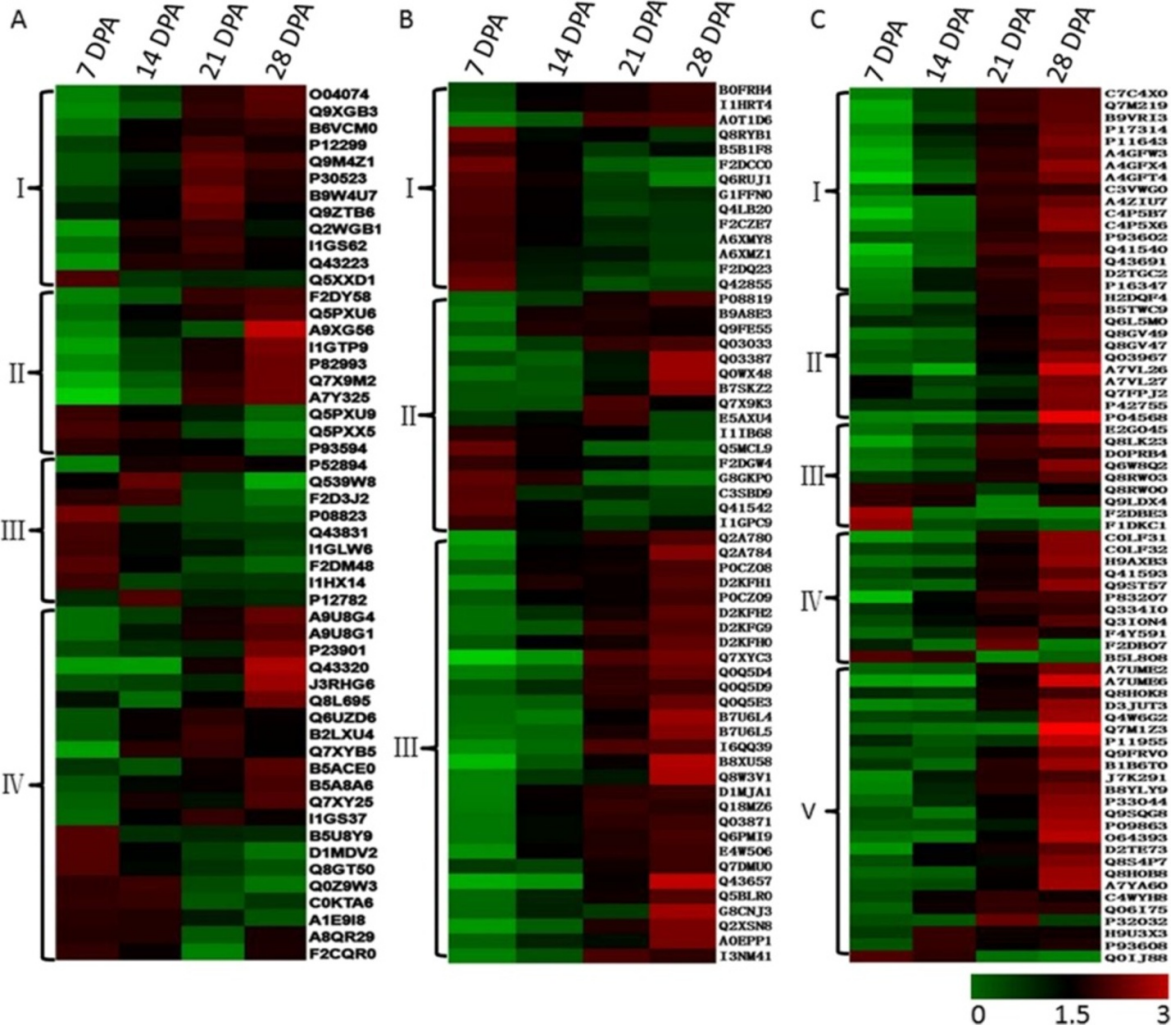
**Hirerarchical clustering analysis of DEPs with different functions during grain development. A**: DEPs involved in carbohydrate metabolism related proteins, including starch synthesis (A-I), beta-amylases (A-II), photosynthesis (A-III) and other carbohydrate metabolism (A-IV). **B**: DEPs involved in protein metabolism, including nitrogen metabolism (B-I), protein systhesis/assembly/degradation (B-II) and storage proteins (B-III). **C**: DEPs involved in defense and stress related proteins, including alpha-amylase inhibitor (C-I), LEA proteins (C-II), ROS scavenging related proteins (C-III), protein protection (C-IV) and other defense/stress related proteins (C-V).

Hierarchical clustering analysis (HCA) was performed to display the dynamic expression patterns of 184 DEPs involved in carbohydrate metabolism and photosynthesis (Figure 4A), protein metabolism and storage proteins (Figure 4B), and defense/stress (Figure 4C).

Totally, 52 DEPs involved in carbohydrate metabolism and photosynthesis were divided into four function groups (Figure 4A), including starch biosynthesis (I), beta-amylases (II), photosynthesis (III), and other carbohydrate metabolism (IV). In group I, 12 important enzymes were involved in starch biosynthesis, including five ADP-glucose pyrophosophorylases (AGPase: B6VCM0, P12299, Q9M4Z1, Q5XXD1, and P30523), two sucrose synthases (SuSy: I1GS62 and Q43223), four starch branching enzymes (SBE: O04074, Q9XGB3, B9W4U7, and Q9ZTB6), and one starch synthase (SS) II-D (Q2WGB1). Most of them showed a significant accumulation from 14 to 21 DPA. For example, three AGPases (P12299, Q9M4Z1, and P30523) were more than 2-fold up-regulated at 14 DPA, and had the highest expression level at 21 DPA. Two SuSy I1GS62 and Q43223 increased 1.9- and 2.9-fold at 14 DPA, respectively, and reached the highest level at 21 DPA. Starch synthase II-D (Q2WGB1) had a 2.92-fold expression level at 14 DPA and continuously increased to 21 DPA. However, starch branching enzyme (SBE) was initiated later, and 2-fold expression changes were observed after 21 DPA (Figure 4A-I and Additional file 5: Table S3-A). The group II included 10 beta-amylases related to the hydrolysis of starch, in which seven (F2DY58, Q5PXU6, A9XG56, I1GTP9, P82993, Q7X9M2, and A7Y325) displayed up-regulated expression during grain development and reached the highest accumulation level at 28 DPA, while three (Q5PXU9, Q5PXX5, and P93594) were down-regulated (Figure 4A-II). Nine proteins involved in photosynthesis, such as ribulose bisphosphate carboxylase (RuBisCO) large chain (Q539W8), small chain (F2D3J2) and phosphoglycerate kinase (PGK, P12782), which were generally downregulated, were placed into group III (Figure 4A-III). The remaining 21 DEPs involved in other carbohydrate metabolism processes were included in group IV, in which 9 showed apparently up-regulation, such as alcohol dehydrogenase ADH1A (A9U8G4), ADH2A (A9U8G1), and the others displayed up-down and down-regulation during grain development (Figure 4A-IV).

As shown in Figure 4B, the 59 DEPs involved in protein metabolism were divided into three functional groups, including nitrogen metabolism (I), protein synthesis/assembly/degradation (II), and storage proteins (III). The group I contained 14 DEPs involved in nitrogen metabolism, of which only three were up-regulated, including two aspartate aminotransferases (B0FRH4 and A0T1D6) and one porphobilinogen deaminase (Q8RYB1), and 11 of them were down-regulated, such as two glutamine synthetase (GS) (Q6RUJ1 and G1FFN0). In group II, 16 DEPs related to protein synthesis/assembly/degradation had different expression patterns; among them, five (P08819, Q03033, Q03387, Q0WX48, and B7SKZ2) demonstrated an up-regulated expression pattern and had the highest expression level at 28 DPA, four DEPs, including protein disulfide isomerases (PDI) (Q9FE55 and B9A8E3), elongation factor-1 alpha (Q7X9K3), and vacuolar processing enzyme 2a (E5AXU4), displayed up-down expression and had the highest level at the middle development stages, while the others were down-regulated. In group III, 29 storage proteins showed up-regulated expression patterns during grain development, particularly with a significantly higher expression level at later grain development stages, including five high molecular weight glutenin subunits (HMW-GSs), two low molecular weight glutenin subunits (LMW-GSs), five avenin-like a, three gliadins, seven globulins, and seven others. Seven of them were significantly altered with more than 5-fold changes at 28 DPA (Q2A780, Q2A784, Q7XYC3, B8XU58, Q8W3V1, Q43657, and Q2XSN8), of which 19 kDa globulin (Q7XYC3) increased by 15.22-fold and two LMW-GS (B8XU58 and Q8W3V1) increased by 12.65- and 5.93-fold, respectively. All five HMW-GS showed more than 3-fold increase at 28 DPA (Additional file 5: Table S3-A).

The 74 DEPs involved in stress/defense were divided into five functional groups (Figure 4C), including alpha-amylase inhibitors (αAI) (I), late embryogenesis abundant proteins (LEA proteins) (II), ROS scavenging system (III), protein protection (IV), and others (V). In group I and group II, 17 αAI and 11 LEA proteins had the highest expression at 28 DPA and 10 were more than 5-fold upregulated, including eight αAI (P11643, A4GFW3, A4GFX4, A4GFT4, C4P5B7, C4P5X6, Q41540, and Q43691) and two LEA proteins (A7VL26 and P04568). The group III contained nine DEPs involved in the ROS scavenging system, including up-regulated catalase (E2G045), peroxidase (Q8LK23), peroxiredoxin (D0PRB4), 1-Cys peroxiredoxin PER1 (1-Cys Prx), and glutathione transferase (GT) (Q8RW03), down-regulated catalase (F2DBE3 and F1DKC1), and down-up regulated thioredoxin (Q9LDX4) and GT (Q8RW00). The group IV included five serpin proteins (C0LF31, C0LF32, H9AXB3, Q41593, and Q9ST57), one chymotrypsin inhibitor WCI (CTI) (P83207), and five hot shock proteins (Hsps) (Q334I0, Q3I0N4, F4Y591, F2DB07, and B5L808), which were involved in protecting proteins from degradation. All the five serpins and CTI were up-regulated during grain development, and particularly serpins COLF31 and CTI (P83207) were up-regulated by 6.36- and 6.06-fold at 28 DPA, respectively. Among the five Hsps, Hsp 101 (Q334I0) and Hsp17.2 (Q3I0N4) were up-regulated and had 2- and 2.91-fold level changes at 28 DPA, respectively. Hsp90 (F4Y591) and Hsp10 (F2DB07) had the highest expression level at 21 DPA with 3.13- and 2.91-fold increase, respectively, after which they were sharply down-regulated. Hsp70 (B5L808) was sharply down-regulated by 3.54-fold at 21 DPA, and then showed a slight up-regulated expression (Additional file 5: Table S3-A). The group V had 26 DEPs, which were mainly involved in cell wall protection, insect and pathogen resistance, osmotic and cold stresses, among which 21 showed significant up-regulated expression and reached the highest expression level at 28 DPA (Additional file 5: Table S3-A).

### Transcriptional expression analysis as revealed by qRT-PCR

**Figure 5:**
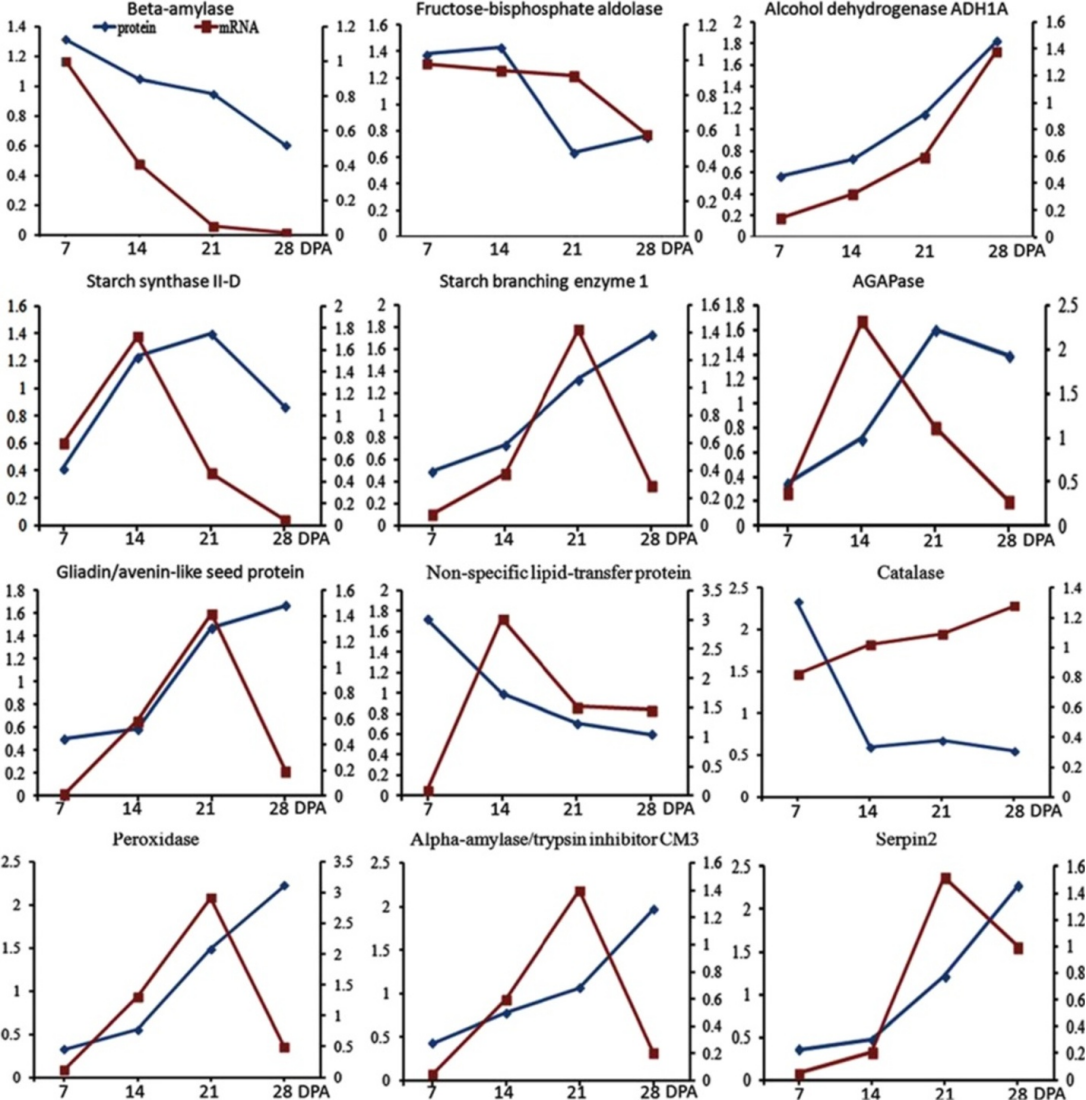
**Comparison of protein and mRNA expression partterns of 12 representative DEPs at four grain developmental stages (7, 14, 21, and 28 DPA) by iTRAQ and qRT-PCR.** Blue line represent protein expression pattern, red line represent mRNA expression pattern.

To provide further information of the correspondence between proteins and their mRNA expression patterns, quantitative real-time polymerase chain reaction (qRT-PCR) was performed to investigate the dynamic transcriptional expression patterns of 12 representative DEPs. The high amplification efficiency of each primer pair was determined by standard curves using serial 14 DPA cDNA. Dilutions and unique melting temperature peaks of each specific primer are shown in Additional file 8: Figure S5. As shown in Figure 5, three genes encoding beta-amylase, alcohol dehydrogenase ADH1A, and SS II-D displayed the same expression patterns with their protein levels, seven genes encoding SBE I, AGPase SSU, gliadin/avenin-like seed protein, non-special lipid transfer protein, αAI, serpin, and peroxidase showed similar expression patterns at three developmental stages with their protein expression patterns. However, two genes encoding Fructose Bisphosphate Aldolase and catalase showed opposed expression patterns with their proteins. Researches demonstrated that the mRNA level and protein abundance of fructose bisphosphate aldolase is not consistent due to translational or post-translational regulation [32], and the expression of catalase is regulated by post-transcriptional regulation [33]. These results are generally consistent with those of a previous report [18, 34].

### Phosphorylated proteins characterization during grain development

The strategy of TiO_2_ affinity chromatography combined with LC-MS/MS and MaxQuant software was used to identify phosphopeptides and phosphorylated sites in developing grains at 28 DPA. In total, 532 phosphopeptides with 598 phosphorylated sites belonging to 417 phosphoproteins were identified. Of the 417 phosphoproteins, 23 containing 29 phosphopeptides and 41 phosphorylated sites were also detected by iTRAQ. The detailed description of these 23 phosphoproteins and their expression patterns are shown in Additional file 5: Table S3-C. Twenty-nine representative MS spectra are shown in Additional file 9: Figure S6.

On the basis of the phosphorylation site probability, the 40 phosphorylation sites belonged to class I (*p* value ≥0.75), in which 37 serines and 3 threonines were phosphorylated. The biological functions of these phosphoproteins are mainly involved in carbohydrate metabolism (43.47%), defense and stress (39.13%), and protein synthesis (17.39%).

Among the 23 phosphoproteins identified, four DEPs including serpin-N3.2 (H9AXB3), serpin-Z2A (Q9ST57), and heat shock protein 90 (F4Y591) contained four phosphorylation sites; two DEPs: LEA1 protein (Q8GV49), wail7 (E0WC53) had three phosphorylation sites; five DEPs including AGPase (P12299), phosphorylase (Q6UZD6), LEA3 protein (Q8GV47), serpin 3 (C0LF32), and serpin-Z1A (Q41593) contained two phosphorylation sites; and the remaining phosphoproteins contained one phosphorylation site (Additional file 5: Table S3-C). In particular, five DEPs related to starch biosynthesis were found to be phosphorylated, including 4 AGPase (B6VCM0, P12299, Q9M4Z1, and Q5XXD1) and one SuSy (Q43223). In addition, three LEA proteins (Q8GV49, Q8GV47, and Q03967), four serpins (C0LF32, H9AXB3, Q41593, and Q9ST57), and Hsp90 (F4Y591), which participate in defense pathways and other various biological processes, were also phosphorylated.

**Figure 6:**
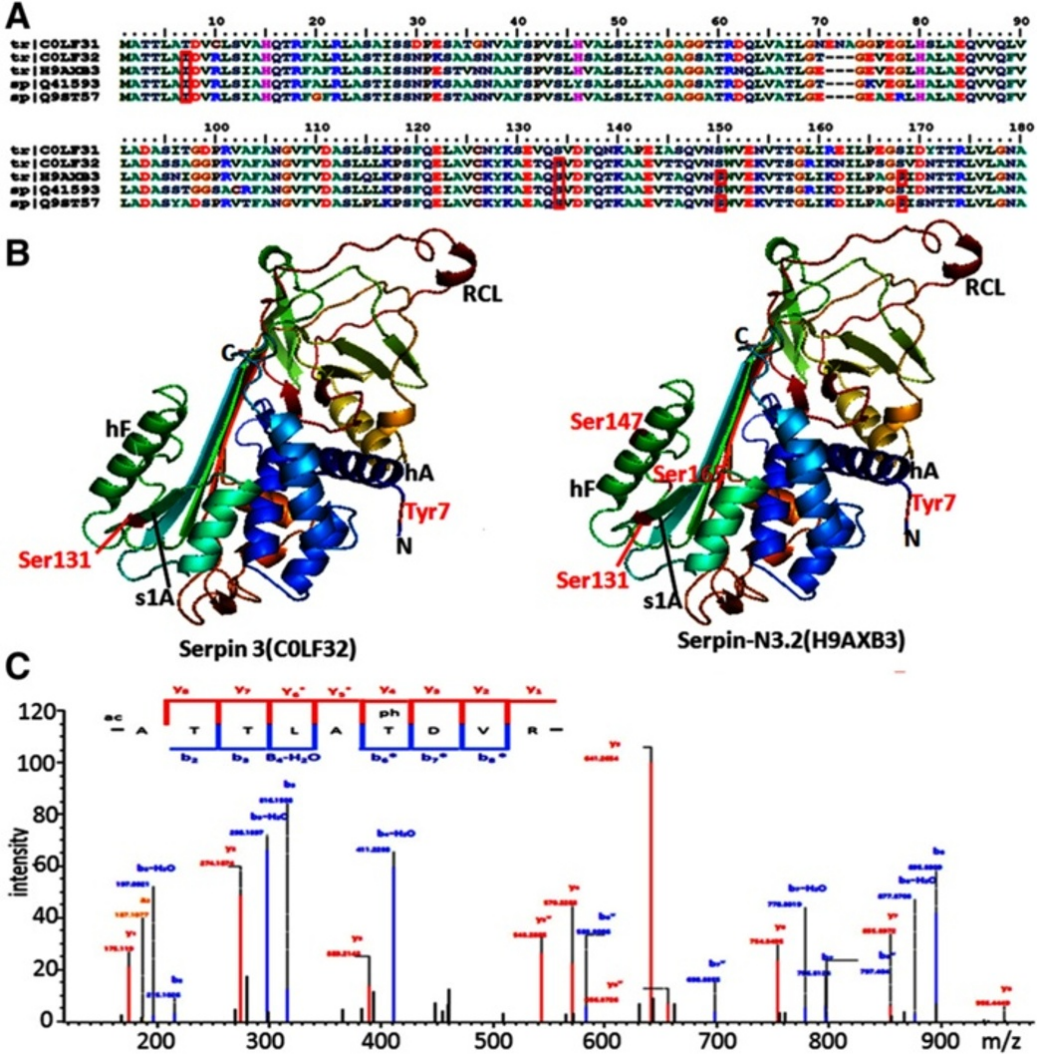
**Phosphorylation modifications of serpins in the developing grains of Yanyou 361. A**: The part sequence alignment of serpins identified in the current study, and the red box indicated the phosphorylated sites. **B**: Three-dimensional structure map of serpin3 (C0LF32) and serpin-N3.2 (H9AXB3) predicted by Phyre. The phosphorylated sites Thr7, Ser131, Ser147 and Ser165 are marked with red color, and a part of domains are marked. **C**: The representative MS/MS spectra of the common phosphopeptide _ATTLAT(ph)DVR_ present in four serpins.

The partial sequence alignment of five serpins (C0LF31, C0LF32, H9AXB3, Q41593, and Q9ST57) showed few amino acid differences (Figure 6A). Both C0LF32 and Q41593 contained two conserved phosphorylation sites at Thr7 and Ser131, while the other two serpins (H9AXB3 and Q9ST57) had four phosphorylation sites at Thr7, Ser131, Ser147, and Ser165, suggesting that these phosphorylation sites were highly conserved in the serpin family. The 3D structures of serpin 3 (COLF32) and serpin-N3.2 (H9AXB3), which were predicted using Phyre [35], are shown in Figure 6B. The phosphorylation sites at Thr7, Ser131, and Ser147 were located at the N*-*termini, β-sheets s1A, and α-helices hF, marked according to Fluhr *et al.*[36], respectively. One representative MS/MS spectra map of the common phosphopeptide _ATTLAT(ph)DVR_ present in the four phosphorylated proteins is shown in Figure 6C and the phosphorylation site Tyr7 is indicated.

### Protein-protein interaction analysis of DEPs

**Figure 7:**
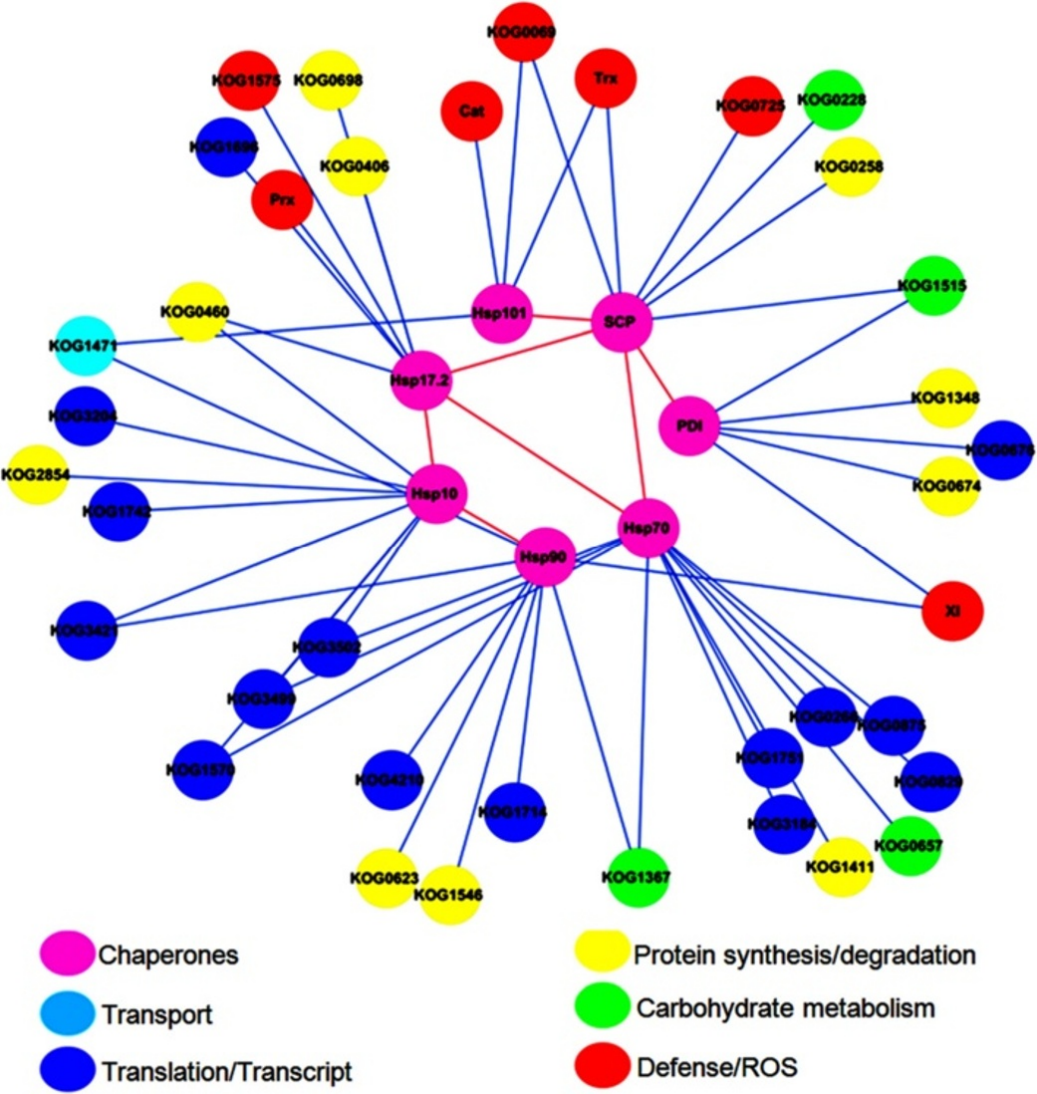
**Interaction network of some important chaperone DEPs (PDI, SCP and Hsp) identified by iTRAQ.** This network shows only known and direct interactions among identified DEPs.

The protein-protein interaction (PPI) network of the DEPs identified in the current study was analyzed using STRING. In total, 147 euKaryotic Orthologous Groups (KOGs) representing 173 DEPs involved in seven functional categories were used to construct the PPI network (Additional file 5: Table S3-B). To improve the reliability of the PPI analysis, the confidence score was set to the high level (≥0.900). As shown in Additional file 10: Figure S7, a complex PPI network contained 79 nodes and 278 edges, was displayed through Cytoscape. Seven chaperones involved in protein synthesis/assembly/degradation, defense/stress, and other important functions were identified by iTRAQ analysis, which included five Hsps (KOG1051, KOG0710, KOG0019, KOG0101, and KOG1641), one SCP (KOG1282), and one PDI (KOG0190). They were used to further extract the potential interacting proteins from the whole PPI network, and a chaperones-centered sub-network was constructed (Figure 7), which indicated a complicated interaction network among chaperones and other DEPs. A total of 36 KOGs representing 58 proteins (Additional file 5: Table S3-C) involved in transcription/translation, protein synthesis/assembly/degradation, defense/stress, and carbohydrate metabolism and transport could interact with the chaperones detected in this work. In particular, 10 KOGs were essential proteins involved in protein synthesis, such as elongation factor Tu (KOG0460) and aspartate aminotransferase (KOG1411), and 7 DEPs were important for defense/stress and ROS scavenging system, such as Cat, Prx, XI, GT, and TRX.

## Discussion

Wheat endosperm is an allohexaploid tissue in which 80–85% of the extractable proteins are gliadins and glutenins. The albumins and globulins of wheat endosperm have received relatively little attention because of their low abundance and perceived secondary role in flour quality. In the current study, iTRAQ-based quantitative proteome analysis detected the dynamic changes of 421 DEPs at four developmental stages of wheat grains, including considerable albumins and globulins that are generally expressed at low levels. The identified DEPs were much more numerous than those discovered in previous reports with traditional 2-DE and 2D-DIGE approaches [10, 15]. These DEPs were mainly involved in starch and protein synthesis as well as defense/stress; in particular, some functionally important proteins were phosphorylated, suggesting their key roles in grain development.

### Starch biosynthesis and accumulation during grain development

As one of the two major wheat grain store components, starch constitutes approximately 70% of mature grain dry weight. Starch is synthesized and accumulated by the coordinated roles of a group of enzymes whose activities are positively correlated to starch accumulation rate [37]. In that precious work, only a limited number of starch synthesis enzymes were detected in developing wheat grains using the traditional 2-DE method [10, 17]. Through the iTRAQ-based quantitative proteomic approach used in this study, considerable key enzymes related to seed starch biosynthesis were identified, and 72 (17.1%) DEPs among 421 were located in the cytoplasm and related to starch metabolism (Figure 4B), thus providing a comprehensive view of starch biosynthesis during wheat grain development.

It is generally accepted that the grain-filling rate in cereals is mainly determined by sink strength, which could be increased by the high activity of SuSy [38, 39]. Two SuSys were found to be up-regulated and reached the highest level at 21 DPA, which is similar to the change of chlorophyll content (Addition file 1: Figure S1). As the senility and photosynthetic rate declined of flag leaves, the supply of sugars to grain decreased, which could led to down-regulation of SuSys. At early grain stage, photosynthesis provides the raw material triosephosphate for starch biosynthesis, and plays a role in maintaining the endogenous O_2_ balance [40]. Our results showed that ribulose bisphosphate carboxylase (RuBisCO) and phosphoglycerate kinase (PGK), which are essential for photosynthesis, had higher expression levels at early grain development stages, and reached the highest levels at 14 DPA (Additional file 5: Table S3-A), which would promote plant photosynthesis and grain filling.

Grain development is the process of starch biosynthesis and accumulation in endosperm cells. Sucrose is constantly split into UDP-glucose by SuSy and used for starch synthesis. Starch biosynthesis is initiated with a substrate of ADP-glucose (ADP-Glu) formed by AGPase, which is regarded as the rate-limiting enzyme in starch biosynthesis [41]. The ADP-Glu is transported into the cytoplasm by ADP-glucose brittle-1 transporter (BT1) [42]. Different classes of SS are recruited to elongate the glucan chain, including SS I–IV and granule bind starch synthase (GBSS), while SBEs are involved in forming alpha-1,6-glucoside and DBE hydrolyzes α-(1,6)-linkages within a polyglucan [43]. Almost all the enzymes related to starch synthesis were detected in our study, except for GBSS and DBE; most of these enzymes showed the highest expression level at 21 DPA, except for AGPase small subunit (Q5XXD1), which was down-regulated, and SBEI, which showed the highest expression level at 28 DPA. This is consistent with the rapid increase of grain weight and starch granules (Figure 1).

Recent studies have showed that starch biosynthesis is mainly regulated by protein post-translational modifications, especially by phosphorylation [30]. Previous studies proved that the wheat starch synthesis enzymes such as SSI, SSII-a, SBEI, SBEII-a, and SBEII-b could be phosphorylated and participate in protein-protein interactions, and their activity could be enhanced by phosphorylation [30, 31]. Some of the enzymes involved in starch biosynthesis have been found to form protein complexes such as SS-SBE complexes [30, 31] and AGPase-starch phosphorylase complexes [30]. Particularly, starch synthase catalytic domain is mainly responsible for glucan-substrate recognition and affinity [44]. Thus, the phosphorylation sites in the starch synthase catalytic domain may play an important role in recognizing and attracting glucan substrates. Previous reports found that many proteins related to starch metabolism were phosphorylated, such as at Ser-421 in AGPase small subunit, Thr-231 in AGPase large subunit, and Thr-330/548 and Ser-543/544 in SS III [45, 46]. In the current study, two AGPase large subunits (B6VCM0 and P12299), two small subunits (Q9M4Z1 and Q5XXD1), one SuSy (Q43223), and BT1 (D0EY60) were found to be phosphorylated in the developing grains (Additional file 5: Table S3-C).

**Figure 8:**
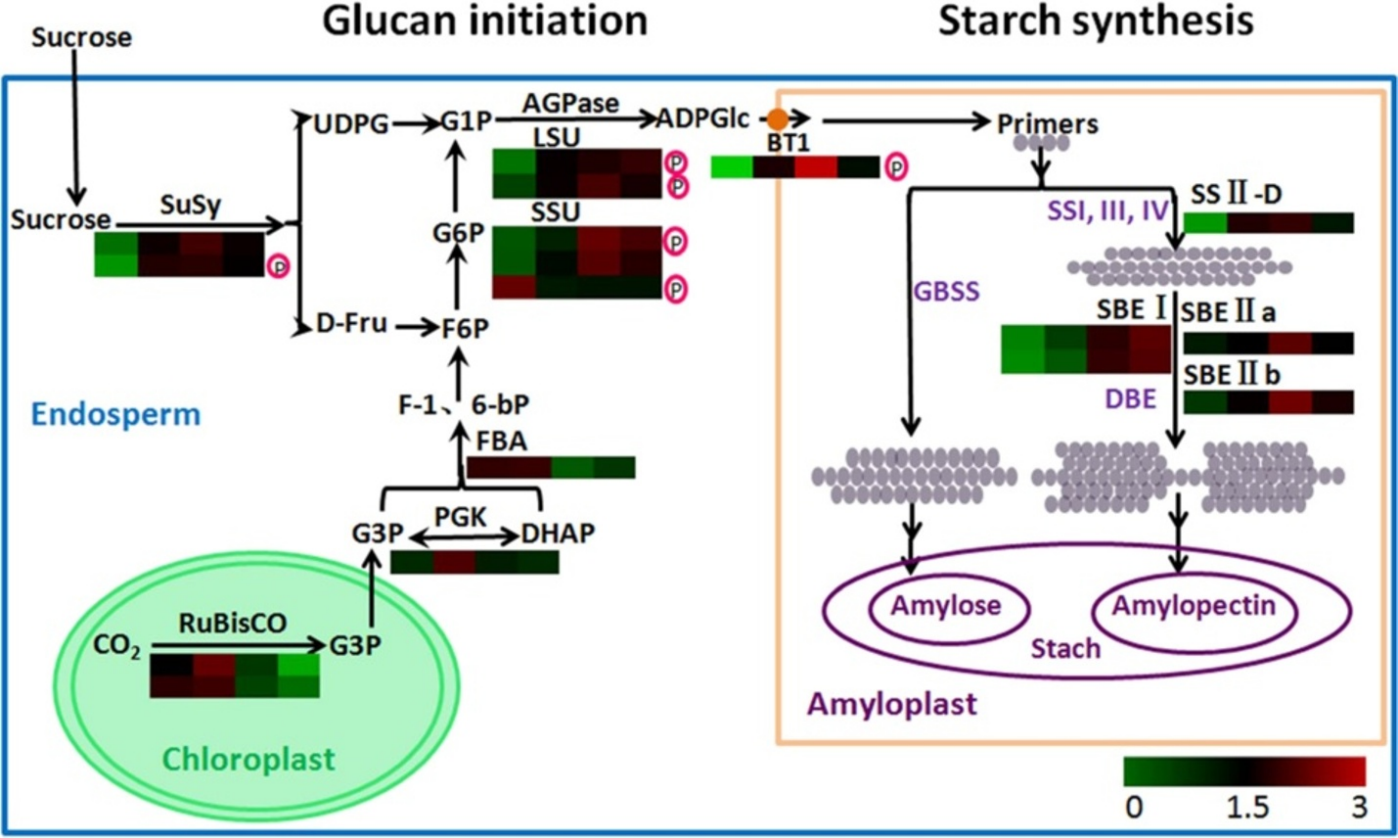
**An overview of the starch biosynthesis processes in wheat endosperm.** The starch biosynthesis includes sucrose degradation pathway, photosynthesis pathway as the raw materials source and starch biosynthesis pathway, and consists of two distinct phases: the glucan initiation process and the starch amplification process. ADPG is mainly synthesized by the cytosolic AGPase SSU and LSU, or supplied by sucrose degradation. The subsequent mechanisms underlying the glucan initiation process remain to be established. Branched dextrins are putatively processed by the coordinated activities of SS, BE, and/or DBE to produce the prototype of an amylopectin cluster structure, which further develops into amylopectin to establish the basic structure. Amylose is mainly synthesized by GBSS. Two AGPase SSU, two AGPase LSU and one Susy were phosphorylated. The protein levels are shown in coloured squares, indicating the change of expression for each developmental stage.

Our results provided dynamic changes and phosphorylated protein characterization of the DEPs involved in starch synthesis (Figure 8). Starch biosynthesis includes two main stages: glucan initiation and starch synthesis. Firstly, photosynthesis and sucrose hydrolysis provide the raw materials that are transformed into glucose-1-phosphate (G1P) by a series of enzymes; then, G1P is catalyzed into ADP-Glu by AGPase. ADP-Glu is carried to amyloplasts by BT1 and begins starch synthesis. ADP-Glu is elongated by GBSS and SS I-IV for amylase and amylopectin synthesis, respectively. SBE and DBE are involved in catalyzing the formation of α-(1, 6)-linkages within the polymers and hydrolyzing α-(1, 6)-linkages within a polyglucan to regularize the branching and maintain amylopectin crystallinity. Most of the enzymes involved in starch biosynthesis were identified in our work and showed a peak expression at 21 DPA, well consistent with the rapid increase of grain sizes and starch granules at 14–21 DPA (Figure 1). Some DEPs essential for starch biosynthesis were phosphorylated in the developing grains, which could increase the interaction between starch synthesis enzymes and enhance their activity to promote starch biosynthesis.

### Protein synthesis and accumulation during grain development

Cereal grains provide 50% of the dietary protein for humans and represent 70% of the protein intake for people in developing countries [47]. Therefore, nitrogen assimilation and protein synthesis are very important for wheat grain development. Previous research attested the key role of glutamine synthetase (GS) in plant nitrogen metabolism [48]. GS is responsible for the first step of ammonium assimilation and transformation into glutamine and glutamate, which are essential compounds in the amino acid biosynthetic pathway. Cytosolic GS has multiple metabolic functions such as assimilating ammonia into glutamine for transport and distribution throughout the plant. Recent studies have highlighted the important roles of GS1 cytosolic isoenzymes for nitrogen management linked to yield establishment and seed filling in monocotyledonous crops [49, 50]. Our study demonstrated that two GS proteins (Q6RUJ1 and G1FFN0) showed a higher expression level at 7 DPA (Additional file 5: Table S3-A), which could contribute to glutenin synthesis and improve gluten quality in the Yanyou 361 cultivar.

Protein disulfide isomerases (PDIs) are involved in the formation of inter- or intramolecular disulphide bonds and in the assembly of glutenin macropolymer (GMP) through intramolecular disulphide bonds among wheat grain proteins [51]. Thus, they play important roles in the maturation of secreted plasma membrane and storage proteins [52]. Investigations of rice mutant esp2 indicated that OsPDIL1-1 retains proglutelin to prevent heterotypic interactions with prolamine polypeptides within the ER lumen [53]. Peak expression of GmPDIL-3a and GmPDIL-3b during seed maturation suggested that they might be involved in folding or accumulation of storage proteins [54]. In wheat, nine PDI and PDIL genes were cloned, and their transcriptional levels in endosperm cellularization demonstrated that they were associated with storage protein synthesis and deposition, which is highly related to gluten quality [54]. In this study, iTRAQ-based analysis revealed that two PDI proteins (B9A8E3 and Q9FE55) were highly expressed at the early grain development stage (7–14 DPA) (Additional file 5: Table S3-A), which could facilitate the folding and maturation of storage proteins and promote gluten quality conformation.

Storage proteins included glutenins and gliadins as well as HMW albumins and globulins and avenin-like proteins, which have important impacts on grain nutrition and processing quality. Glutenins, which consist of high- and low-molecular weight glutenin subunits (HMW-GS and LMW-GS), are conserved storage proteins that are synthesized on polyribosomes attached to the rough endoplasmic reticulum (ER) [55]. Disulfide bond conformation, folding, and maturation of glutenin proteins are assisted by ER lumenal proteins such as molecular chaperone proteins PDIs, binding proteins (BiPs), and glutamine synthetases [56]. In the current work, five HMW-GS (1Ax1, 1Bx17 + 1By18, and 1Dx5 + 1Dy10) in the Yanyou 361 cultivar were detected by SDS-PAGE (Additional file 11: Figure S8), and this combination of HMW-GS is considered to yield the best dough quality [57]. All five subunits, which had fast accumulation from 14 to 21 DPA and whose peak expression occurred at 28 DPA, were identified by iTRAQ approach (Additional file 5: Table S3-A). Several studies demonstrated higher glutenin accumulation rate at the early stage of grain development, which contributed to good quality [58]. However, the expression levels of other storage proteins such as two LMW-GS (B8XU58 and Q8W3V1), five avenin-like proteins (Q2A780, Q2A784, P0CZ08, D2KFH1, and P0CZ09), and three gliadin/avenin-like proteins (D2KFH2, D2KFG9, and D2KFH0) only slightly changed until 21 DPA, but they were dramatically up-regulated at 28 DPA.

In addition, grain hardness in wheat is also important for end-product quality. Soft wheats are generally used to make cookies and pastries, while hard wheats are typically used to make breads [59]. Grain softness protein (GSP) and puroindoline are two important proteins controlling wheat flour hardness [60]. This study detected two up-regulated GSPs and three up-regulated puroindolines, the expression levels of which GSP (Q43657) and puroindoline b (Q2XSN8) had 10.51- and 6.28-fold changes, respectively (Additional file 5: Table S3-A), while the others had about 3-fold changes. Their high expression could contribute to the conformation of grain hardness and superior bread quality of the Yanyou 361 cultivar.

### The expression of defense/stress proteins in developing grains

To protect themselves against various biotic or abiotic stresses such as osmotic stress, drought, salts, extreme temperatures, and pathogenic microorganisms, a number of stress/defense proteins are expressed throughout wheat grain development. Previous studies indicated that 22% [15] and 25.6% [10] of grain albumins and globulins belonged to the stress/defense category. Our iTRAQ analysis revealed 83 (19.71%) stress/defense DEPs during the grain development of the Yanyou 361 cultivar, and some of them showed more than 5-fold up-regulation at later stages, such as LEA, αAI, XI, and serpin (Additional file 5: Table S3-A). LEA proteins accumulated at the late stage, especially under stress conditions such as drought and low temperature [61]. A previous study showed that ERD14, an *Arabidopsis* dehydrin (group 2 LEA protein), had calcium binding activity and it was regulated by phosphorylation, suggesting a role in calcium binding [62]. Calcium binding proteins may have a significant impact on signaling processes and regulate second messenger transmission [63]. In this study, eight LEA proteins involved in protecting seeds from serious dehydration at the late developmental stage were found to significantly increase their levels at 28 DPA (Additional file 5: Table S3-A). Particularly, the two LEA proteins Q8GV49 and Q8GV47 were phosphorylated at Ser4/Ser112/Ser116 and at Ser4/Ser112, respectively (Additional file 5: Table S3-C). Early-methionine-labeled polypeptides (EmPs) are members of the LEA proteins and are considered to be embryo-specific [64]. Three EmPs (Q7FPJ2, P42755, and P04568) were found to be dramatically up-regulated at 28 DPA, and two (P42755 and P04568) were phosphorylated at Ser32 and Ser30, respectively. These phosphorylation modifications may enhance the defense capability to various adverse stresses during grain development.

The serpin family functions through irreversible inhibition of endogenous and exogenous proteinases, which play important roles in plant growth, development, stress responses, and defense against insects and pathogens [65]. Serpins, which are found in wheat grain up to several percent of total protein, are probably laid down in seeds as a defensive shield to protect storage proteins from digestion [66]. Previous research demonstrated that serpin was significantly expressed under osmotic conditions such as salt and cold stresses [67]. In our study, the expression levels of five serpins (C0LF31, C0LF32, H9AXB3, Q41593, and Q9ST57) were dramatically increased at 28 DPA, and the level of C0LF31 increased by 6.36-fold (Additional file 5: Table S3-A). Furthermore, four serpins (C0LF32, H9AXB3, Q41593, and Q9ST57) were found to be phosphorylated (Additional file 5: Table S3-C) and all had one phosphorylated site at Thr7 in the N*-*terminus (Figure 6). Recent studies have demonstrated that the phosphorylation of tyrosine in maspins, which belong to the serpin family, plays a role in increasing maspin expression and accumulation in the cytoplasm [68]. The phosphorylation at Thr24 at the C-terminal 26-residue peptide can significantly increase the activity of serpin A1 [69]. Thus, phosphorylation modifications could increase the activity of serpins, then promote the formation of serpin-protease complex, and protect storage proteins from digestion.

The Hsp family has two essential ATP-binding sites and belongs to a larger class of AAA^+^ chaperone-like ATPases, which are involved in the assembly, operation, or disassembly of protein complexes [70]. Thus, Hsps play an important role in a diverse array of cellular processes such as amino acid and protein metabolism, stress response, and signal transduction [71]. Some members of the Hsp/chaperone family, such as Hsp17.2, Hsp10, and Hsp70, can stabilize protein conformation, prevent aggregation, and thereby maintain the non-native protein in a competent state for subsequent refolding by other Hsps/chaperones such as Hsp60, Hsp70, and Hsp90 [72]. Five Hsps were detected with different expression patterns during grain development, among which Hsp101 (Q334I0) and Hsp17.2 (Q3I0N4) were gradually up-regulated, Hsp90 (F4Y591) and Hsp10 (F2DB07) were sharply up-regulated to 21 DPA and then dramatically down-regulated, and Hsp70 (B5L808) was sharply down-regulated at 21 DPA (Additional file 5: Table S3-A). This suggests that different classes of Hsps may be involved in different functions through various expression patterns. Particularly, Hsp90 (F4Y591) was phosphorylated at four phosphorylation sites (Ser98, Ser239, Ser246, and Ser250), and the phosphorylated peptide _EIS(ph)DDEDEE_ was also identified in *Arabidopsis*[73] and rice [74], indicating the revolutionary conservation at this serine phosphorylation site in different plant species. Cooperation with different classes of Hsps under abiotic stress is essential in cellular protective functions such as maintaining proteins conformations, preventing aggregation of non-native proteins, refolding of denatured proteins, and removing harmful polypeptides [72]. Phosphorylation at the N-terminal domain of Hsp90 influenced client protein maturation and co-chaperone binding [71]. Protein-protein interaction analysis showed that Hsp90 (KOG0019) interacted with other 10 DEPs identified by iTRAQ, including Hsp10 (KOG1641) and the defense protein XI (KOG1339) (Figure 7). The phosphorylation of Hsp90 may enhance the interactions with other DEPs and promote its defense functions.

Exposure of plants to abiotic and biotic stresses can induce the production of ROS, including superoxide radicals (O_2_^.–^), alkyl-hydroperoxide (ROOH), and hydrogen peroxide (H_2_O_2_). To protect themselves against these toxic oxygen intermediates, plants employ defense systems, in which some defense proteins such as superoxide dismutases, catalases, thioredoxin, and peroxiredoxin catalyze the scavenging of ROS. Catalase is involved in the elimination of H_2_O_2_[75], peroxidase and peroxiredoxin reduce the levels of H_2_O_2_, alkyl-hydroperoxides, and OH^-^[76]. Thioredoxin is involved in cellular protection against oxidative stress, particularly during seed desiccation [77]. 1-Cys-peroxiredoxin has peroxidase activity when coupled to the thioredoxin system [78] and it can be activated by glutathione transferase (GT) [79]. In the present study, nine DEPs involved in ROS scavenging system were identified, of which five were gradually up-regulated during grain development, including catalase (E2G045), peroxidase (Q8LK23), peroxiredoxin (D0PRB4), 1-Cys-peroxiredoxin (Q6W8Q2), and GT (Q8RW03). Particularly, peroxidase was up-regulated by 6.78-fold at 28 DPA. Two catalases (F2DBE3 and F1DKC1) were down-regulated, while thioredoxin (Q9LDX4) and GT (Q8RW00) showed down-up expression pattern. Different expression patterns may reflect different functions in response to different stress conditions at different grain developmental stages. Interestingly, thioredoxin expression was sharply increased by 2.99-fold at 28 DPA (Additional file 5: Table S3-A), suggesting its role in the scavenging of toxic oxygen produced by dehydration at late grain developmental stages [77].

**Figure 9:**
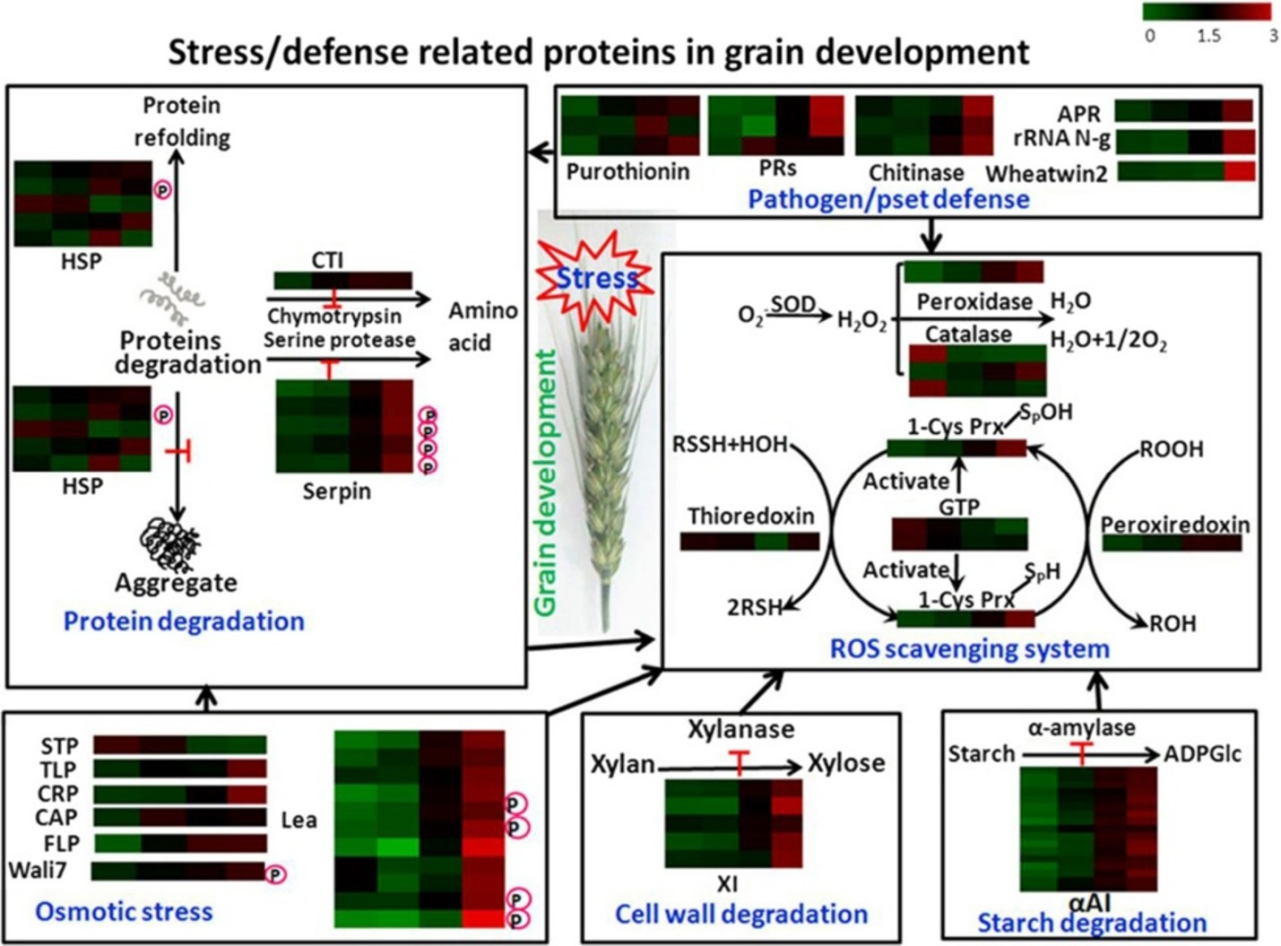
**An overlook of stress/defense related proteins and ROS scavenging system involved in wheat grain development.** LEA, TLP, SLP, CAP, CRP Emp and wail17 have coordinating role in various osmotic stresses during grain development. αAI and XI are mainly involved in protection of starch and cell wall, respectively. Purothionin, antifungal protein R (APR), chltinase, rRNA N-glycosidase (rRNA N-g) and wheatwin2 were mainly involved in resistance to pathogen and pest infection. Serpin, CTI and Hsps are involved in protein protection processes, serpin and CTI prevent protein from degradation by protese, Hsps prevent degraded protein from aggregation and help misfolded protein refolding, these processes also involved in resistance to pathogen/insect and osmotic stress. All defense process can produce reactive oxygen and which can be scavenged by ROS scavenging system. The protein levels are shown in coloured squares, indicating the change of expression for each developmental stage. The phosphorylated DEPs are marked.

Based on our results, an overview of DEPs involved in stress/defense could be drawn (Figure 9), which depicts a coordinated adverse response and defense mechanism during grain development. Since the grain development is liable to affected by adverse environments such as various biotic and abiotic stresses, various defense proteins were recruited to resist and adapt to the adverse environments. Considerable DEPs involved in defense/stress processes were activated and significantly up-regulated during grain development. Moreover, some DEPs such as LEA, serpins, Hsp90, and wail7 were phosphorylated, which could enhance the activity of stress/defense-related proteins and their interactions with other proteins. The coordinated functions of these stress/defense-related proteins could protect grain development from adverse harm, and benefit wheat yield and quality.

## Conclusion

Our results showed that the iTRAQ-based quantitative proteome analysis is a powerful technique for investigating the DEPs involved in wheat grain development, especially for low-abundance proteins. Overall, 421 DEPs and their quantitative expression characterization during the grain development of the Yanyou 361 cultivar were studied, which revealed the central metabolism changes involved in wheat grain development. Considerable DEPs involved in starch and protein biosynthesis pathways and stress/defense mechanisms displayed significant up-regulated expression, consistent with grain size increase and reserve accumulation during grain development. Some DEPs such as AGPase, SuSy, Hsp90, and serpins were phosphorylated at different sites, suggesting their important roles in wheat grain development. Our results have provided comprehensive proteome insights into the wheat grain development and increased our understanding of the molecular mechanisms influencing wheat yield and quality.

## Methods

### Wheat materials, field planting and sampling

Elite Chinese bread wheat cultivar Yanyou 361 (*Triticum aestivum* L*.*) was used as material, and grown in Wuqiao experimental station of China Agricultural University, Hebei province, located at 116°37′23″E and 37°41′02″N during 2011-2012 wheat growing season. Basal fertilizers in 0–20 cm soil layer included 0.8-1.4 mg/kg organic fertilizer, 0.04–0.1 mg/kg total nitrogen, 35–70 mg/kg alkali-hydrolyzable nitrogen, 10.8–30 mg/kg phosphorus and 90–110 mg/kg potassium. Before sowing, 200 kg/hm^2^ urea, 400 kg/hm^2^ (NH4)_2_HPO_4_, 150 kg/hm^2^ K_2_SO_4_ and 15 kg/hm^2^ ZnSO_4_ were fertilized. In the experimental location, average annual sunshine was 2690 hours, average annual temperature was 12.6°C, and the annual precipitation in 2011–2012 growing season was 153.9 mm. Yanyou 361 was planted in three biological replicates and each blot was 50 m^2^. As local field cultivation management, wheat plants were watered at jointing and flowering stages with 750 m^3^/hm^2^, respectively. Developmental grains at different development stages (7, 14, 21, and 28 DPA for iTRAQ analysis and 7, 9, 11, 14, 18, 21, 26, and 28 DPA for RNA extraction) were collected from three biological replicates and stored at -70°C prior to analysis.

### Scanning electron microscope (SEM)

Grain endosperms sampled from different developmental stages before mature (7, 14, 21, and 28 DPA) were fixed in the solution containing 5 ml 70% ethanol, 5 ml formalin, and 5 ml glacial acetic acid for at least 24 h. Samples were dehydrated sequentially in ethanol solutions of various concentrations including 70% (20 min), 80% (20 min), 90% (overnight) and 100% (20 min). After dehydration, samples were treated in 15 min steps in ethanol and isoamyl acetate mixtures with sequential ratios of 3:1, 1:1 and 1:3, and then soaked in isoamyl acetate before critical point drying. Treated grain samples were broken in half and sputter-coated with gold before examination. The scanning electron microscope (SEM) S-4800 FESEM (Hitachi, Japan) was used to observe the grain endosperm structures.

### Measurement of physiological parameters

Wheat flag leaves were collected at different development stages (7, 14, 18, 21, and 28 DPA). RWC was determined by the standard method [80]. Leaf chlorophyll was extracted by 80% ethanol, and the contents of chlorophyll a and b were determined by spectrophotometry at 663 and 645 nm, respectively. Proline content was measured as described by Bates *et al.*[81]. WSC was determined according to previously described method [82].

### Sample preparation and iTRAQ labeling

Grain samples were ground into fine powder in liquid nitrogen using a mortar and pestle, and 500 mg samples were extracted for 2 h with 3 ml extraction buffer (50 mM Tris-HCl, pH 8.0, 0.1 M KCl, 5 mM EDTA, 30% sucrose) containing 1 mM PMSF. After centrifuging for 15 min at 13,000 rpm, supernatants were transferred to new tubes. Five-fold volumes of 10% cold TCA-acetone were added to the supernatants and stored for 2 h at -20°C, followed by centrifuging at 13,000 rpm for 15 min. The pellets were rinsed with 90% cold acetone, left at -20°C for 30 min, and then centrifuged at 13,000 rpm for 5 min. This step was repeated three times. After freeze drying, the final pellets were stored at -80°С or analyzed instantly.

Protein samples were incorporated into 500 μl STD buffer (4% SDS, 1 mM DTT, 150 mM Tris-HCl pH 8.0), and incubated in boiling water for 5 min, then subjected to ultra-sonication for 10 times (duration: 5 min; time interval: 5 min). After centrifuged at 13,000 g for 40 min, protein concentrations were determined by bicinchoninic acid (BCA) method. About 200 μg protein samples were diluted with 200 μl UA buffer (8 M urea, 150 mM Tris-HCl, pH 8.0), centrifuged at 14,000 g for 30 min, and then 200 μl UA buffer was added and centrifuged for another 30 min. After adding 100 μl UA buffer (50 mM iodoacetamide), the samples were incubated for 30 min in darkness, and then centrifuged for 20 min, and repeated twice. Then 100 μl DS buffer (50 mM triethylammoniumbicarbonate at pH 8.5) were added and centrifuged for 20 min. This step was repeated twice and then 40 μl trypsin solution (2 μg trypsin from Promega in 40 μl DS buffer) was added. The samples were incubated for about 16-18 h at 37°C. The resulting peptides were collected by centrifugation and the peptide content was tested by BCA method. The value of 1.1 at OD_280_ represented 1 μg/μl. About 100 μg peptides of each sample were labeled separately using the iTRAQ (Applied Biosystems, Foster City, CA, USA) standard protocol for the 4-plex kit (114, 115, 116 and 117 for 7, 14, 21 and 28 DPA, respectively, and sample labeled with 114 was used as the control).

### LC-MS/MS and data analysis

The mass spectrometry analysis was performed using Q-Exactive mass spectrometer (Thermo Finnigan), coupled with an Easy nLC system. In the HPLC procedure, mobile phase A (0.1% formic acid in water) and mobile phase B (0.1% formic acid in ACN) were selected. The sample was added through Thermo scientific EASY column (2 cm × 100 μm 5 μm-C18), and separated at a flow rate of 250 nl/min by Thermo scientific EASY column (75 μm × 100 mm 3 μm-C18). Peptide separation was performed with the following gradient comprised of 0–250 min from 0% to 60% B, 250–253 min from 35% to 100% B and 253–280 min of 100% B. A full mass spectrometry (MS) scan (300–1800 m/z) was acquired in the positive ion mode at a resolution of 70,000 (at 200 m/z), an AGC target value of 3–6, a maximum ion accumulation time of 10 ms, number of scan ranges of 1 and dynamic exclusion of 40.0 s. Information of peptides and peptide fragments m/z were collected as following: 10 fragment files were collected after every full scan (MS2 scan), higher collision energy dissociation (HCD) fragmentation, an isolation window of 2 m/z, full scan at a resolution of 17,500 (at 200 m/z), micro-scans of 1, a maximum ion accumulation time of 60 ms, normalized collision energy of 30 eV, and an under-fill ratio of 0.1%.

Three biological replicates were set for better coverage of the target proteome with reliable statistical consistency. For protein identification, the MS raw files were processed by Mascot 2.2 and Proteome Discoverer 1.3 (Thermo scientific, 2011). The acquired MS/MS spectra were automatically searched against the uniprot_pooideae_74417.fasta (Nov 16, 2012), and the total number of protein sequences used in this database was 74,417. A unique protein with at least two unique peptides, with a false discovery rate (FDR) <0.01, was qualified for further quantification data analysis. The parameters were set as: peptide mass tolerance of ±20 ppm, fragment mass tolerance of 0.1 Da, and number of allowed missed tryptic cleavage sites of 2. Protein quantification was based on the total intensity of the assigned peptides. The average of four labeled samples mixes was used as reference (REF), based on the weighted average of the intensity of report ions in each identified peptide. The final ratios of protein were normalized by the median average protein ratio for mixes of different labeled samples (7 d/REF, 14 d/REF, 21 d/REF, and 28 d/REF).

### RNA extraction and qRT-PCR analysis

Total RNA of wheat grains were extracted using TRIZOL Reagent (Invitrogen) according to the manufacturer’s instructions. Genomic DNA was removed by digesting each sample (20-50 μg of total RNA) with DNase I (Promega). According to the manufacturer’s instructions, 2 μl RNA sample was reversely transcribed to cDNA using PrimeScript® RT reagent Kit (DDR047A, Takara). Primer pairs for qRT-PCR analysis (Additional file 5: Table S3-D) were designed by the Primer3Plus program ( http://www.bioinformatics.nl/cgi-bin/primer3plus/primer3plus.cgi) and checked by blasting primer sequences in the NCBI database ( http://www.ncbi.nlm.nih.gov/tools/primerblast/index.cgi?LINK_LOC%20=%20BlastHome), and all primers were specifically consistent with the respective sequence of its targeted gene. ADP-ribosylation factor gene was used as reference for normalization, RT-PCR was performed in 20 μl volumes containing 9 μl 2.5 × RealMasterMix/20 × SYBR solution, 2 μl cDNA, 0.5 μl of each gene-specific primer and 8 μl ddH_2_O. PCR conditions were: 95°C for 3 min, 40 cycles of 20 s at 95°C, 55°C for 15 s and 72°C for 20 s, a melt curve of 65°C to 95°C. Reactions were conducted on a CFX96 Real-time PCR Detection System (Bio-Rad). All data were analyzed with CFX Manager Software (Bio-Rad).

### Phosphopeptide enrichment, identification and phosphorylation residue localization

The protein mixtures from developing grains at 28 DPA was directly reduced with dithiothreitol (DTT), alkylated with iodoacetamide, and subsequently digested with endoproteinase Lys-C and trypsin, as described previously [29]. The enrichment and identification for the phosphopeptide procedure were performed as the recently reported method [29]. Three biological replicates were performed independently for the phosphopeptide identification using LC-MS/MS.

The raw files were processed using MaxQuant (version 1.2.2.5) [83], and searched against the uniprot_pooideae_74417.fasta (Nov 16, 2012), and the total number of protein sequences used in this database was 74,417. Up to two missing cleavage points were allowed. The precursor ion mass tolerance was 7 ppm, and the fragment ion mass tolerance was 0.5 Da for the MS/MS spectra. The false discovery rate (FDR) was set to <1.0% for the identification of both peptides and proteins. The minimum peptide length was set to 6.

Phosphorylation residue localization was evaluated based on the PTM scores, which assign the probabilities for each of the possible residues according to their residue-determining ions. In this study, MaxQuant (version 1.2.2.5) was used to calculate the PTM scores and PTM localization probabilities. Potential phosphorylation residues were then grouped into three categories depending on their PTM localization probabilities [29, 84], namely, class I (localization probability, P ≥0.75), class II (0.75 > P ≥0.5), and class III (P <0.5). A false discovery rate (FDR) of 1.0% was used for the identification of phosphorylation residues. Spectra without residue-determining ions led to the identification of phosphopeptides with undetermined residues.

### Bioinformatics

Proteins were examined using AgriGO [85] for gene ontology (GO) annotation and enrichment analysis. The Search Tool for the Retrieval of Interacting Genes/Proteins (STRING) database of physical and functional interactions was used to analyze the protein-protein interaction (PPI) of all the proteins identified in the current study. The sequences were blasted in the EggNog databases ( http://eggnog.embl.de/version_4.0.beta/) to obtain the protein KOG numbers. The dataset containing all of the KOG numbers was then used to conduct PPI analysis by STRING, and some important networks were displayed using Cytoscape (version 3.0.2) software. The Phyre2 (namely, the protein homology/analogy recognition engine v 2.0) ( http://www.sbg.bio.ic.ac.uk/phyre2/html/page.cgi?id=index) was used to predict the 3D structure of the phosphoproteins. The phosphorylated residues were displayed using Swiss-PdbViewer (SPDBV) version 4.1 software ( http://spdbv.vital-it.ch/).

## Electronic supplementary material

Additional file 1: Figure S1: The physiological changes of flag leaves from Yanyou 361 during different grain development stages. A: Relative water content; B: Chlorophyll content; C: WSC content; D: Proline content. (JPEG 2 MB)

Additional file 2: Table S1: Complete list of normalized expression values obtained from three experimental series based on three biological replicates. (XLSX 569 KB)

Additional file 3: Table S2: Complete list of proteins identified by iTRAQ. (XLSX 8 MB)

Additional file 4: Figure S2: Pearson correlation between three biology replicates of iTRAQ test during different development stages. a. 7 DPA; b. 14 DPA; c. 21 DPA; d. 28 DPA. (JPEG 2 MB)

Additional file 5: Table S3-A: The detail information of 421 DEPs identified by iTRAQ in wheat grain development stages; **Table S3-B:** All KOG of DEPs; **Table S3-C:** The information of the representative phosphorylated proteins; **Table S3-D:** Primers of DEPs encoding genes for qRT-PCR. Table S3-E: KOG of DEPs interacted with chaperones; **Table S3-F**: Primers of DEPs encoding genes for qRT-PCR. (XLS 266 KB)

Additional file 6: Figure S3: Representative MS spectra of identified peptides from developing grains of Yanyou 361. (PDF 104 KB)

Additional file 7: Figure S4: GO functional enrichment of DEPs of different expression pattern subsets (**A:** up-regulation subset; **B:** down-regulation subset; **C:** up-to-down-regulation subset). The statistical significance of the enrichment analysis is represented by a scale of red tones whose intensity is proportional to the degree of significance starting from FDR <0.05. (PDF 461 KB)

Additional file 8: Figure S5: Melting curves and standard curves of qRT-PCR analysis. (JPEG 4 MB)

Additional file 9: Figure S6: Representative MS spectra of phosphopeptides and Uniprot ID of the corresponding proteins. (PDF 4 MB)

Additional file 10: Figure S7: A complex PPI network with 79 nodes analyzed by STRING. Interactions of the DEPs are extracted by searching the STRING database with a confidence cutoff of 0.900. The interaction network is reconstructed by using the Cytoscape software. (JPEG 5 MB)

Additional file 11: Figure S8: SDS-PAGE identification of HMW-GS (1, 17 + 18, 5 + 10) from Yanyou 361. Chinese Spring (CS) (N, 7 + 8, 2 + 12) was used as the standard. (JPEG 266 KB)

## Abbreviations

2-DE: Two-dimensional electrophoresis
2D-DIGE: Two-dimensional differential gel electrophoresis
αAI: alpha-amylase inhibitor
ADP-Glu: ADP-glucose
AGPase: ADP-glucose pyrophosophorylase
BT1: ADP-glucose brittle-1 transporter
CTI: Chymotrypsin inhibitor WCI
DBE: De-branching enzyme
DEPs: Differentially expressed proteins
DPA: Day post anthesis
EmP: Early-methionine-labelled polypeptide
G1P: Glucose-1-phosphate
GBSS: Granule bind starch synthase
GS: Glutamine synthetase
GSP: Grain softness protein
HMW-GS: High molecular weight glutenin subunit
Hsp: Heat shock protein
iTRAQ: Isobaric tag for relative and absolute quantitation
KOG: Eukaryotic Orthologous Group
LEA: Late embryogenesis abundant
LMW-GS: Low molecular weight glutenin subunit
PDI: Pdisulfide isomerase
PGK: Phosphoglycerate kinase
PPI: Protein-protein interaction
PRs: Pathogenesis-related proteins
PTMs: Post-translational modifications
qRT-PCR: Quantitative real-time polymerase chain reaction
ROS: Reactive oxygen species
RuBisCO: Ribulose bisphosphate carboxylase
RWC: Relative water content
SBE: Starch branching enzymes
SEM: Scanning electronic microscope
SS: Starch synthase
SuSy: Sucrose synthases
WSC: Water soluble content
XI: Xylanase inhibitor protein.
