# Supplementary figures and images for: iTRAQ-based quantitative proteome and phosphoprotein characterization reveals the central metabolism changes involved in wheat grain development
Source: BMC Genomics. 2014 Nov 27;15(1):1029. doi: 10.1186/1471-2164-15-1029 (PMC4301063; doi:10.1186/1471-2164-15-1029)

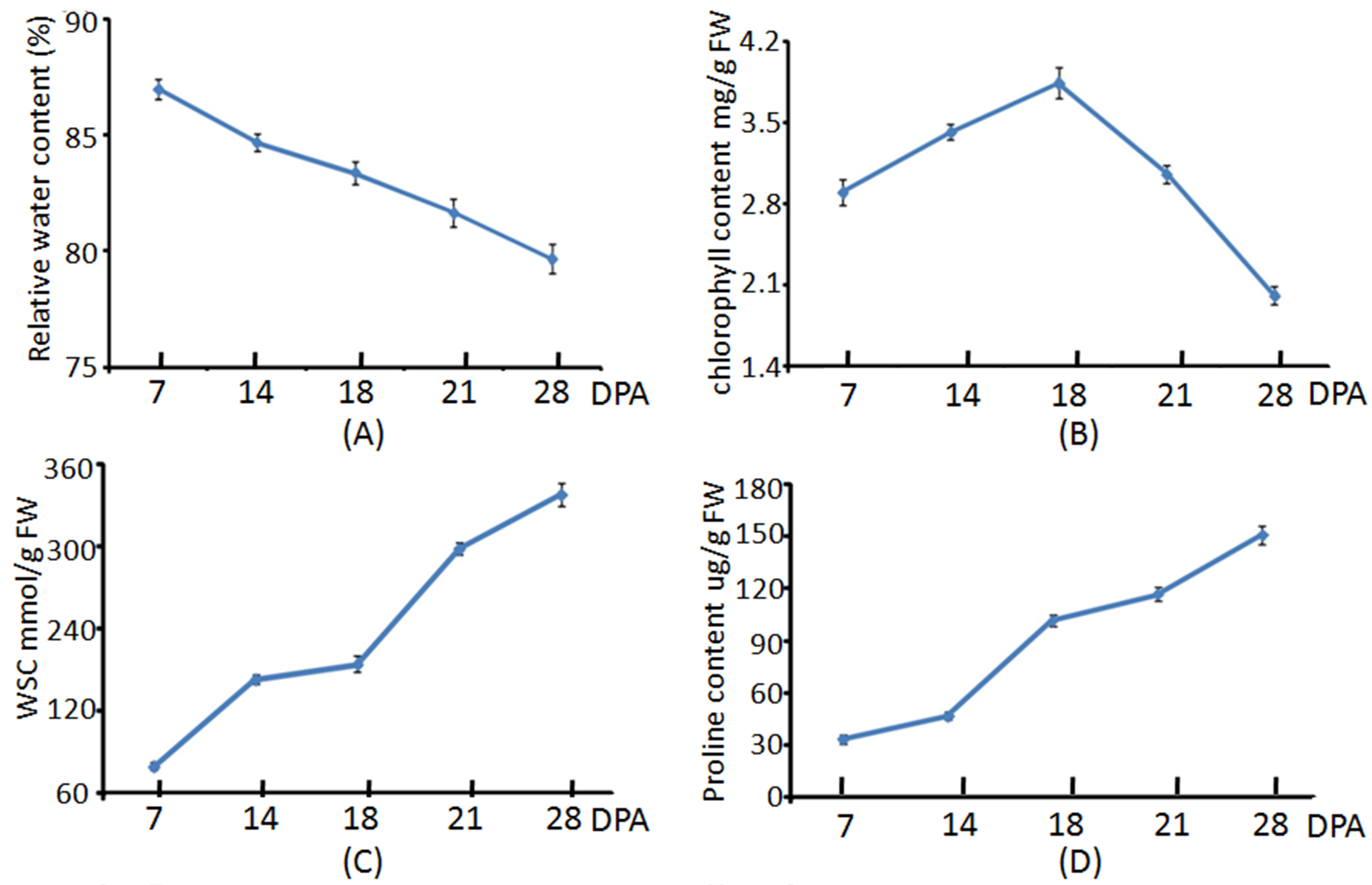

Supplement: Supplementary file 1 — Additional file 1: Figure S1: The physiological changes of flag leaves from Yanyou 361 during different grain development stages. A: Relative water content; B: Chlorophyll content; C: WSC content; D: Proline content. (JPEG 2 MB) [file 12864_2014_6842_MOESM1_ESM.jpeg]

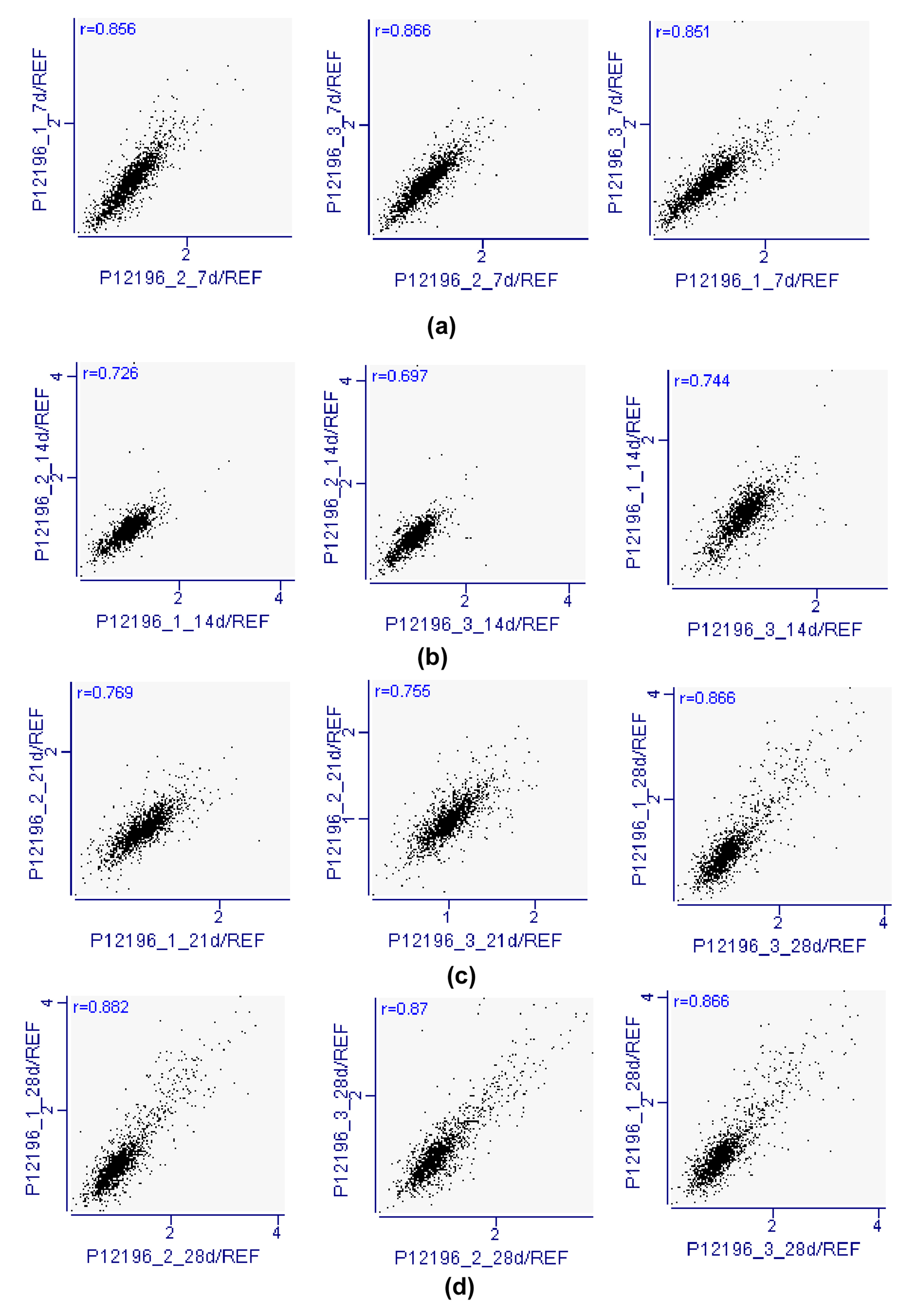

Supplement: Supplementary file 4 — Additional file 4: Figure S2: Pearson correlation between three biology replicates of iTRAQ test during different development stages. a. 7 DPA; b. 14 DPA; c. 21 DPA; d. 28 DPA. (JPEG 2 MB) [file 12864_2014_6842_MOESM4_ESM.jpeg]

Figure S4-A

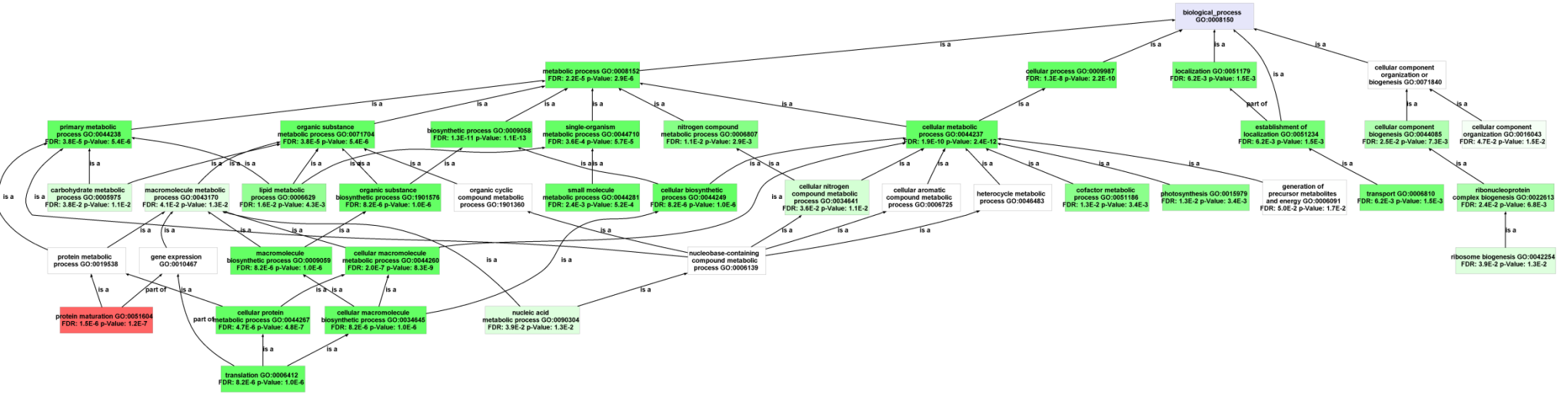

Enriched Graph

### Figure S4-B

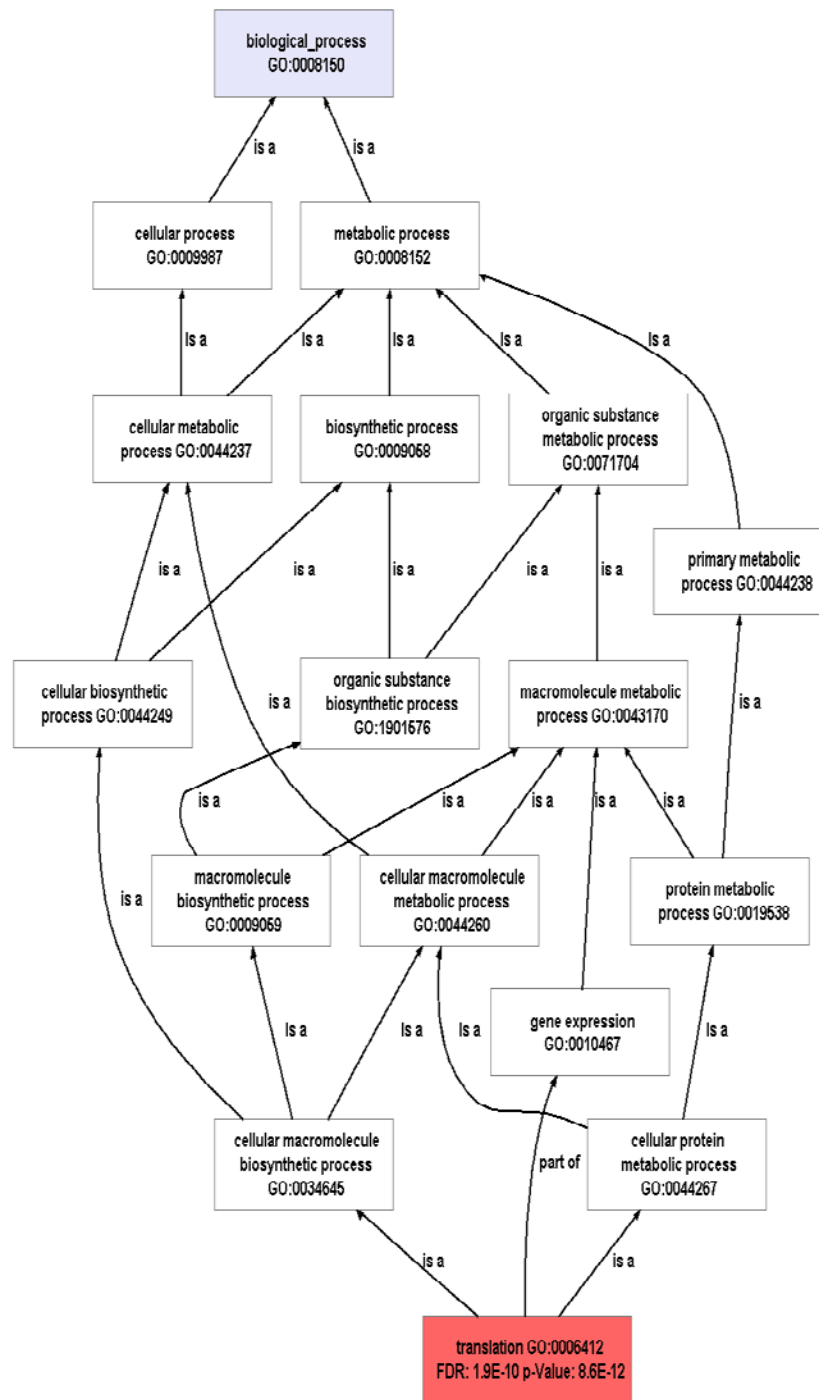

### Enriched Graph

Figure S4-C

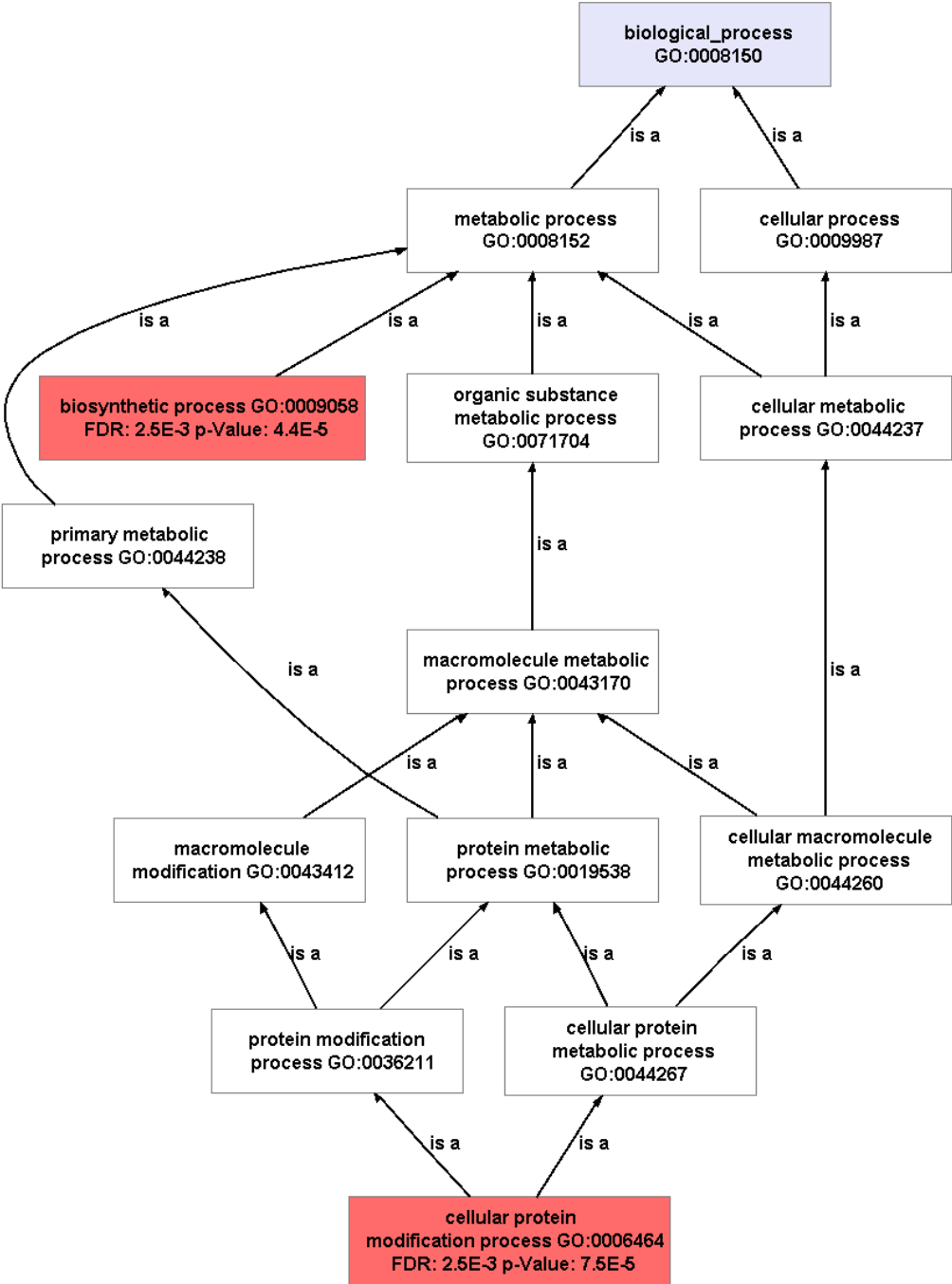

Enriched Graph

Supplement: Supplementary file 7 — Additional file 7: Figure S4: GO functional enrichment of DEPs of different expression pattern subsets (A: up-regulation subset; B: down-regulation subset; C: up-to-down-regulation subset). The statistical significance of the enrichment analysis is represented by a scale of red tones whose intensity is proportional to the degree of significance starting from FDR <0.05. (PDF 461 KB) [file 12864_2014_6842_MOESM7_ESM.pdf]

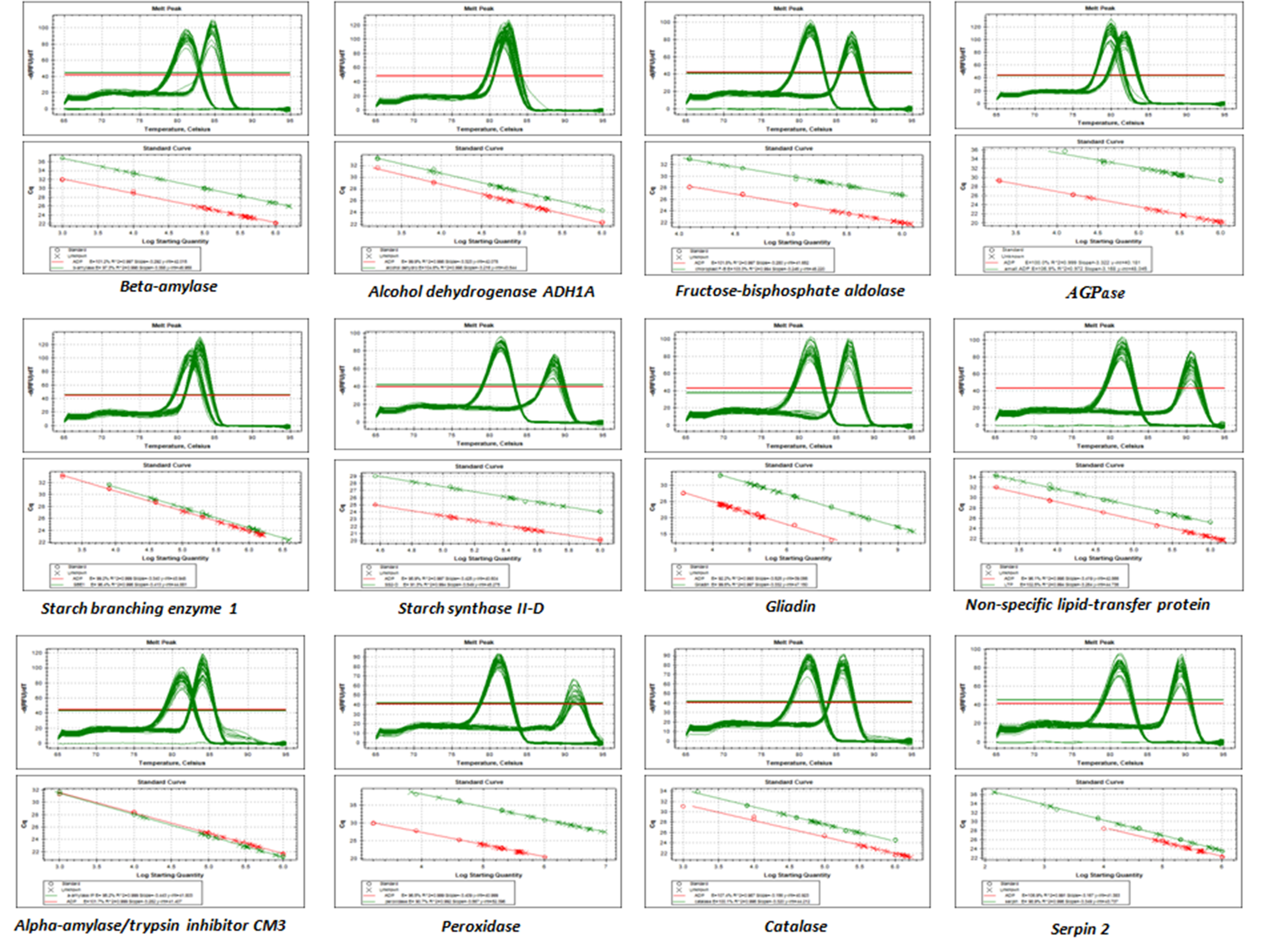

Supplement: Supplementary file 8 — Additional file 8: Figure S5: Melting curves and standard curves of qRT-PCR analysis. (JPEG 4 MB) [file 12864_2014_6842_MOESM8_ESM.jpeg]

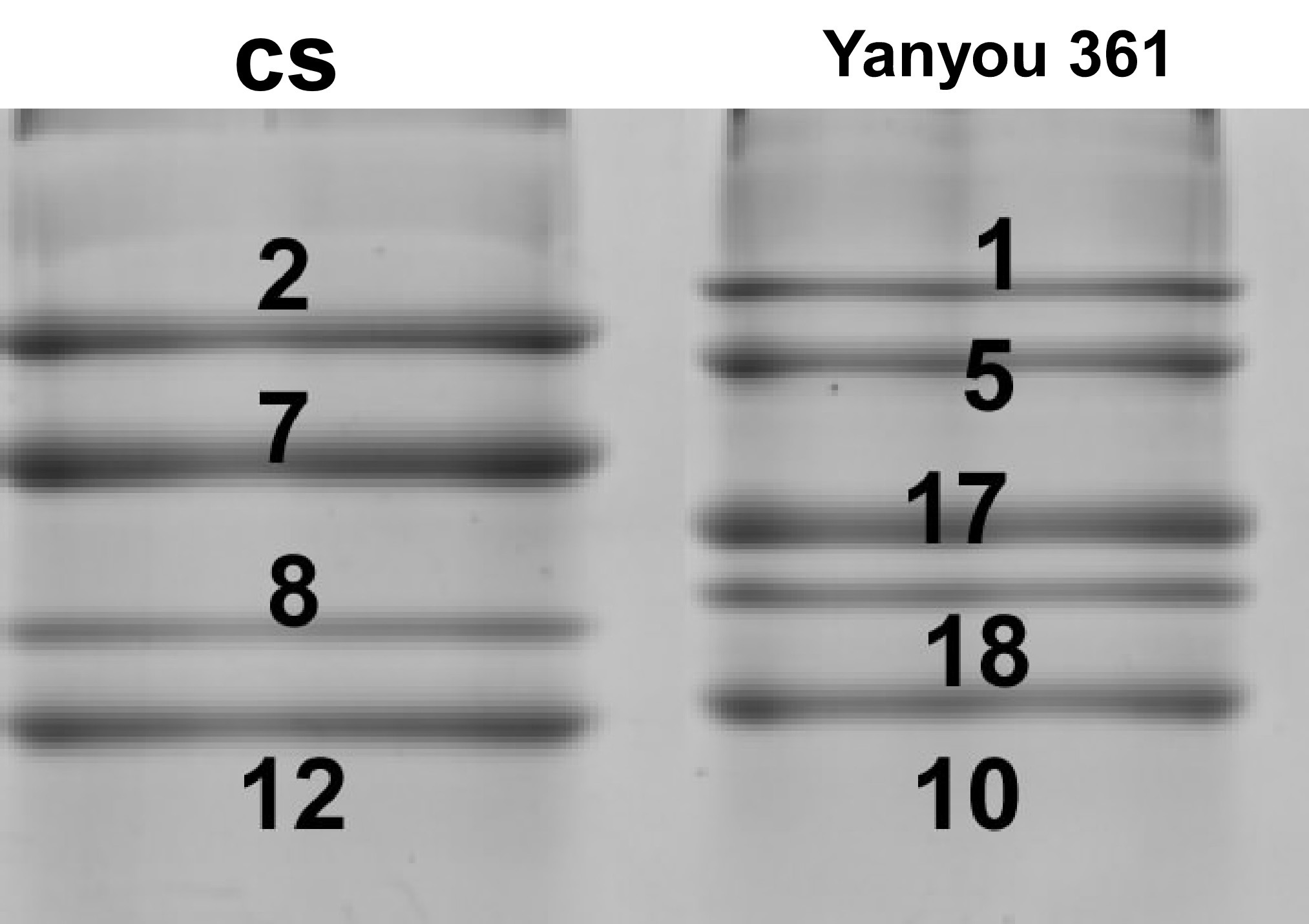

Supplement: Supplementary file 11 — Additional file 11: Figure S8: SDS-PAGE identification of HMW-GS (1, 17 + 18, 5 + 10) from Yanyou 361. Chinese Spring (CS) (N, 7 + 8, 2 + 12) was used as the standard. (JPEG 266 KB) [file 12864_2014_6842_MOESM11_ESM.jpeg]
