# Supplementary material for: iTRAQ-based quantitative proteome and phosphoprotein characterization reveals the central metabolism changes involved in wheat grain development
Source: BMC Genomics. 2014 Nov 27;15(1):1029. doi: 10.1186/1471-2164-15-1029 (PMC4301063; doi:10.1186/1471-2164-15-1029)
Supplement: Supplementary file 6 — Additional file 6: Figure S3: Representative MS spectra of identified peptides from developing grains of Yanyou 361. (PDF 104 KB) [file 12864_2014_6842_MOESM6_ESM.pdf]

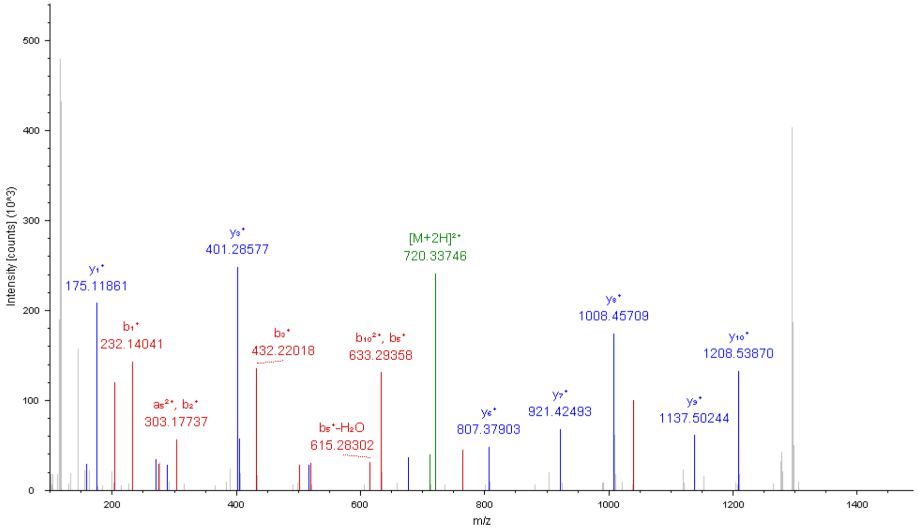

1. Fructose-bisphosphate aldolase

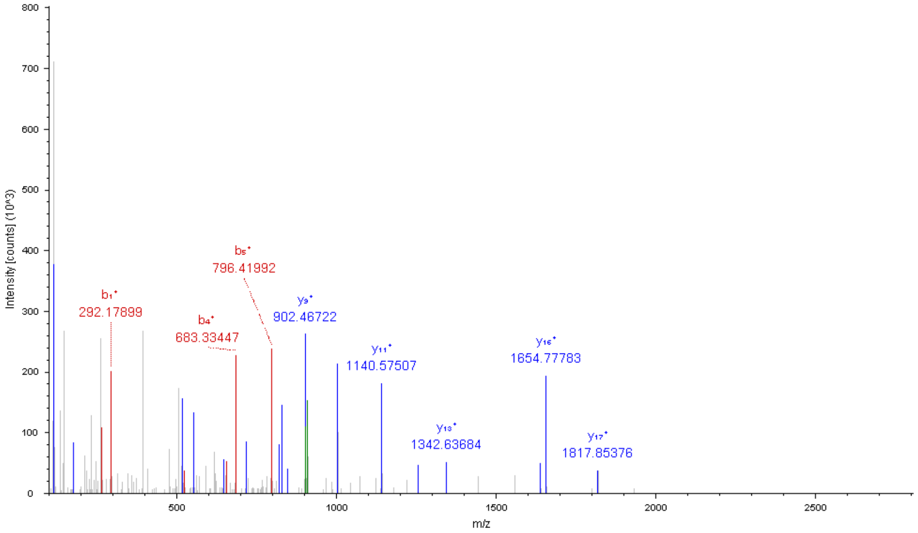

2. Lipoxygenase

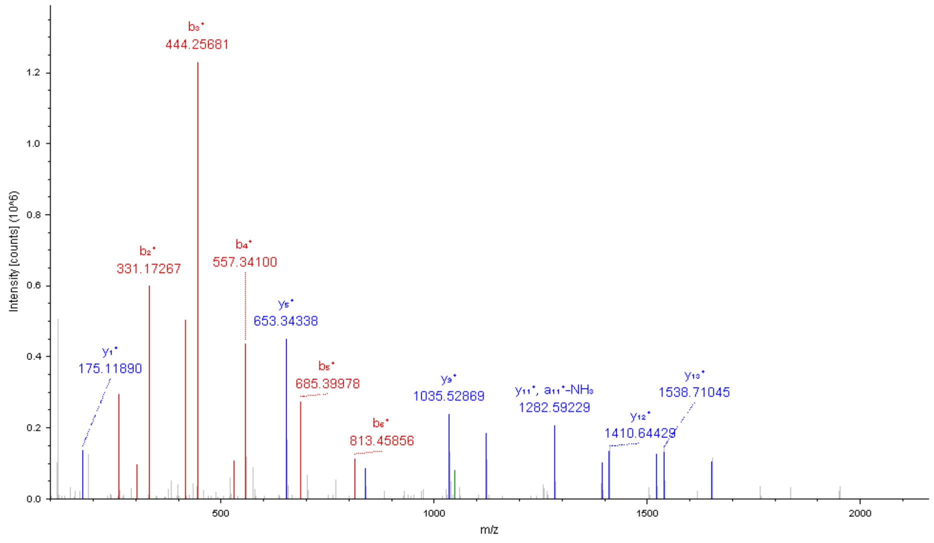

3. Gliadin/avenin-like seed protein

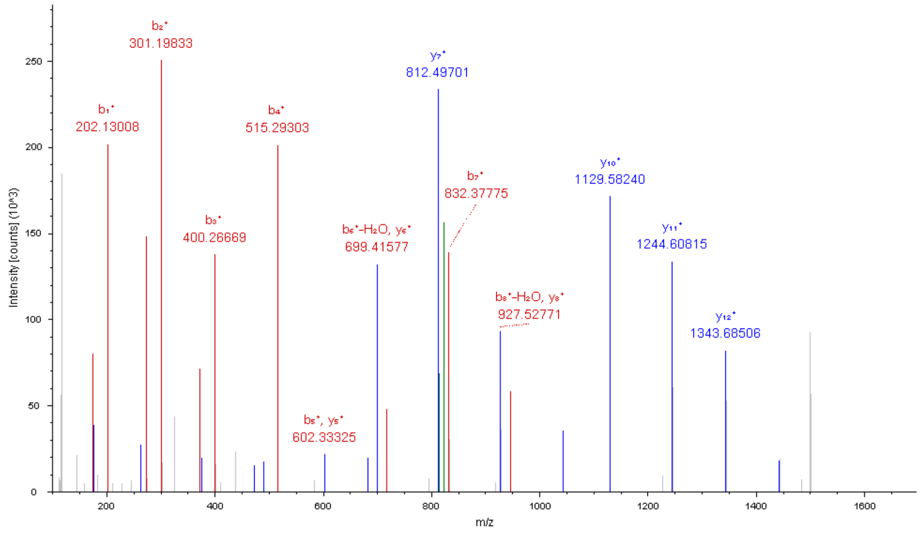

4. Heat shock protein 90

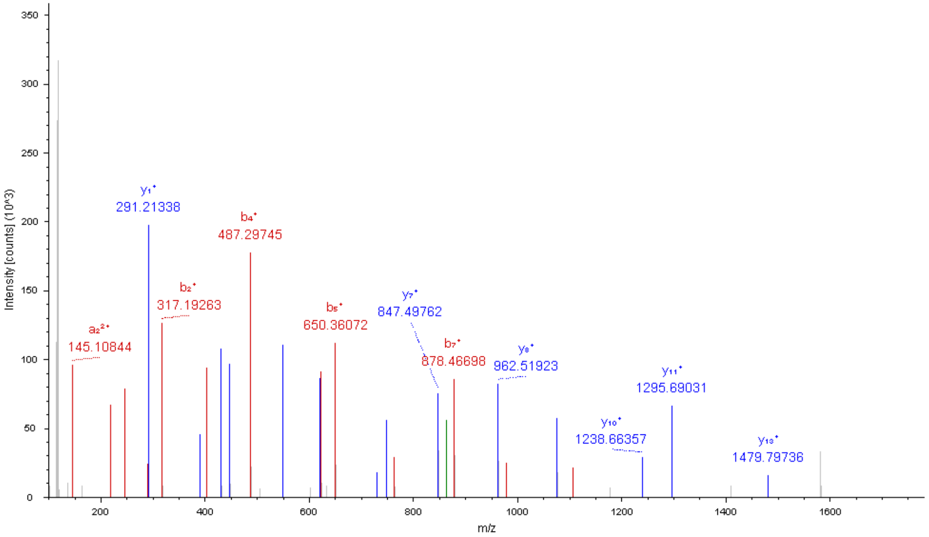

5. Starch branching enzyme 1

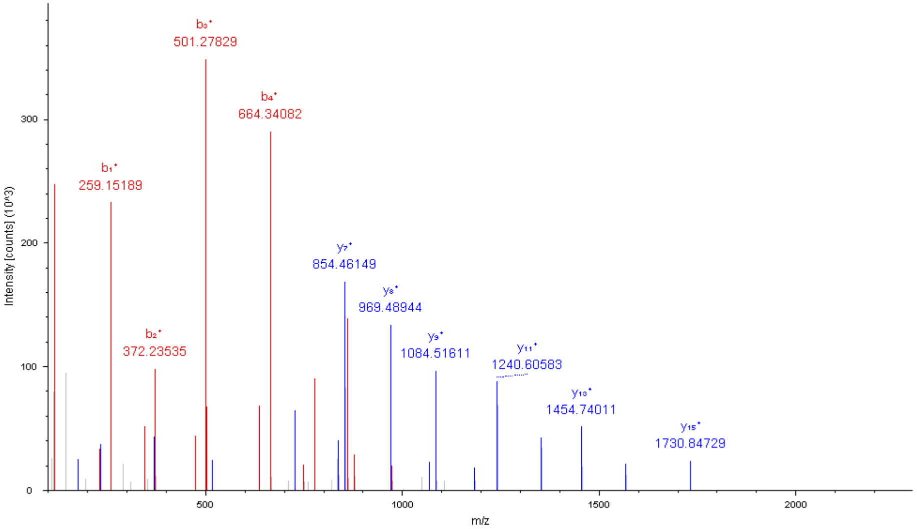

6. Beta-amylase

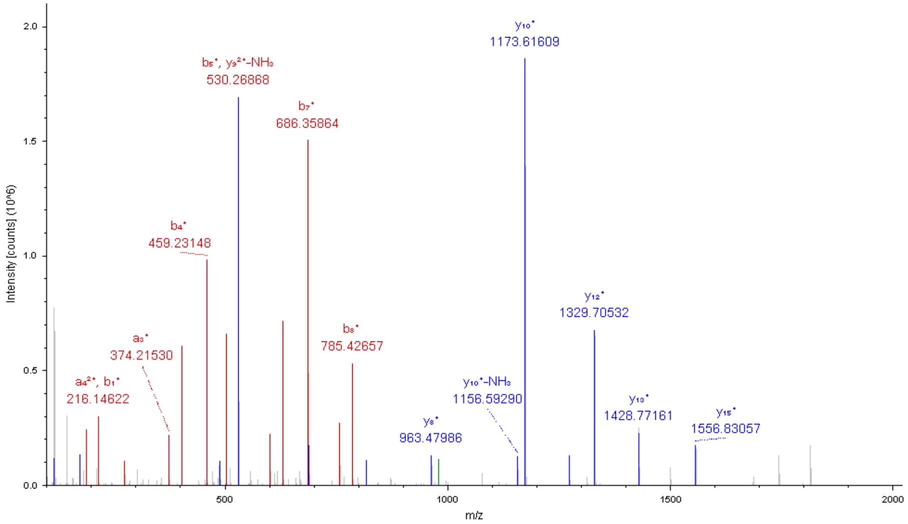

7. Globulin 1

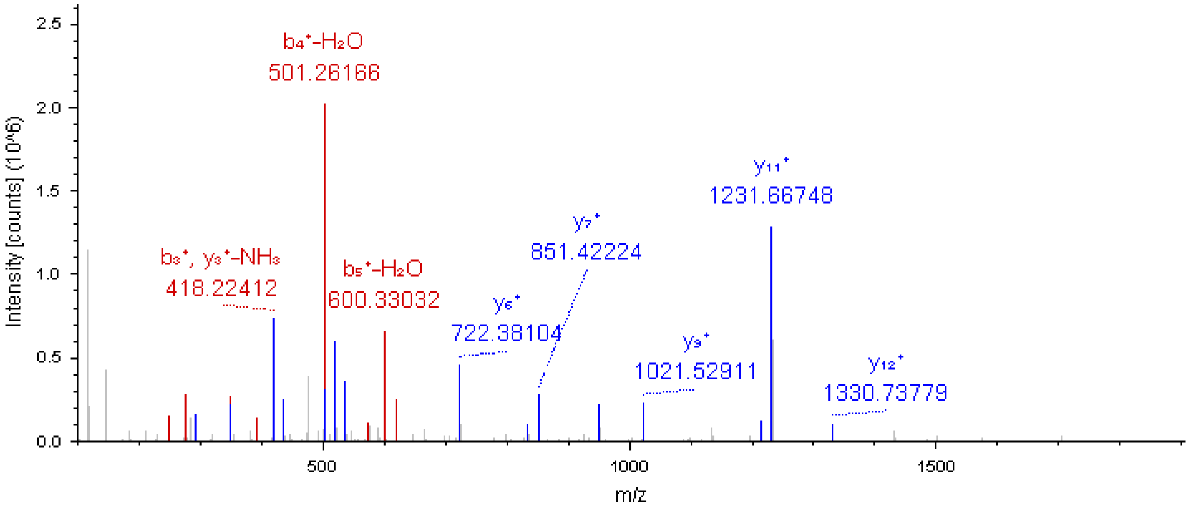

8. Sucrose synthase

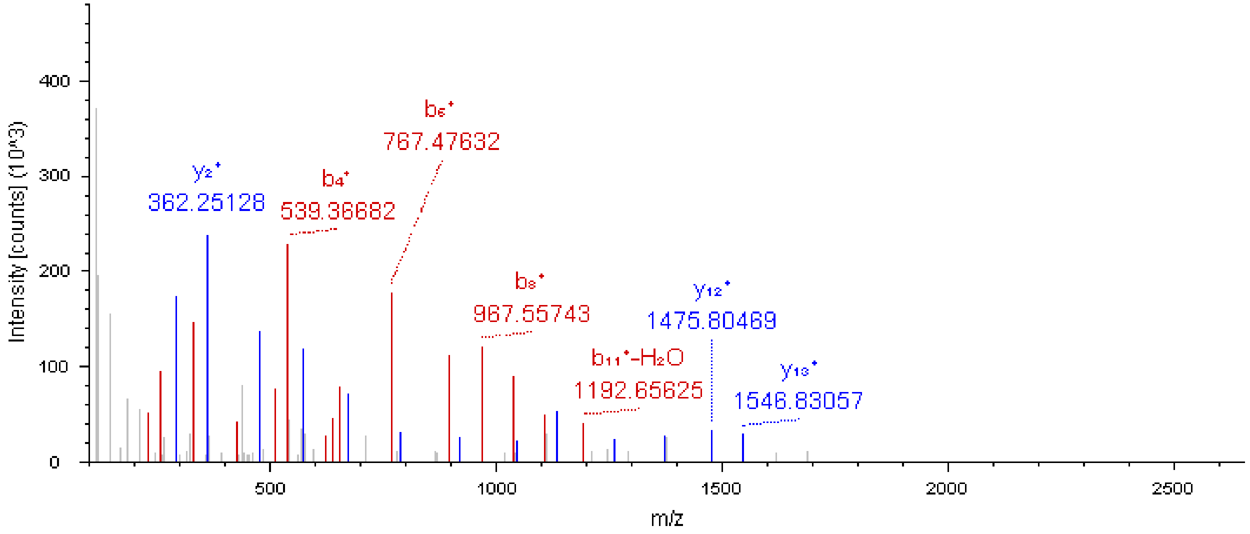

9. Protein disulfide isomerase

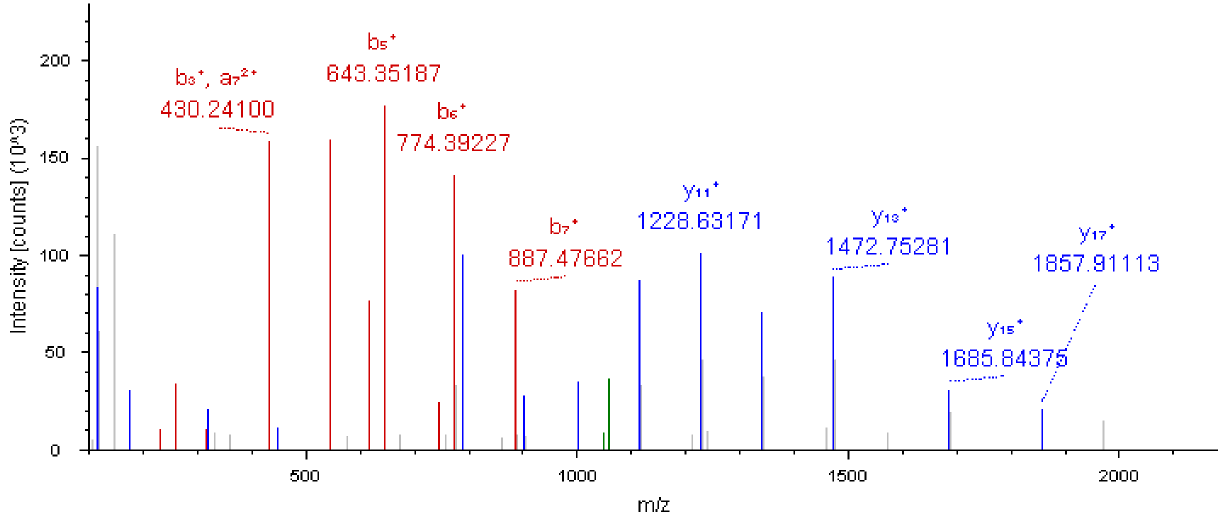

10. Glucose-1-phosphate adenylyltransferase

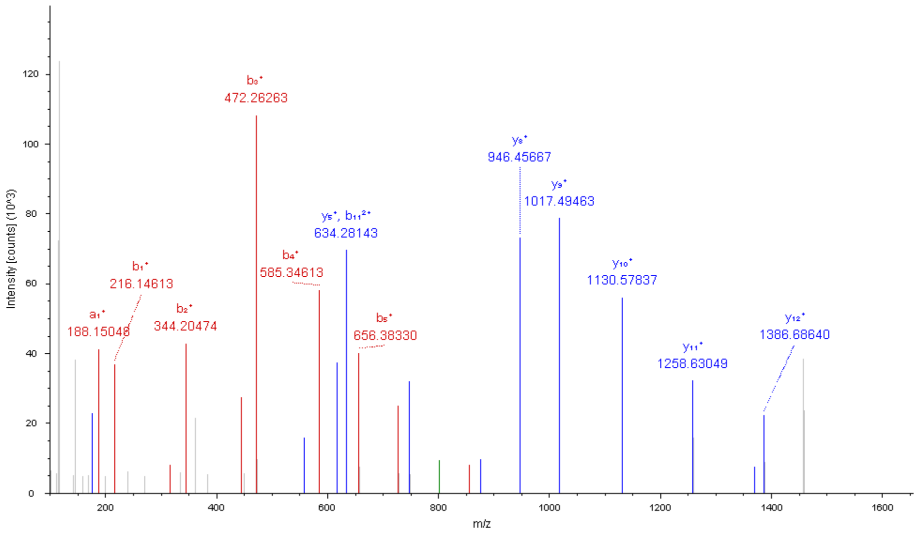

11 High-molecular-weight glutenin subunit Bx17

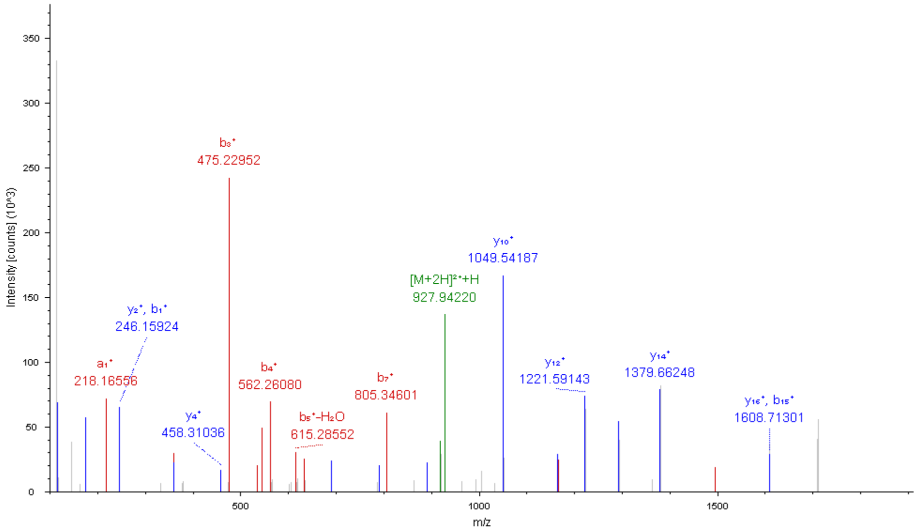

12. RuBisCO large subunit-binding protein subunit alpha
