# Supplementary material for: iTRAQ-based quantitative proteome and phosphoprotein characterization reveals the central metabolism changes involved in wheat grain development
Source: BMC Genomics. 2014 Nov 27;15(1):1029. doi: 10.1186/1471-2164-15-1029 (PMC4301063; doi:10.1186/1471-2164-15-1029)
Supplement: Supplementary file 9 — Additional file 9: Figure S6: Representative MS spectra of phosphopeptides and Uniprot ID of the corresponding proteins. (PDF 4 MB) [file 12864_2014_6842_MOESM9_ESM.pdf]

Additional file 7: Figure S4: Representative representative MS spectras for phosphopeptides for identified peptides and Uniprot ID of corresponding proteins.

# B6VCM0/ P12299

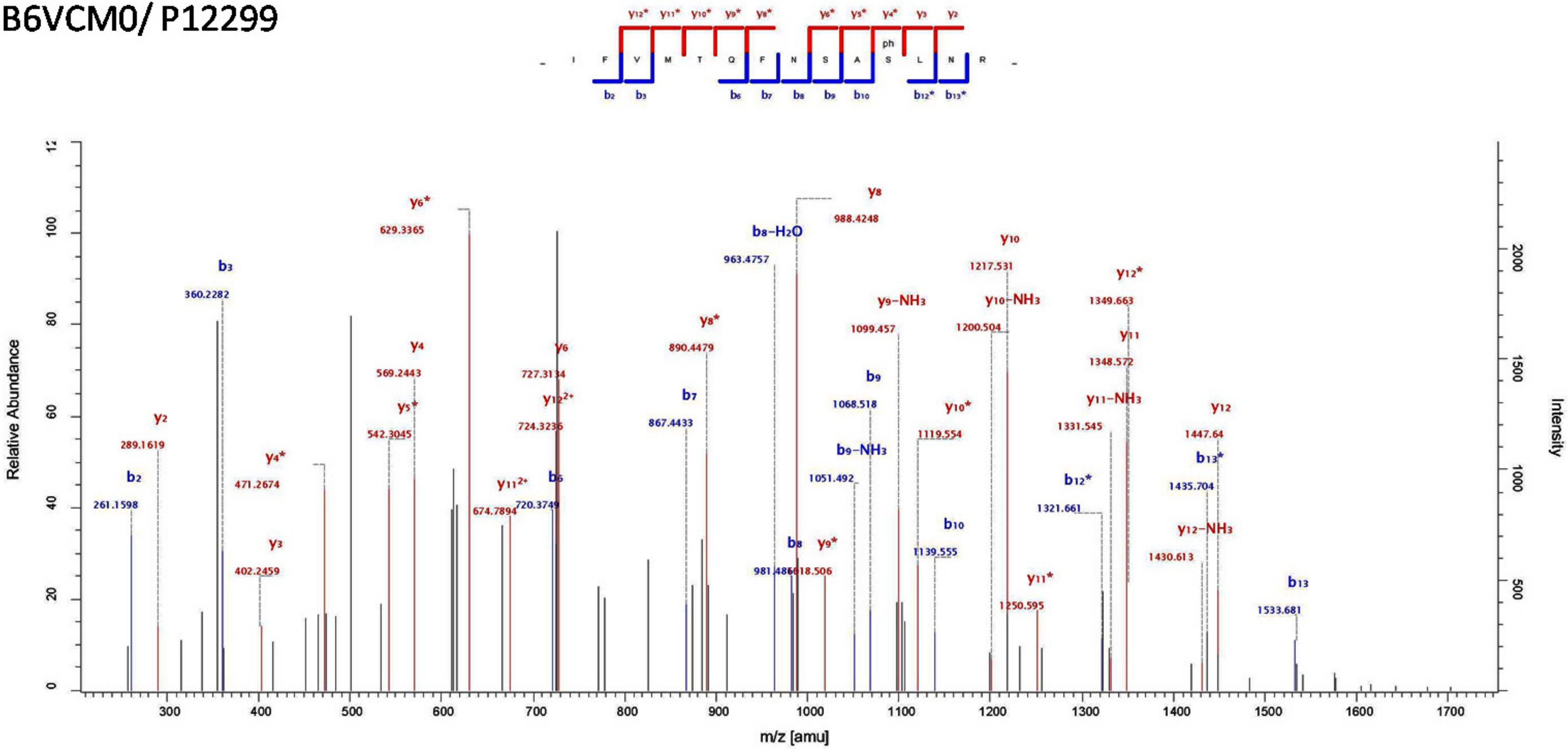

P12299

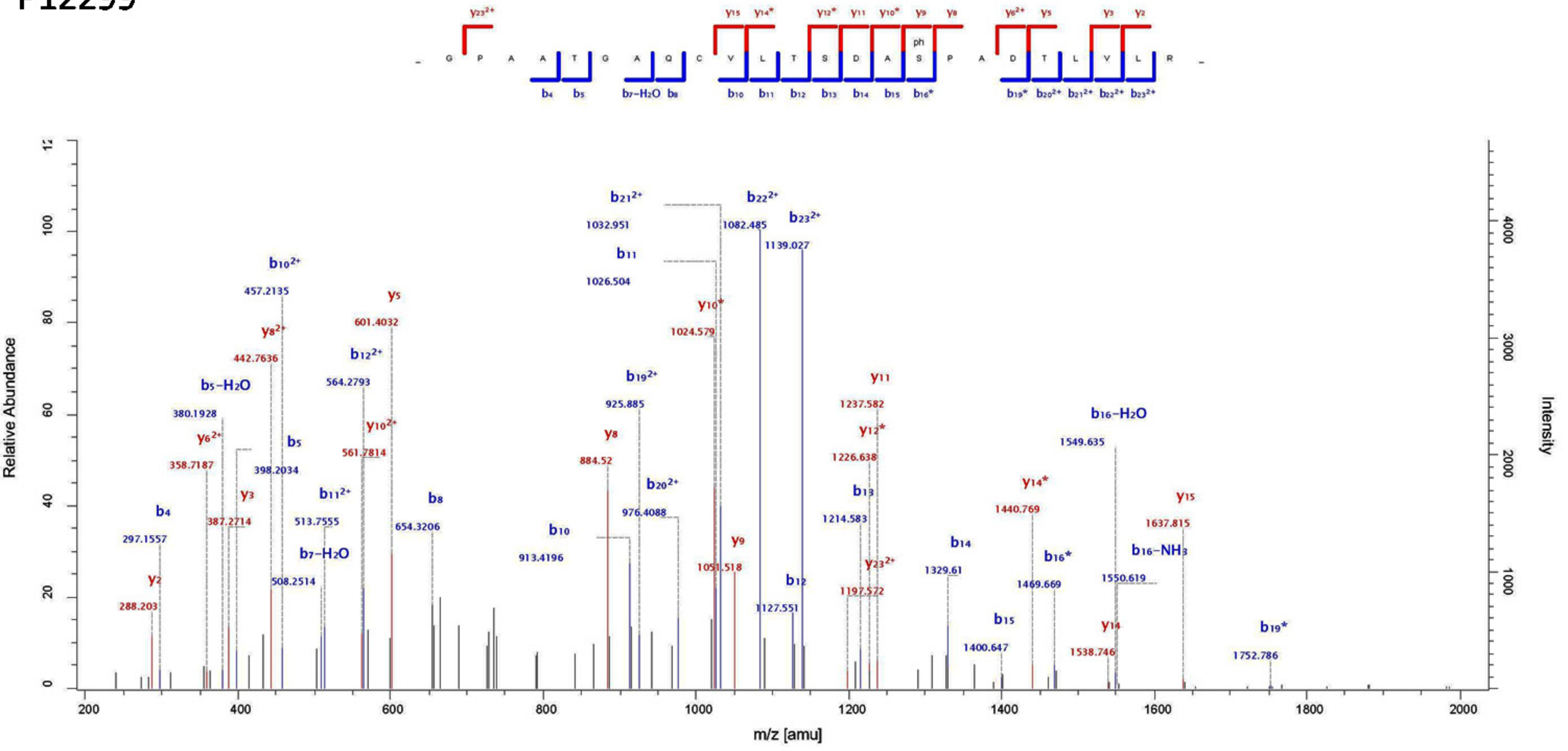

Relative Abundance
Intensity
m/z [amu]
b21 2+ 1032.951
b22 2+ 1082.485
b23 2+ 1139.027
b11 1026.504
y10* 1024.579
b10 2+ 457.2135
y5 601.4032
y8 2+ 442.7636
b12 2+ 564.2793
b5-H2O 380.1928
y6 2+ 358.7187
b5 398.2034
y10 2+ 561.7814
b19 2+ 925.885
y8 884.52
b20 2+ 976.4088
y11 1237.582
y12* 1226.638
b16-H2O 1549.635
y3 387.2714
b11 2+ 513.7555
b8 654.3206
b4 297.1557
b10 913.4196
b7-H2O 508.2514
y2 288.203
y9 1051.518
b13 1214.583
y14* 1440.769
y15 1637.815
b14 1329.61
b16* 1469.669
b16-NH3 1550.619
y23 2+ 1197.572
b12 1127.551
b15 1400.647
y14 1538.746
b19* 1752.786

# Q9M4Z1/ Q5XXD1

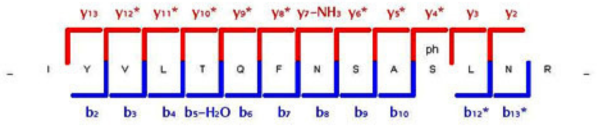


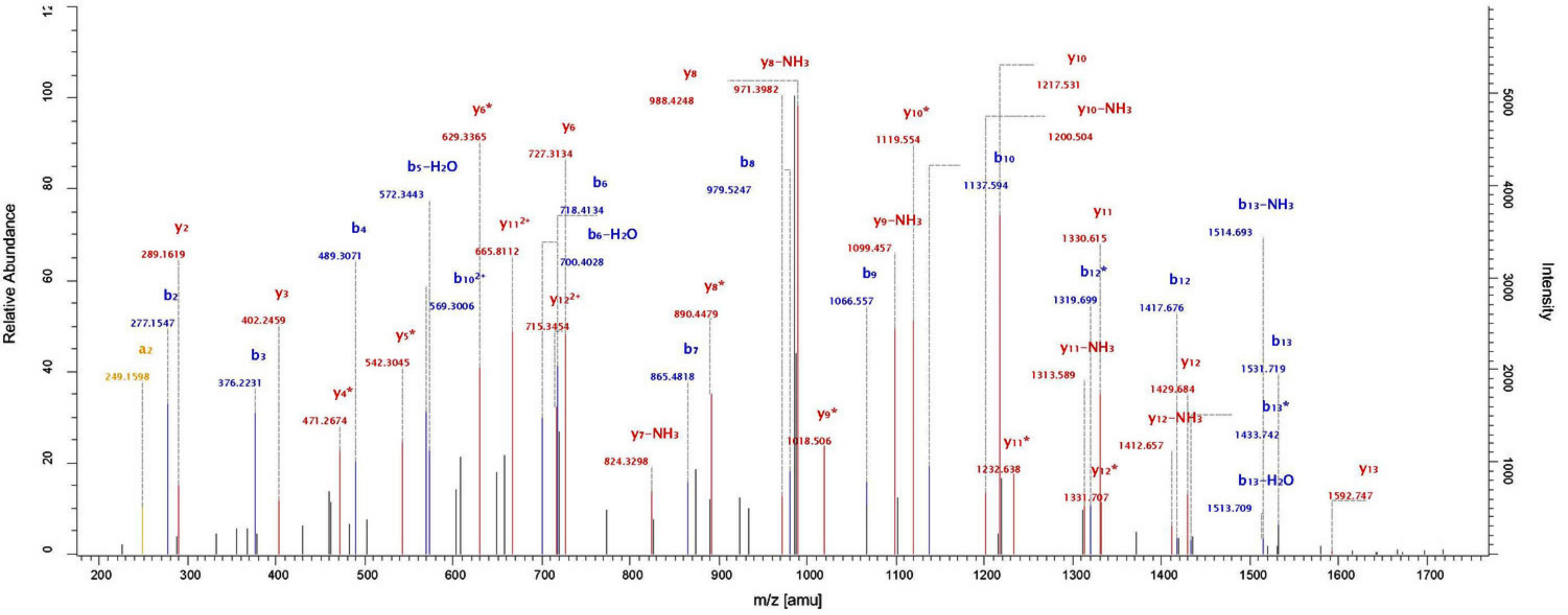

# P93594

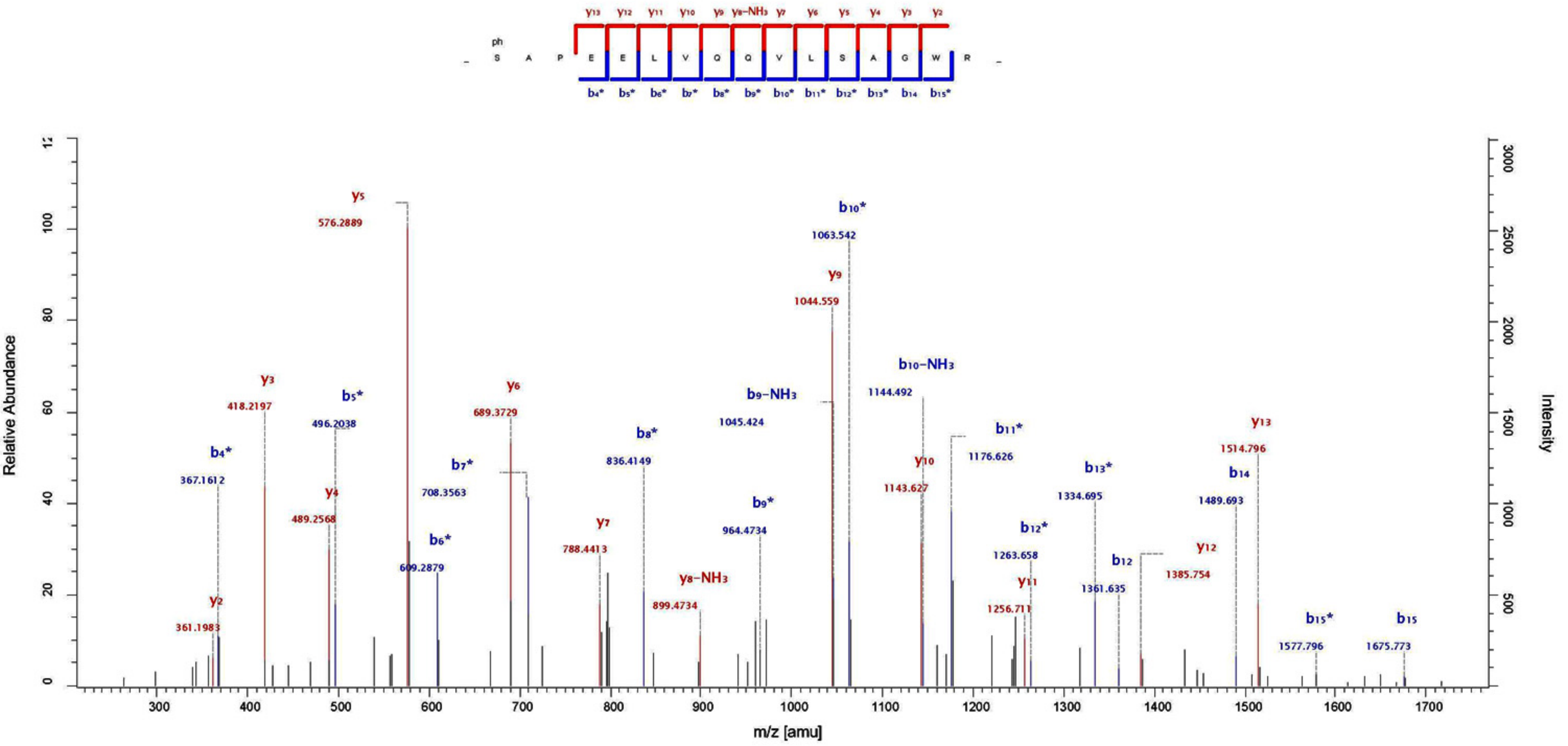

y13 y12 y11 y10 y9 y8-NH3 y7 y6 y5 y4 y3 y2
ph
_ S A P E E L V Q Q V L S A G W R _
b4* b5* b6* b7* b8* b9* b10* b11* b12* b13* b14 b15*
y5 576.2889
b10* 1063.542
y9 1044.559
b10-NH3 1144.492
b9-NH3 1045.424
y3 418.2197
b5* 496.2038
y6 689.3729
b11* 1176.626
y13 1514.796
b4* 367.1612
b8* 836.4149
y10 1143.627
b13* 1334.695
b7* 708.3563
b14 1489.693
y4 489.2568
b9* 964.4734
b6* 609.2879
y7 788.4413
b12* 1263.658
y12 1385.754
b12 1361.635
y8-NH3 899.4734
y11 1256.711
y2 361.1983
b15* 1577.796
b15 1675.773
Relative Abundance
Intensity
m/z [amu]

# Q43223

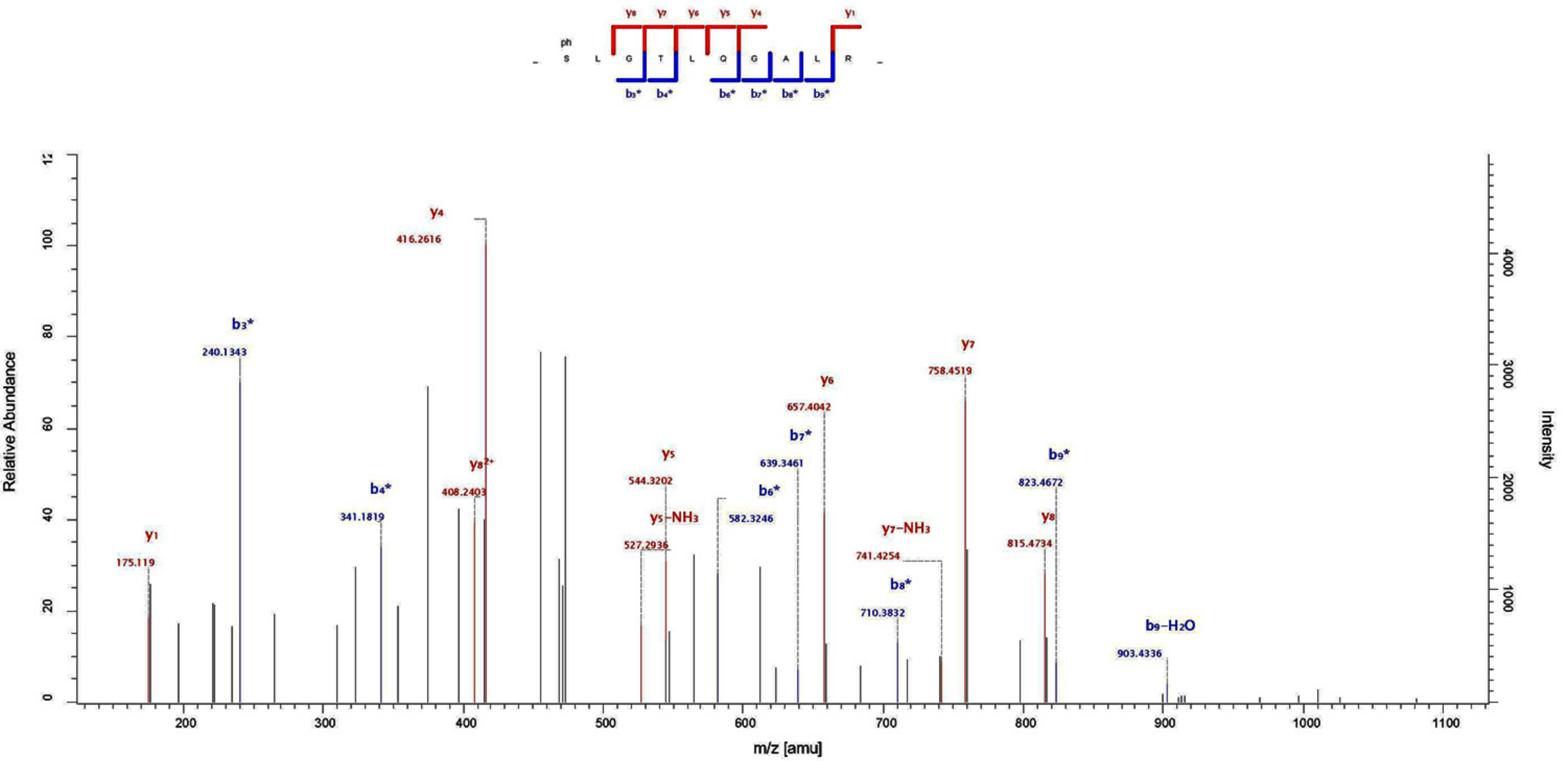

ph
S L G T L Q G A L R
y8 y7 y6 y5 y4 y1
b3* b4* b6* b7* b8* b9*
y4
416.2616
b3*
240.1343
y7
758.4519
y6
657.4042
b7*
639.3461
y5
544.3202
y8 2+
408.2403
b4*
341.1819
b6*
582.3246
b9*
823.4672
y1
175.119
y5-NH3
527.2936
y7-NH3
741.4254
y8
815.4734
b8*
710.3832
b9-H2O
903.4336
Relative Abundance
Intensity
m/z [amu]

# A9U8G4

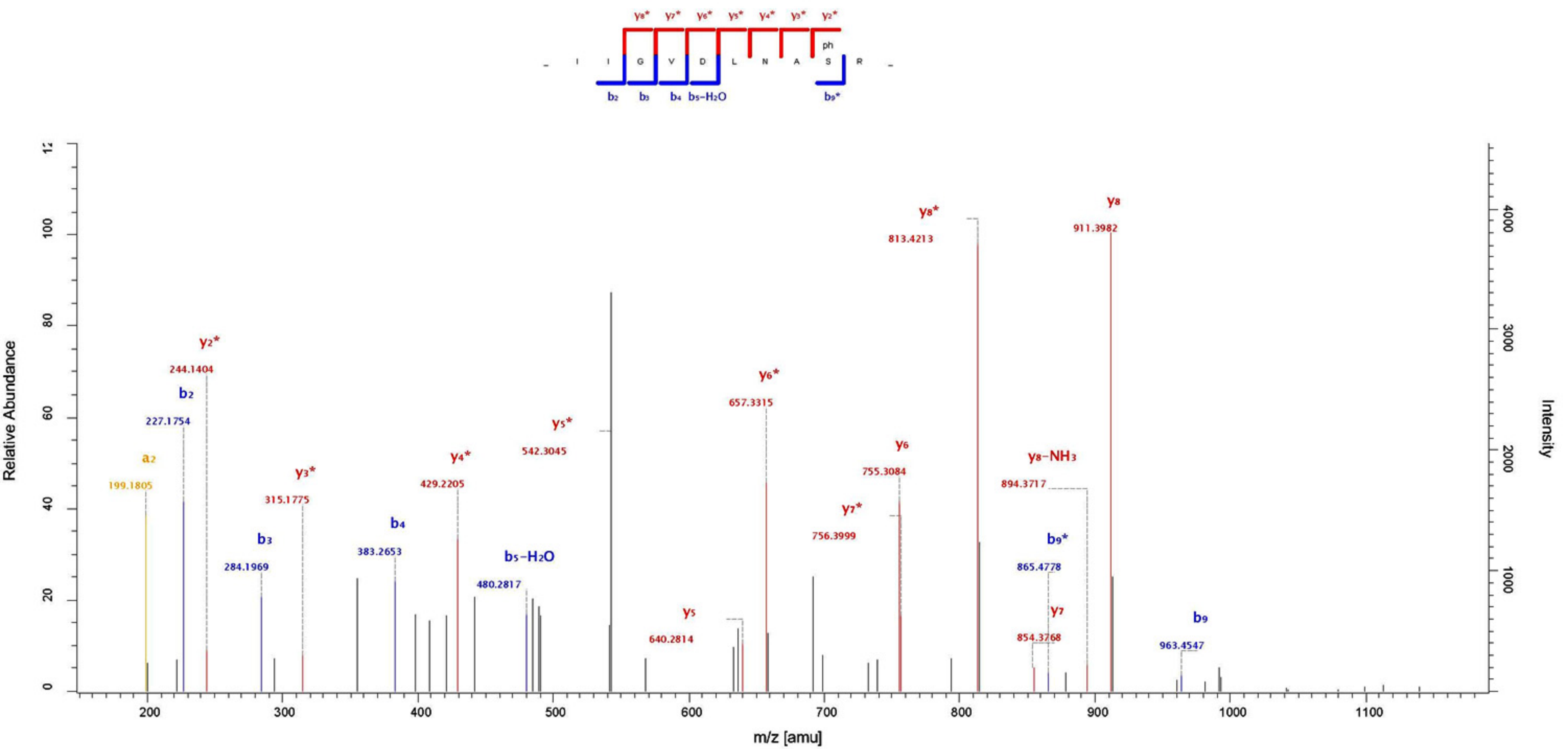

y8* y7* y6* y5* y4* y3* y2*
ph
I I G V D L N A S R
b2 b3 b4 b5-H2O b9*
Relative Abundance
Intensity
m/z [amu]
a2 199.1805
b2 227.1754
y2* 244.1404
b3 284.1969
y3* 315.1775
b4 383.2653
y4* 429.2205
b5-H2O 480.2817
y5* 542.3045
y5 640.2814
y6* 657.3315
y6 755.3084
y7* 756.3999
y8* 813.4213
y7 854.3768
b9* 865.4778
y8-NH3 894.3717
y8 911.3982
b9 963.4547

# Q6UZD6

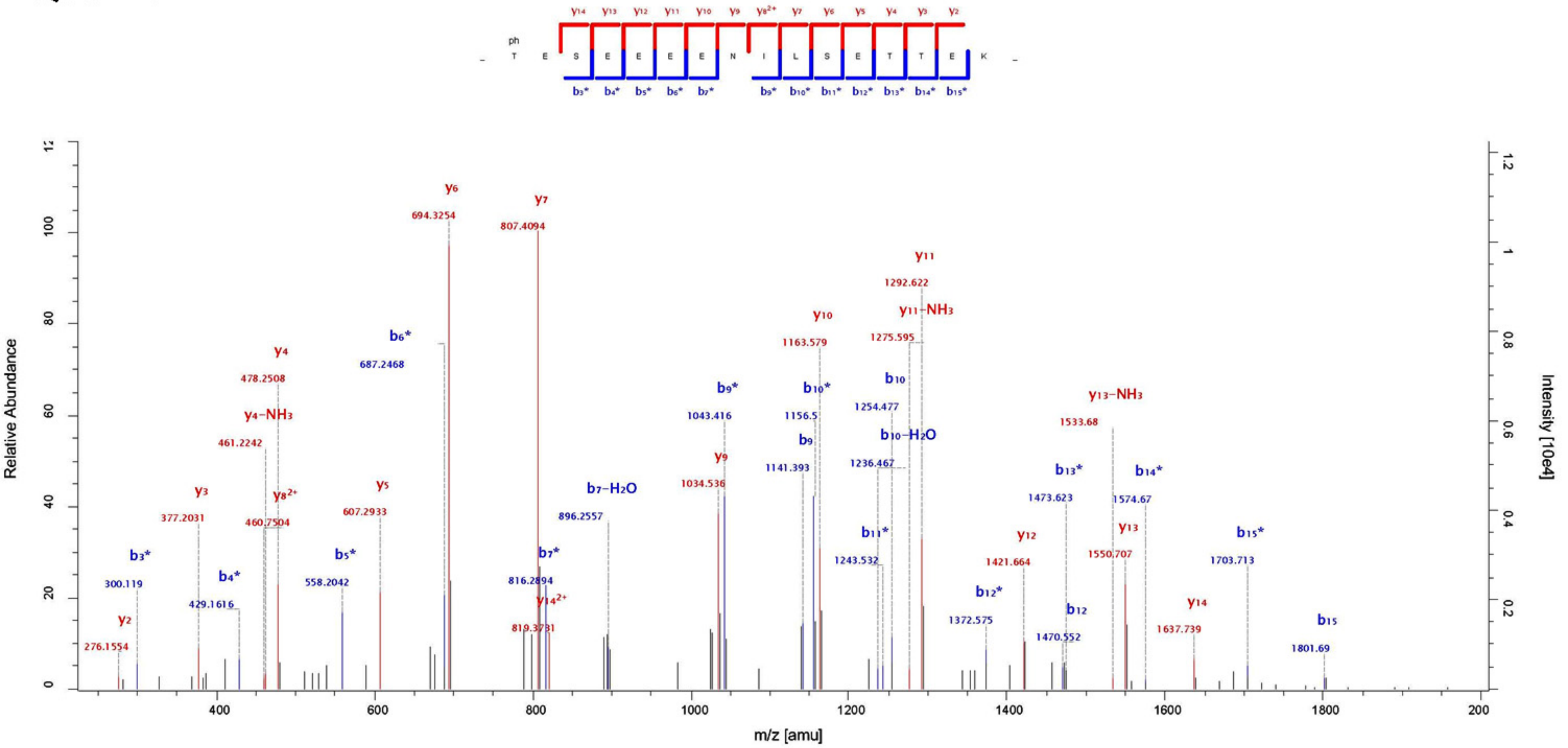

ph
T E S E E E E N I L S E T T E K
y14 y13 y12 y11 y10 y9 y8 2+ y7 y6 y5 y4 y3 y2
b3* b4* b5* b6* b7* b9* b10* b11* b12* b13* b14* b15*
Relative Abundance
Intensity [10e4]
m/z [amu]
y6 694.3254
y7 807.4094
y11 1292.622
y11-NH3 1275.595
y10 1163.579
b6* 687.2468
y4 478.2508
y4-NH3 461.2242
b9* 1043.416
b10* 1156.5
b10 1254.477
b10-H2O 1236.467
b9 1141.393
y9 1034.536
y13-NH3 1533.68
y3 377.2031
y8 2+ 460.7504
y5 607.2933
b7-H2O 896.2557
b13* 1473.623
b14* 1574.67
b3* 300.119
b4* 429.1616
b5* 558.2042
b7* 816.2894
y14 2+ 819.3731
b11* 1243.532
y12 1421.664
y13 1550.707
b15* 1703.713
b12* 1372.575
b12 1470.552
y14 1637.739
b15 1801.69
y2 276.1554

# Q6UZD6

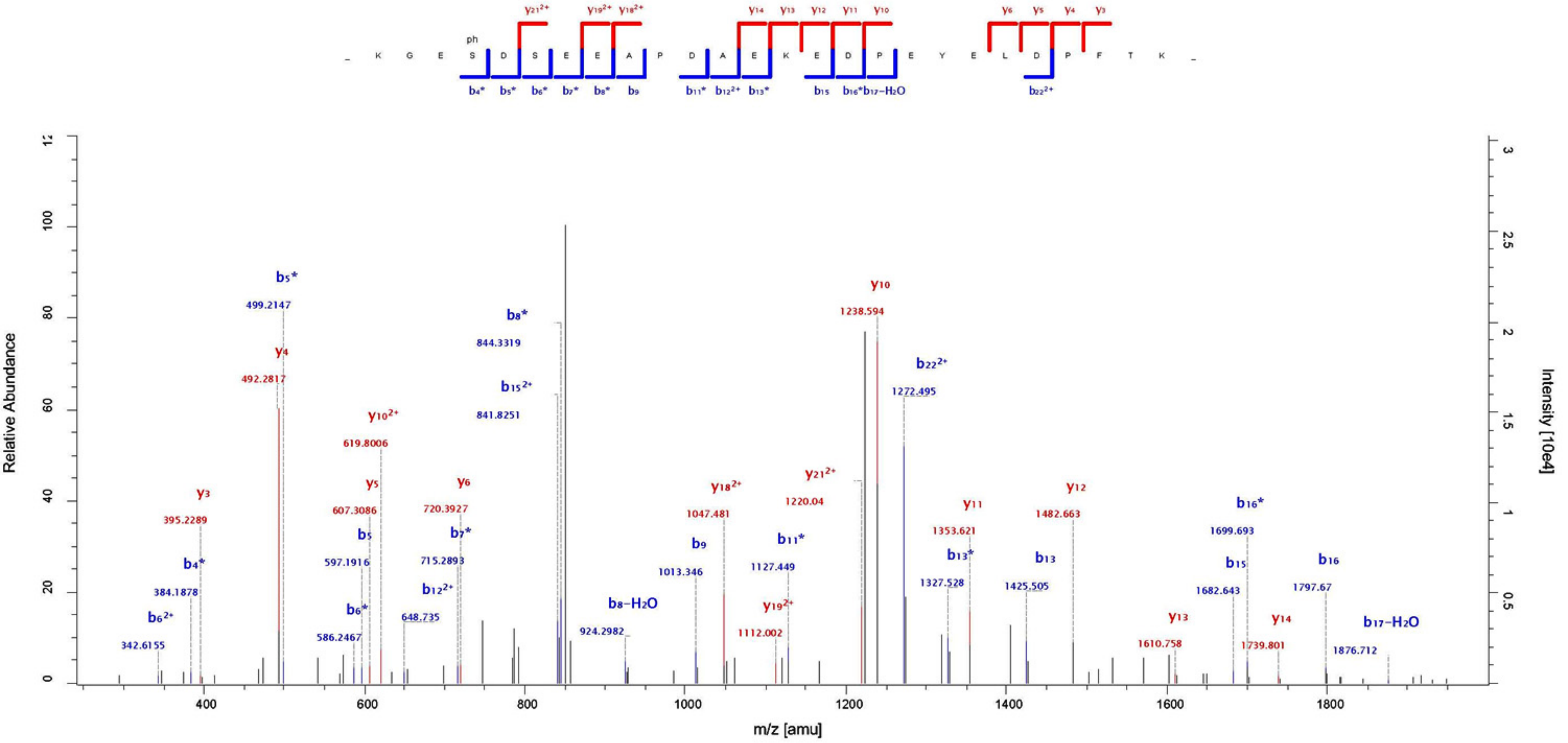

_ K G E S D S E E A P D A E K E D P E Y E L D P F T K _
ph
Relative Abundance
Intensity [10e4]
m/z [amu]
b5* 499.2147
y4 492.2817
b8* 844.3319
b15 2+ 841.8251
y10 1238.594
b22 2+ 1272.495
y10 2+ 619.8006
y3 395.2289
b4* 384.1878
b6 2+ 342.6155
y5 607.3086
b5 597.1916
b6* 586.2467
y6 720.3927
b7* 715.2893
b12 2+ 648.735
b8-H2O 924.2982
y18 2+ 1047.481
b9 1013.346
y21 2+ 1220.04
b11* 1127.449
y19 2+ 1112.002
y11 1353.621
b13* 1327.528
y12 1482.663
b13 1425.505
b16* 1699.693
b15 1682.643
b16 1797.67
y13 1610.758
y14 1739.801
b17-H2O 1876.712

# Q8GV49/ Q8GV47

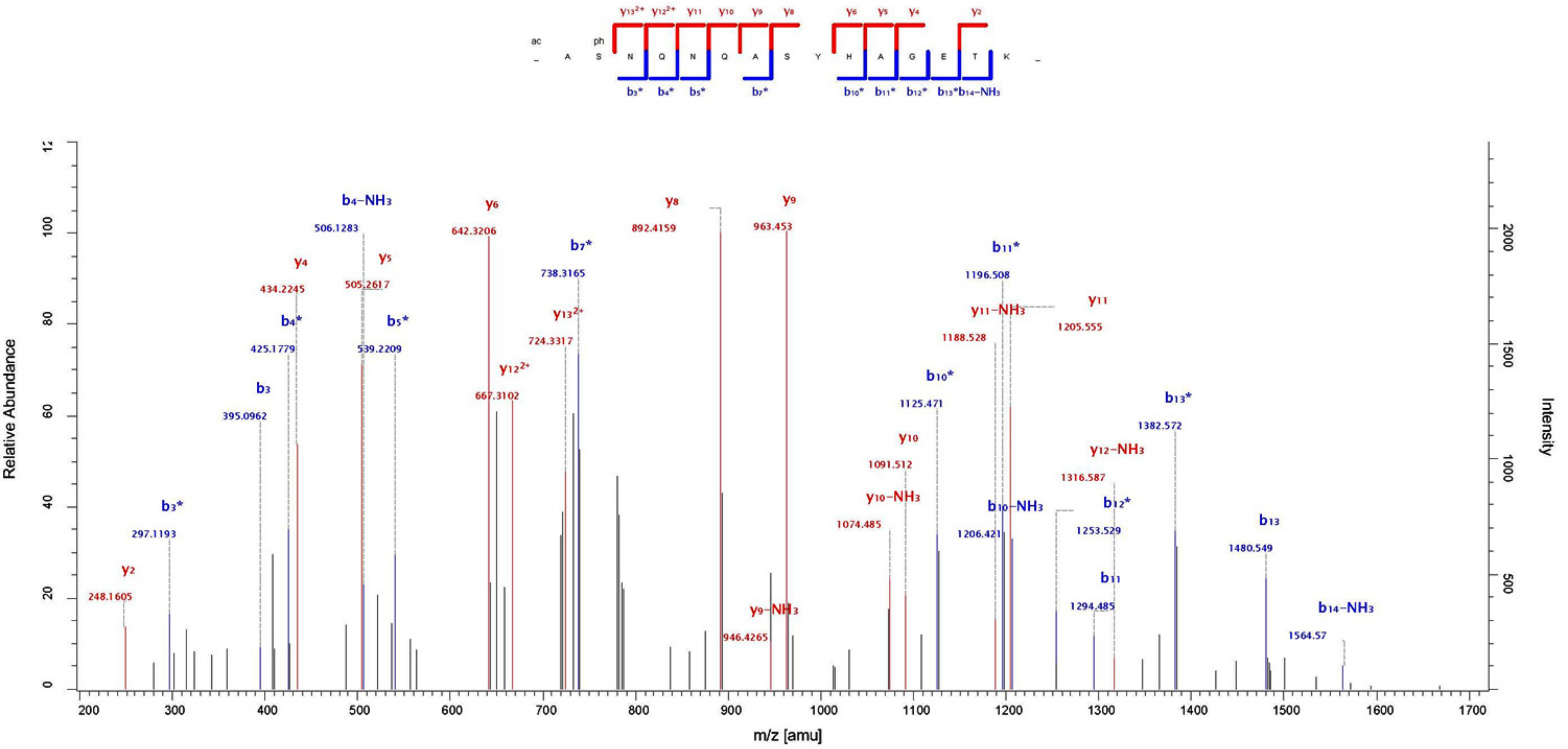

# Q8GV49

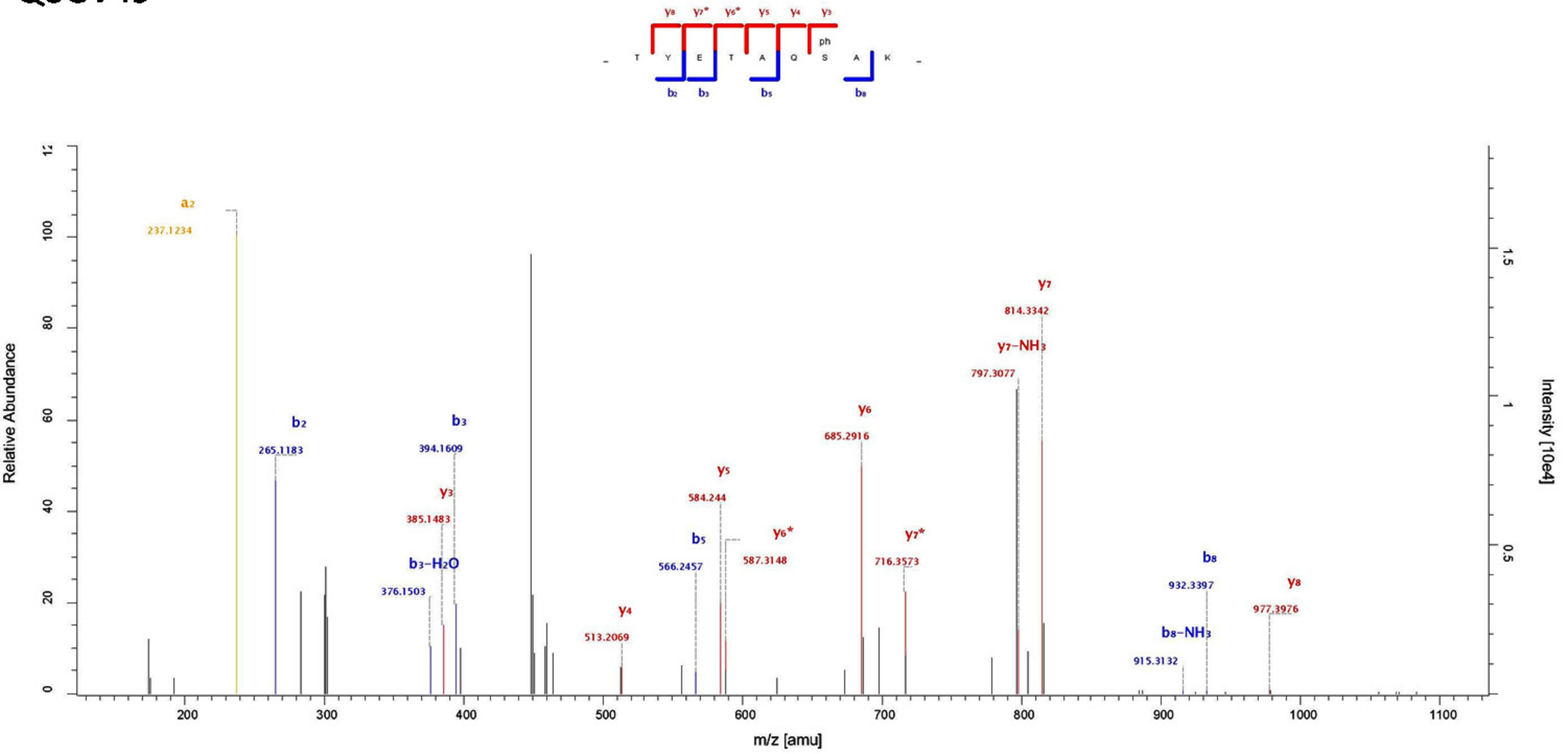

y8 y7* y6* y5 y4 y3
ph
T Y E T A Q S A K
b2 b3 b5 b8
a2
237.1234
b2
265.1183
b3
394.1609
y3
385.1483
b3-H2O
376.1503
y4
513.2069
y5
584.244
b5
566.2457
y6*
587.3148
y6
685.2916
y7*
716.3573
y7
814.3342
y7-NH3
797.3077
b8
932.3397
b8-NH3
915.3132
y8
977.3976
Relative Abundance
Intensity [10e4]
m/z [amu]

# Q8GV49/ Q8GV47

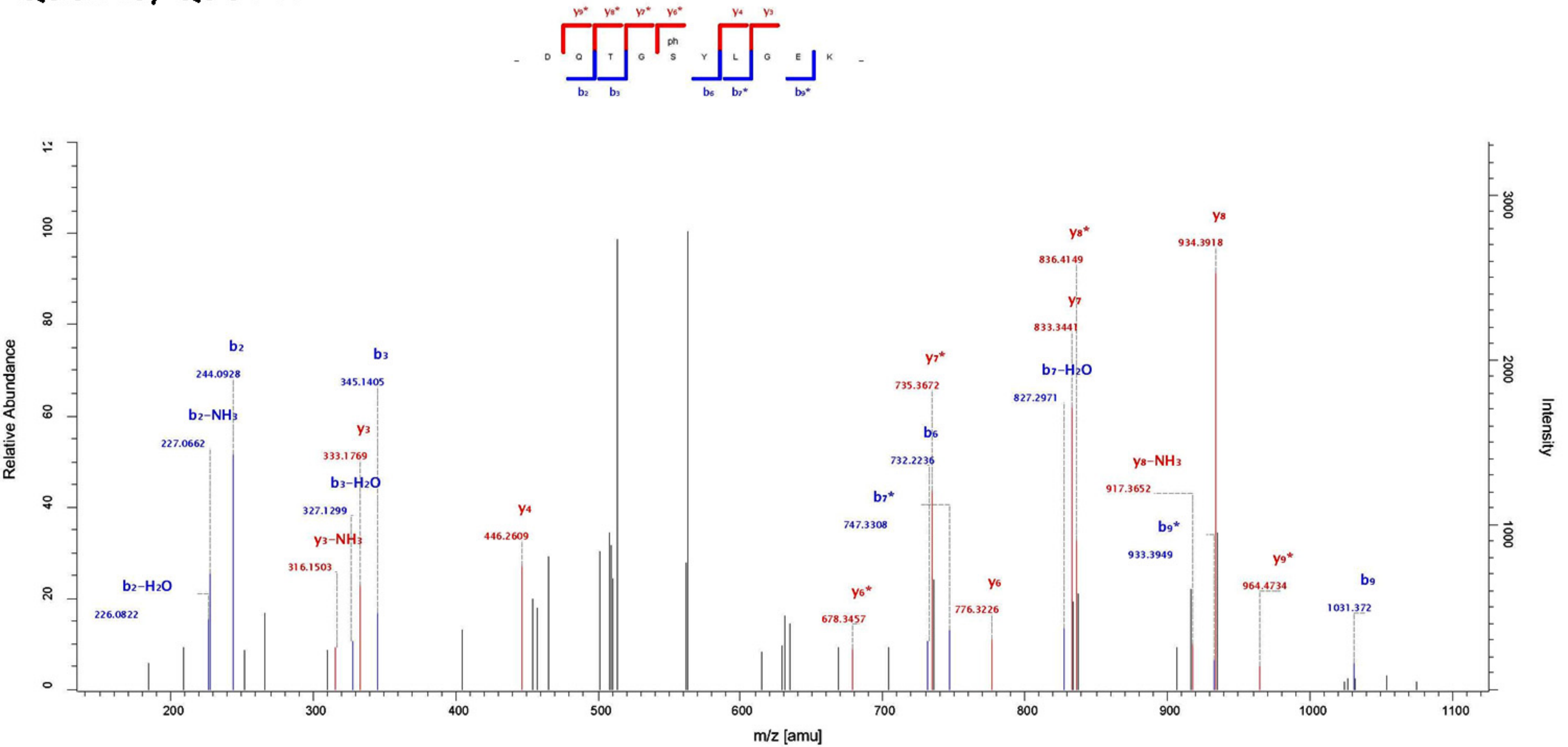

# P42755

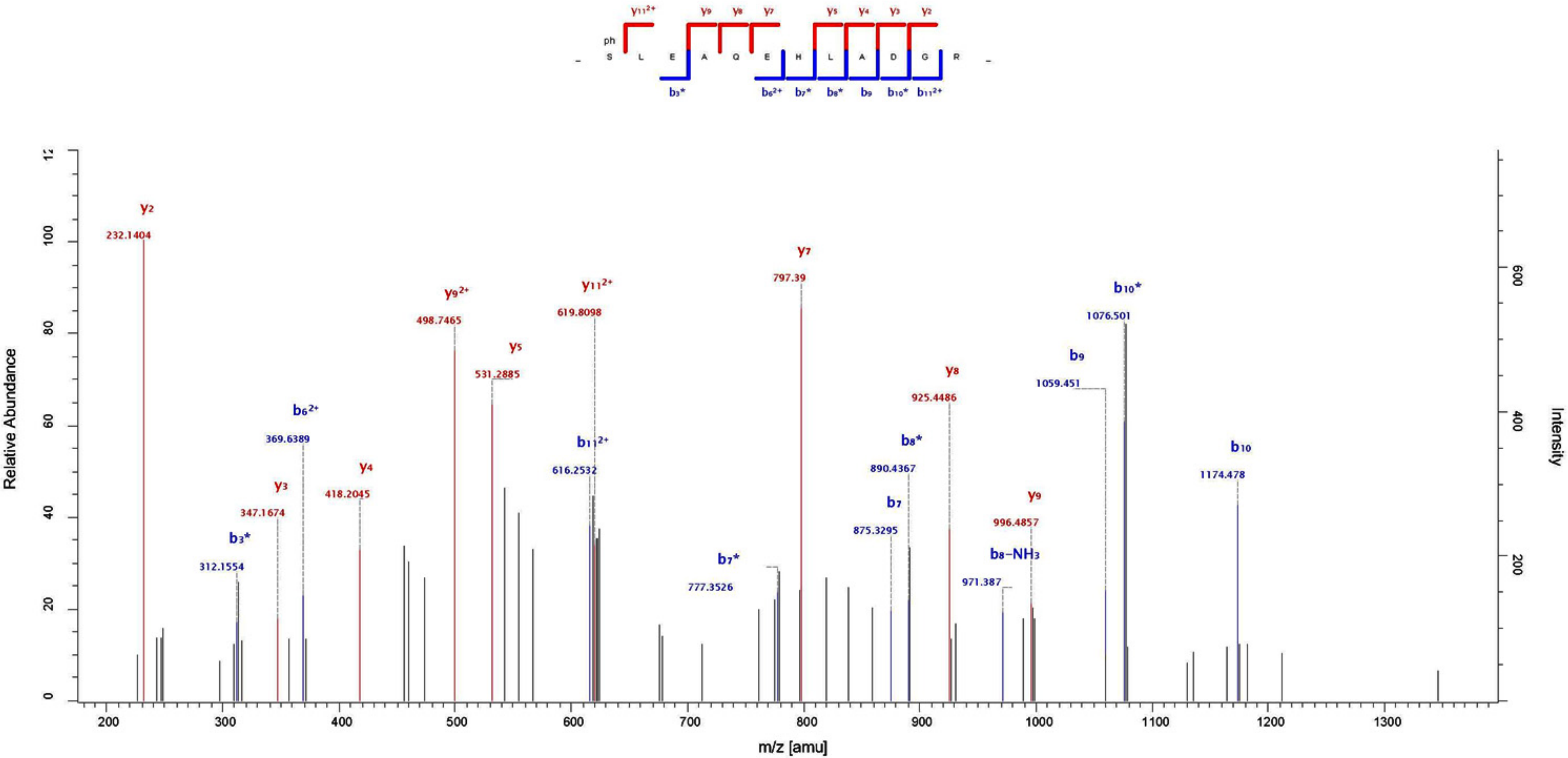

ph
S L E A Q E H L A D G R
y11 2+ y9 y8 y7 y5 y4 y3 y2
b3* b6 2+ b7* b8* b9 b10* b11 2+
Relative Abundance
Intensity
m/z [amu]
y2 232.1404
y9 2+ 498.7465
y5 531.2885
y11 2+ 619.8098
y7 797.39
b10* 1076.501
b9 1059.451
y8 925.4486
b6 2+ 369.6389
b11 2+ 616.2532
y4 418.2045
y3 347.1674
b3* 312.1554
b8* 890.4367
b7 875.3295
b10 1174.478
y9 996.4857
b8-NH3 971.387
b7* 777.3526

# P04568

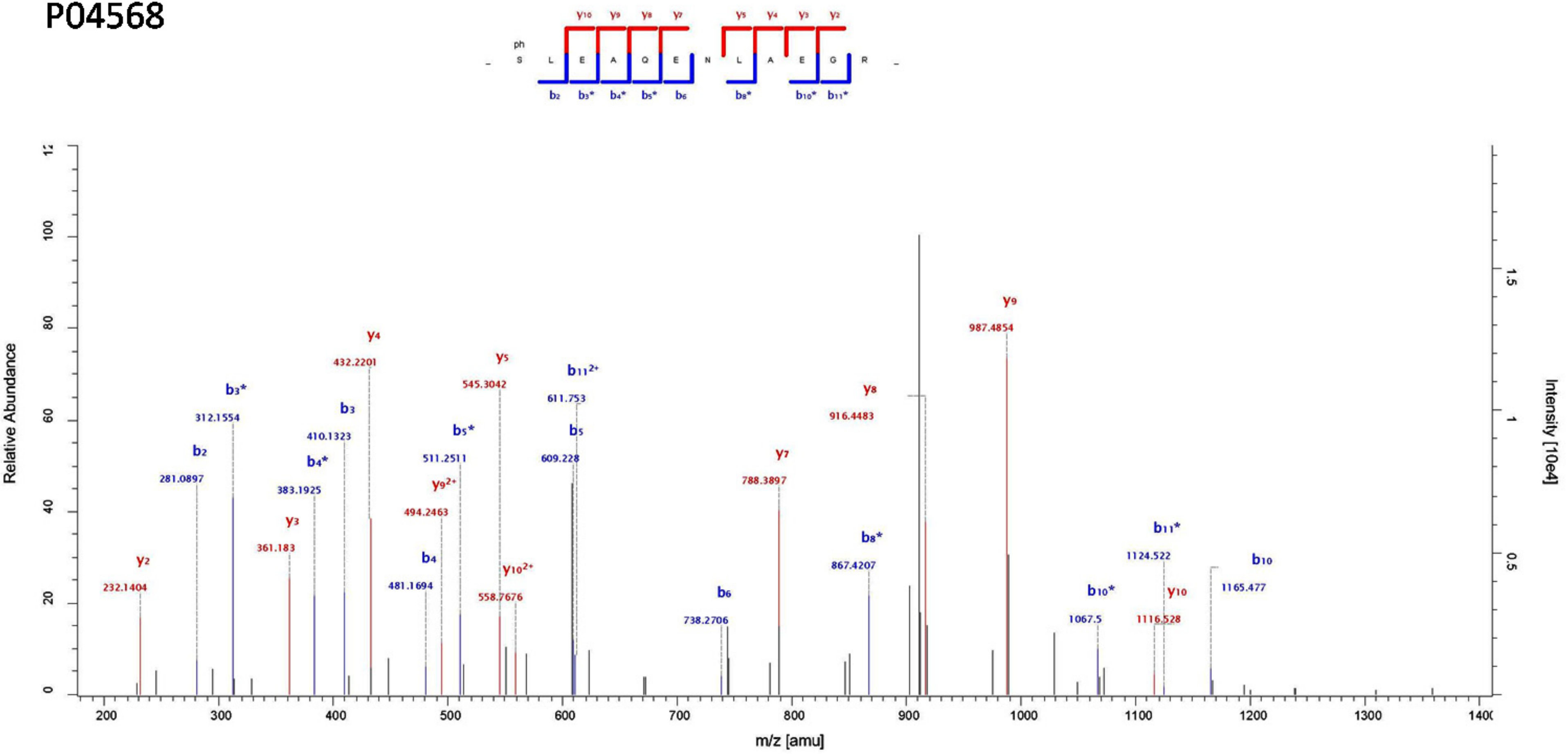

y10 y9 y8 y7 y5 y4 y3 y2
ph
S L E A Q E N L A E G R
b2 b3* b4* b5* b6 b8* b10* b11*
Relative Abundance
Intensity [10e4]
m/z [amu]
y2 232.1404
b2 281.0897
b3* 312.1554
y3 361.183
b4* 383.1925
b3 410.1323
y4 432.2201
b4 481.1694
y9 2+ 494.2463
b5* 511.2511
y5 545.3042
y10 2+ 558.7676
b5 609.228
b11 2+ 611.753
b6 738.2706
y7 788.3897
b8* 867.4207
y8 916.4483
y9 987.4854
b10* 1067.5
y10 1116.528
b11* 1124.522
b10 1165.477

# C0LF32/ H9AXB3/ Q41593/ Q9ST57

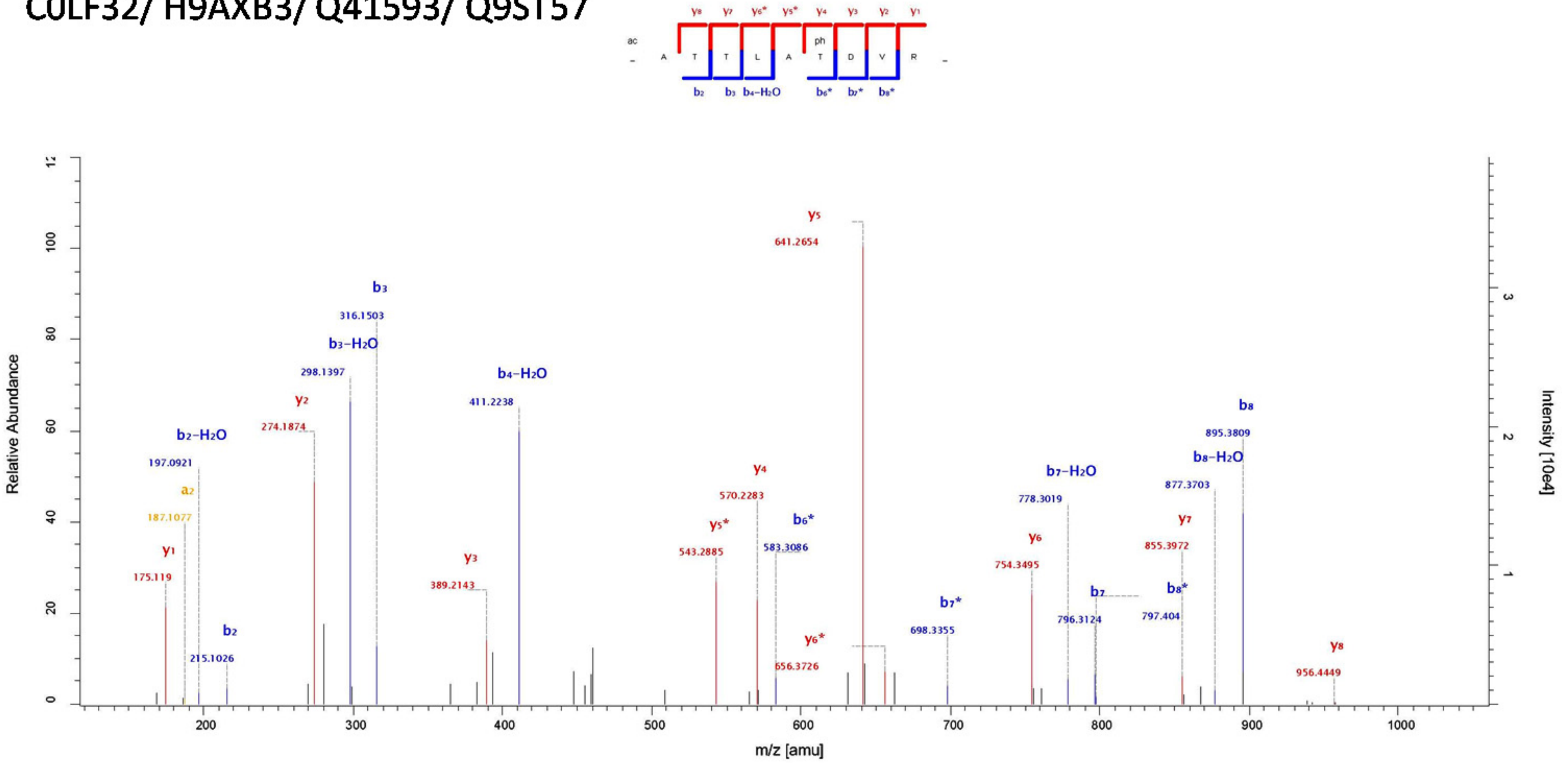

## C0LF32/ Q41593

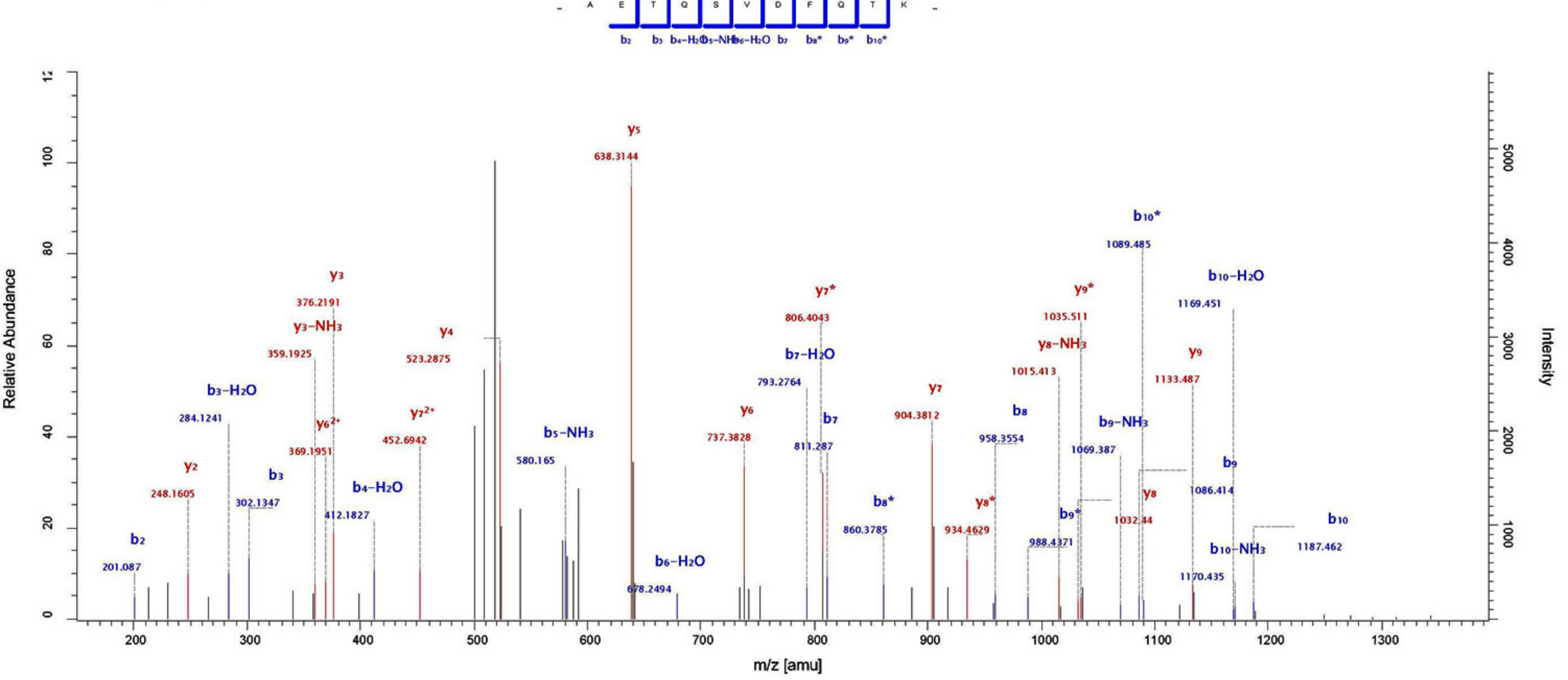

# H9AXB3/ Q9ST57

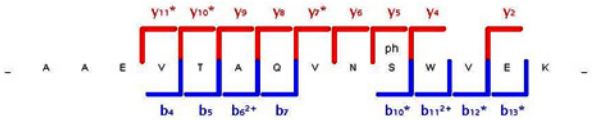


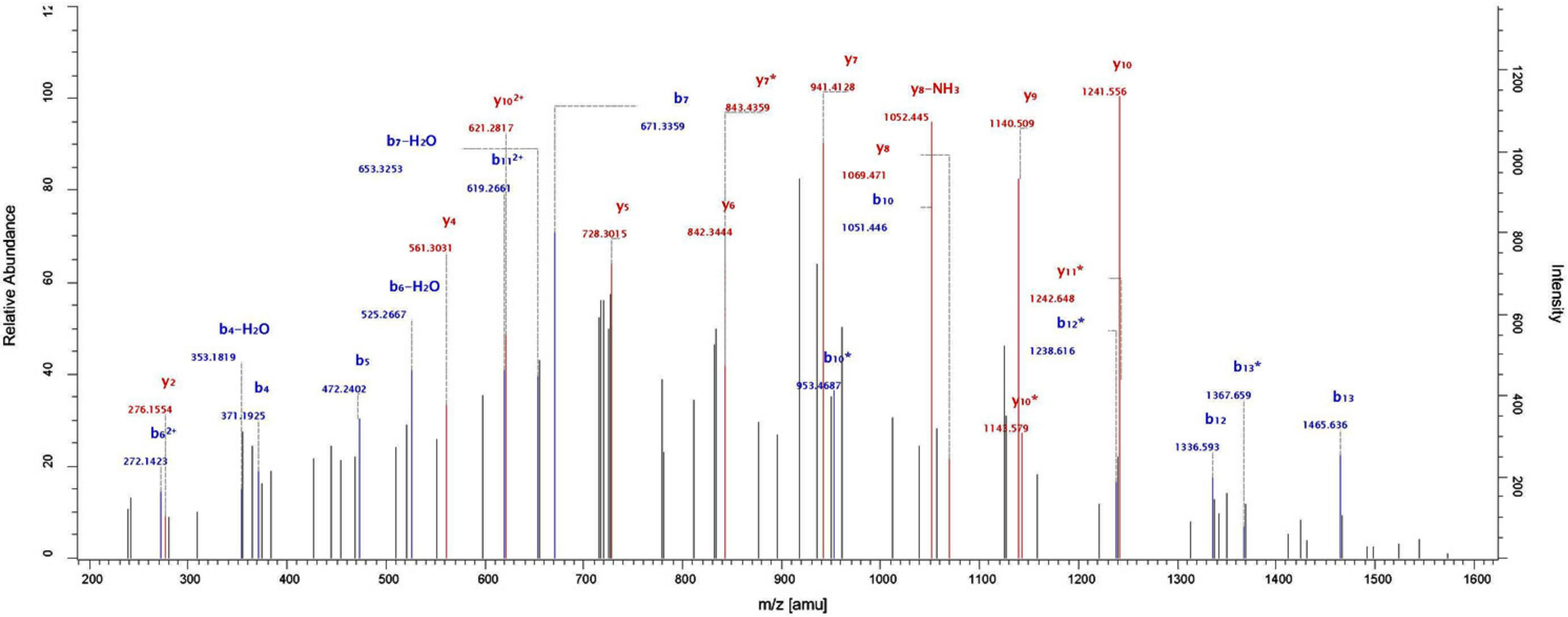

# H9AXB3/ Q9ST57

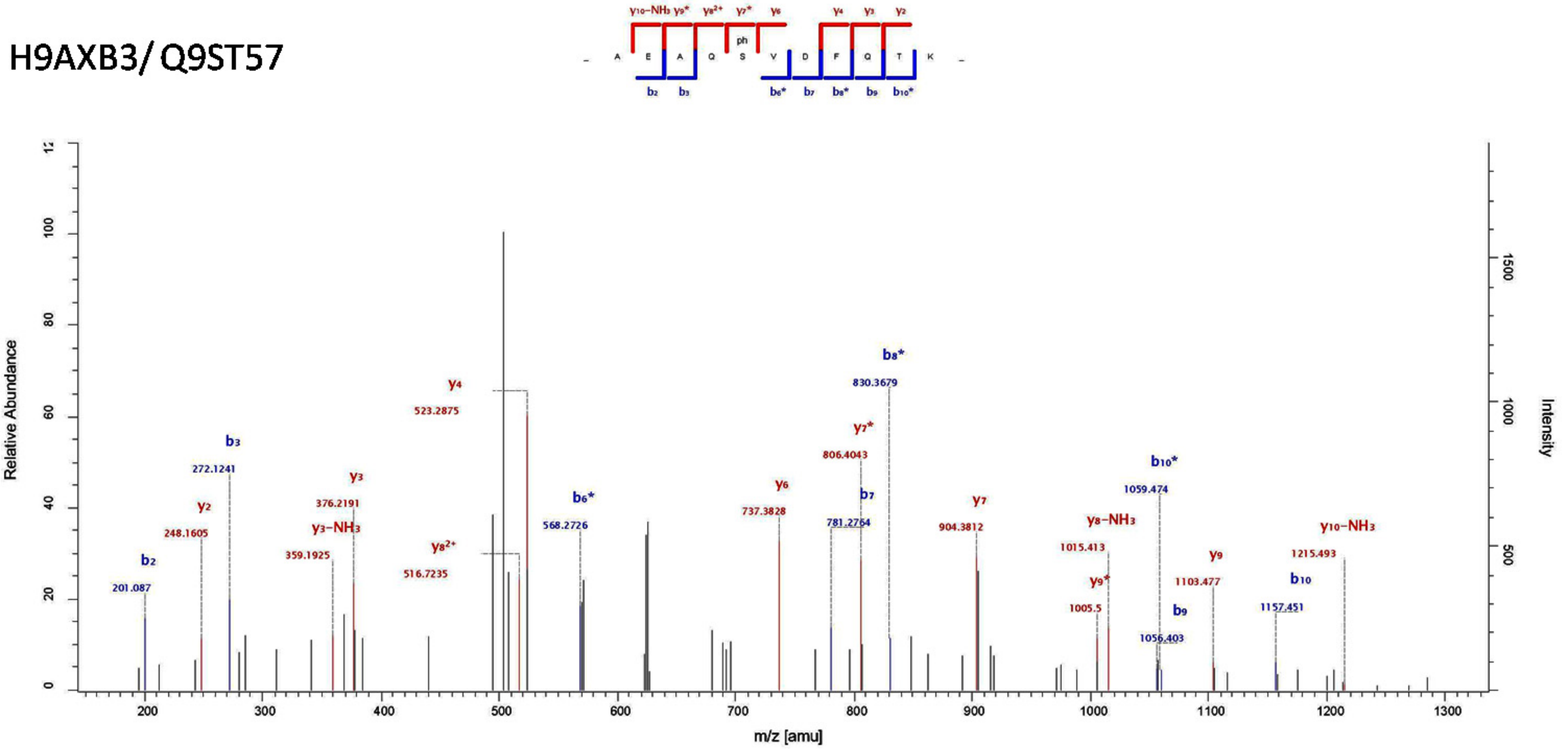

# H9AXB3

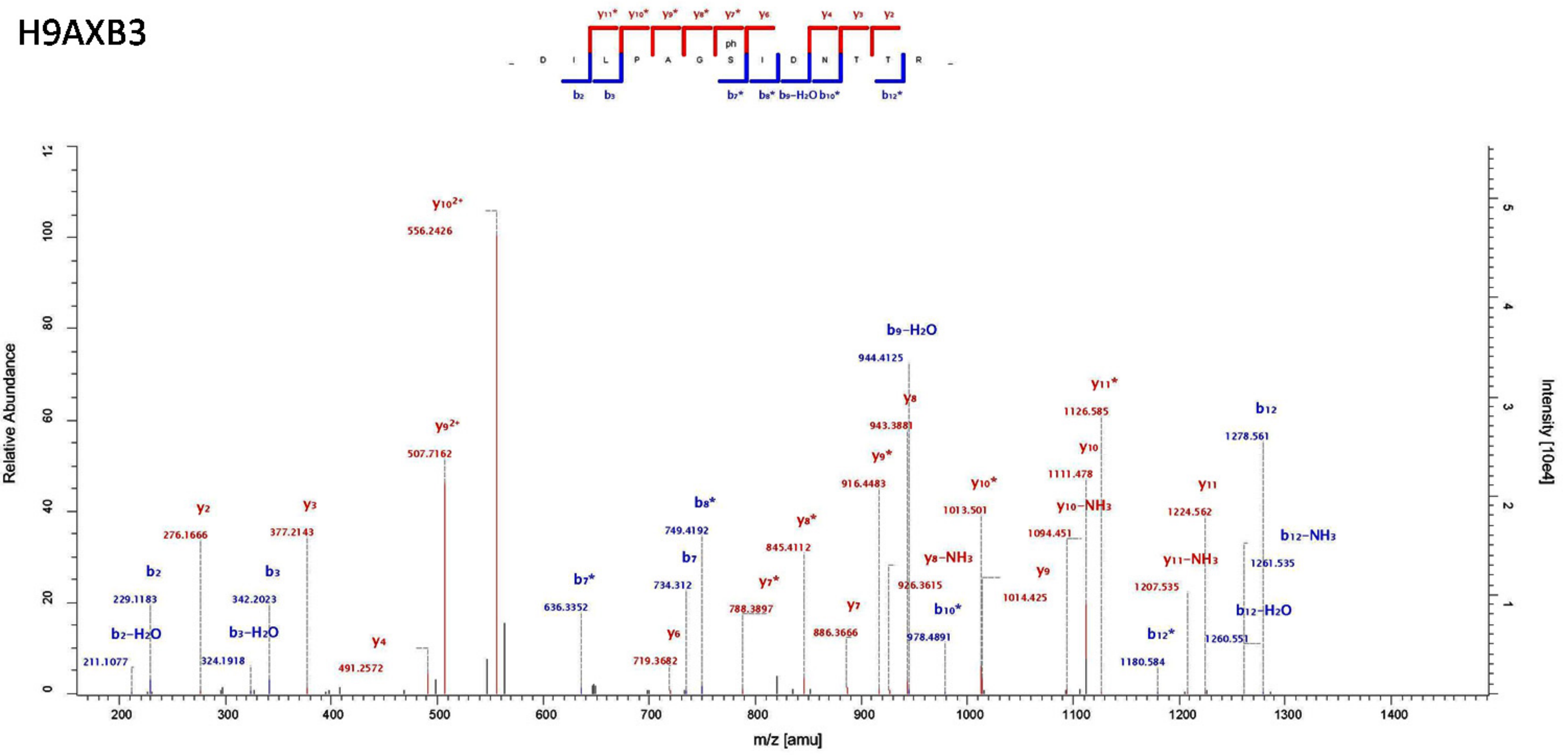

ph
D I L P A G S I D N T T R
b2 b3 b7* b8* b9-H2O b10* b12*
y11* y10* y9* y8* y7* y6 y4 y3 y2
Relative Abundance
Intensity [10e4]
m/z [amu]
y10 2+
556.2426
y9 2+
507.7162
b9-H2O
944.4125
y11*
1126.585
y8
943.3881
b12
1278.561
y10
1111.478
y9*
916.4483
y10*
1013.501
y11
1224.562
y10-NH3
1094.451
b12-NH3
1261.535
b8*
749.4192
y2
276.1666
y3
377.2143
y8*
845.4112
y8-NH3
926.3615
y11-NH3
1207.535
b7
734.312
b2
229.1183
b3
342.2023
b7*
636.3352
y7*
788.3897
y9
1014.425
y7
886.3666
b10*
978.4891
b12-H2O
1260.551
b2-H2O
211.1077
b3-H2O
324.1918
y4
491.2572
y6
719.3682
b12*
1180.584

# E0WC53

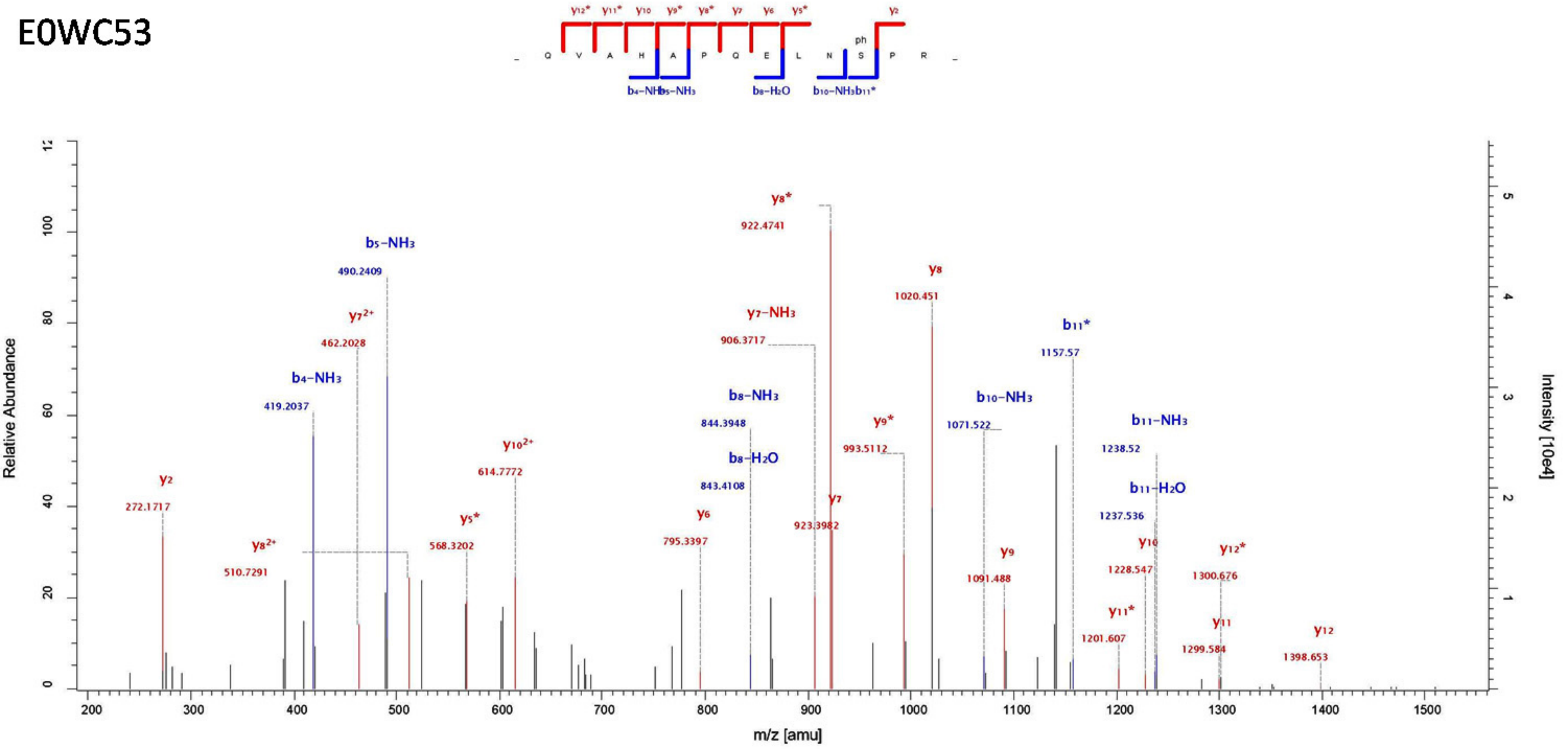

_ Q V A H A P Q E L N S P R _
ph
y12* y11* y10 y9* y8* y7 y6 y5* y2
b4-NH3 b5-NH3 b8-H2O b10-NH3 b11*
Relative Abundance
Intensity [10e4]
m/z [amu]
y2 272.1717
y8 2+ 510.7291
b4-NH3 419.2037
y7 2+ 462.2028
b5-NH3 490.2409
y5* 568.3202
y10 2+ 614.7772
y6 795.3397
b8-NH3 844.3948
b8-H2O 843.4108
y7-NH3 906.3717
y8* 922.4741
y7 923.3982
y9* 993.5112
y8 1020.451
b10-NH3 1071.522
y9 1091.488
b11* 1157.57
y11* 1201.607
y10 1228.547
b11-NH3 1238.52
b11-H2O 1237.536
y11 1299.584
y12* 1300.676
y12 1398.653

E0WC53

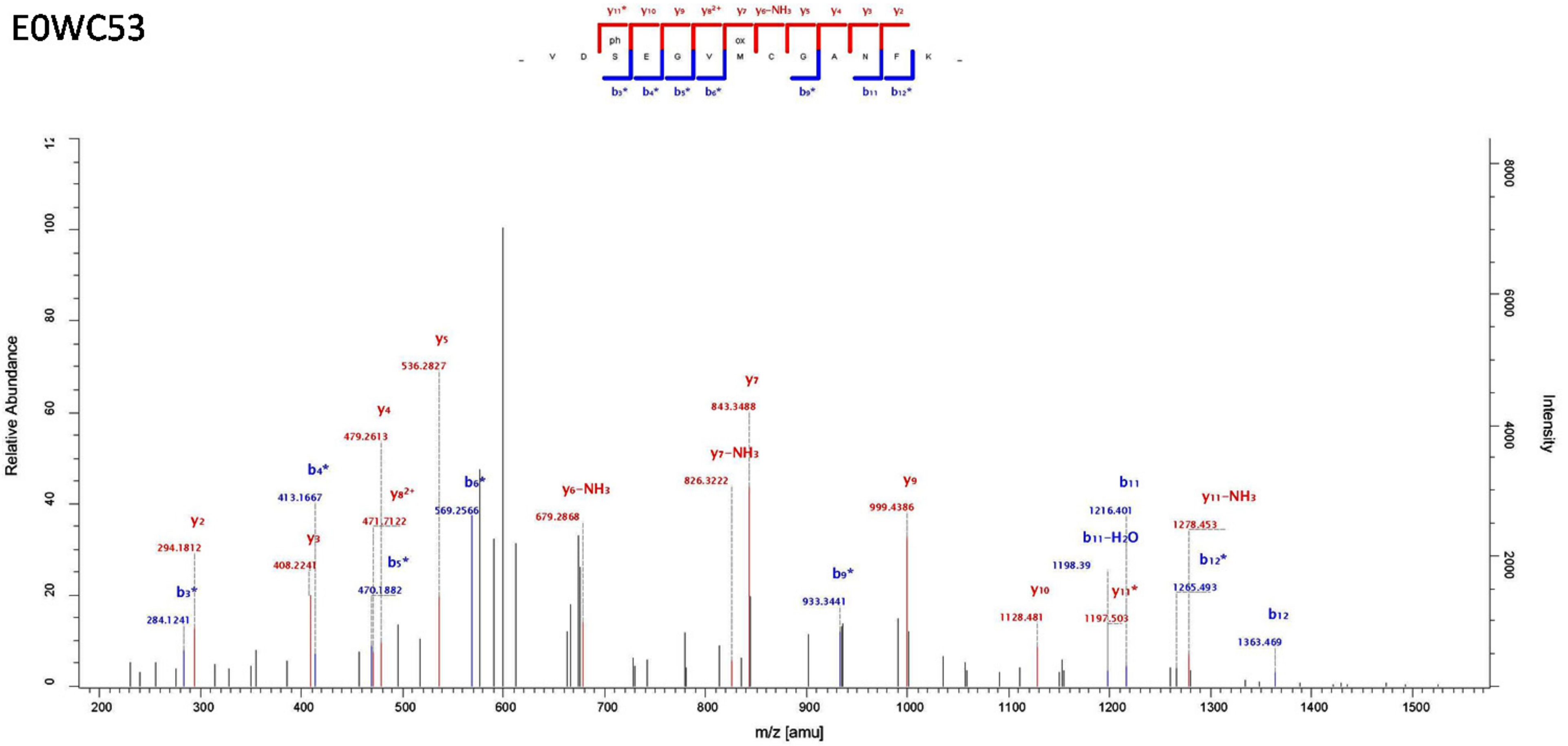

y11* y10 y9 y8 2+ y7 y6-NH3 y5 y4 y3 y2
ph
ox
_ V D S E G V M C G A N F K _
b3* b4* b5* b6* b9* b11 b12*
Relative Abundance
Intensity
m/z [amu]
y2 294.1812
b3* 284.1241
y3 408.2241
b4* 413.1667
y4 479.2613
y8 2+ 471.7122
b5* 470.1882
y5 536.2827
b6* 569.2566
y6-NH3 679.2868
y7 843.3488
y7-NH3 826.3222
b9* 933.3441
y9 999.4386
y10 1128.481
b11 1216.401
b11-H2O 1198.39
y11* 1197.503
y11-NH3 1278.453
b12* 1265.493
b12 1363.469

E0WC53

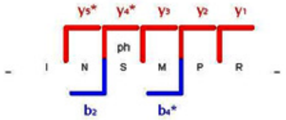
y5* y4* y3 y2 y1
ph
I N S M P R
b2 b4*

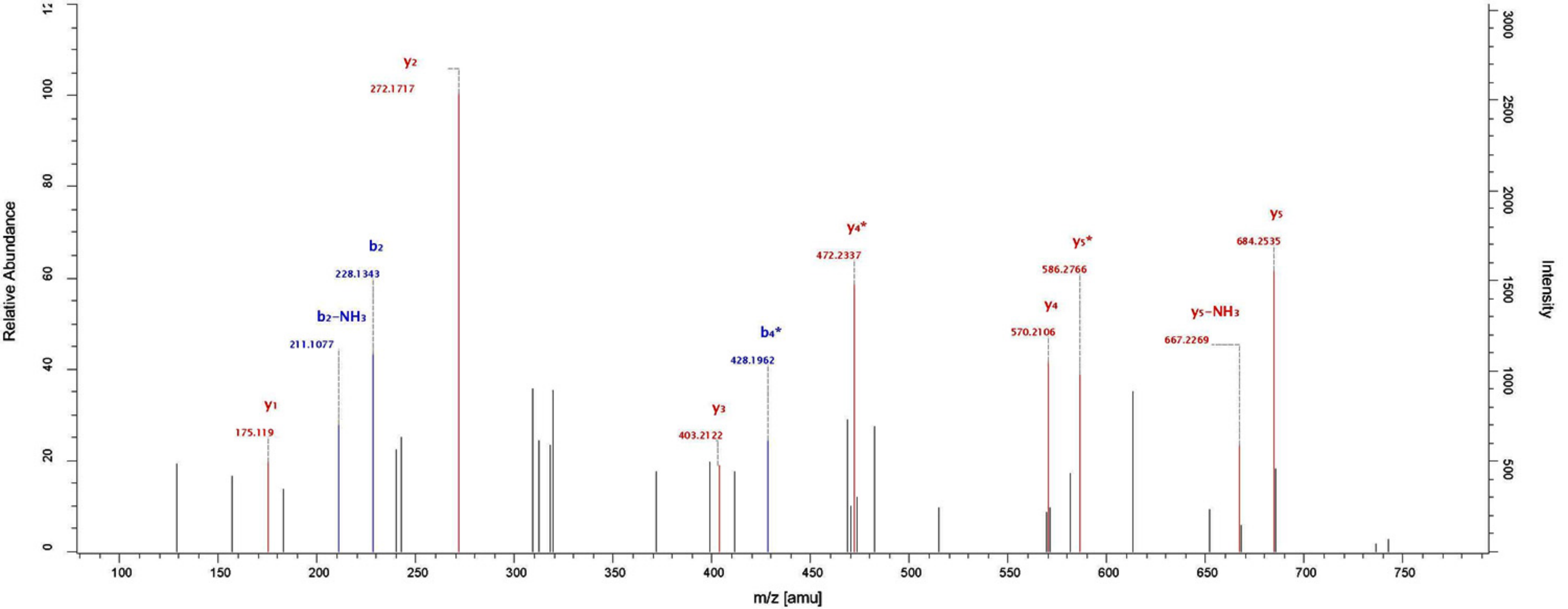
Relative Abundance
Intensity
m/z [amu]
y2
272.1717
b2
228.1343
b2-NH3
211.1077
y1
175.119
y3
403.2122
b4*
428.1962
y4*
472.2337
y4
570.2106
y5*
586.2766
y5-NH3
667.2269
y5
684.2535

# F4Y591

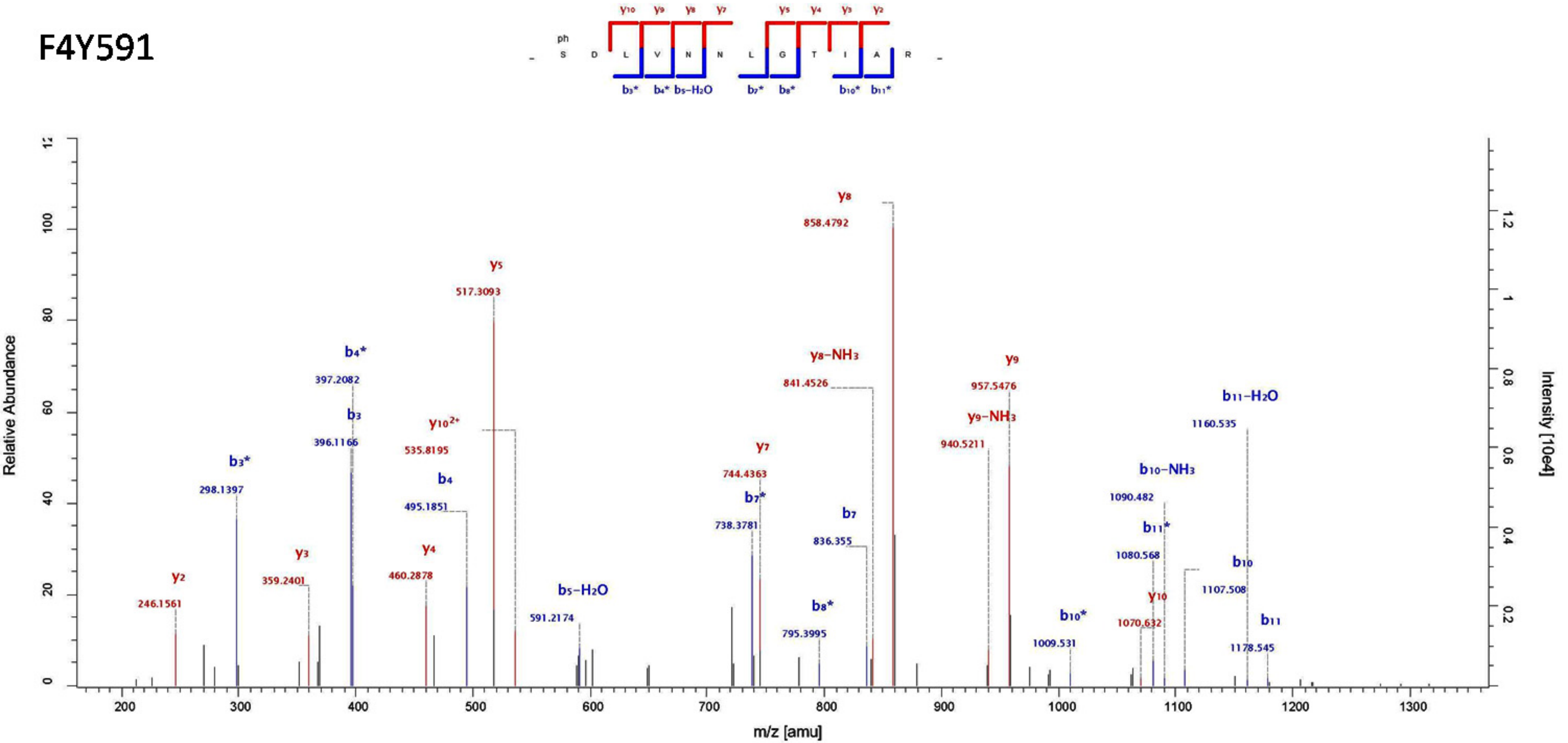

ph
S D L V N N L G T I A R
y10 y9 y8 y7 y5 y4 y3 y2
b3* b4* b5-H2O b7* b8* b10* b11*
Relative Abundance
Intensity [10e4]
m/z [amu]
y2 246.1561
b3* 298.1397
y3 359.2401
b3 396.1166
b4* 397.2082
y4 460.2878
b4 495.1851
y5 517.3093
y10 2+ 535.8195
b5-H2O 591.2174
b7* 738.3781
y7 744.4363
b8* 795.3995
b7 836.355
y8-NH3 841.4526
y8 858.4792
y9-NH3 940.5211
y9 957.5476
b10* 1009.531
y10 1070.632
b11* 1080.568
b10-NH3 1090.482
b10 1107.508
b11-H2O 1160.535
b11 1178.545

F4Y591

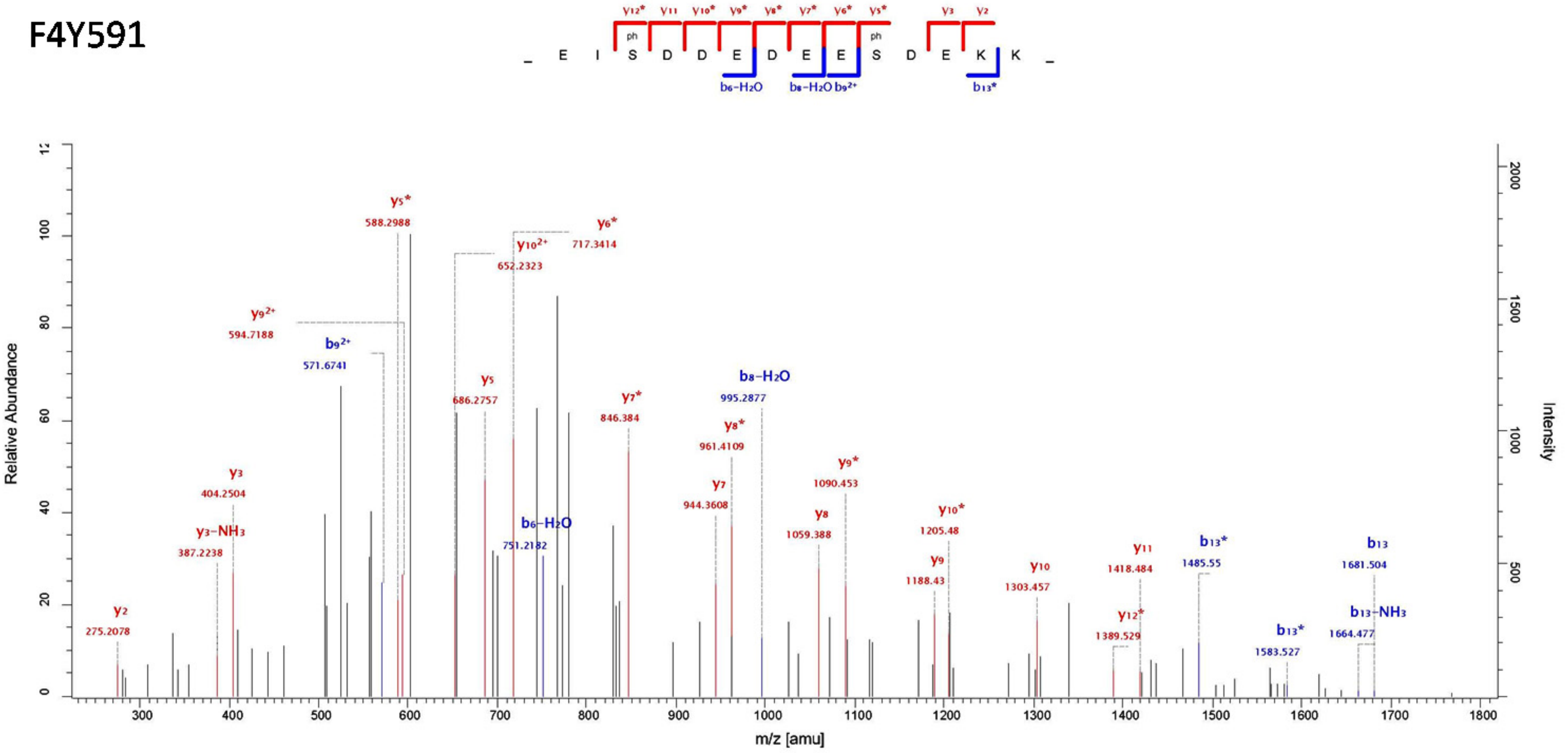

F4Y591

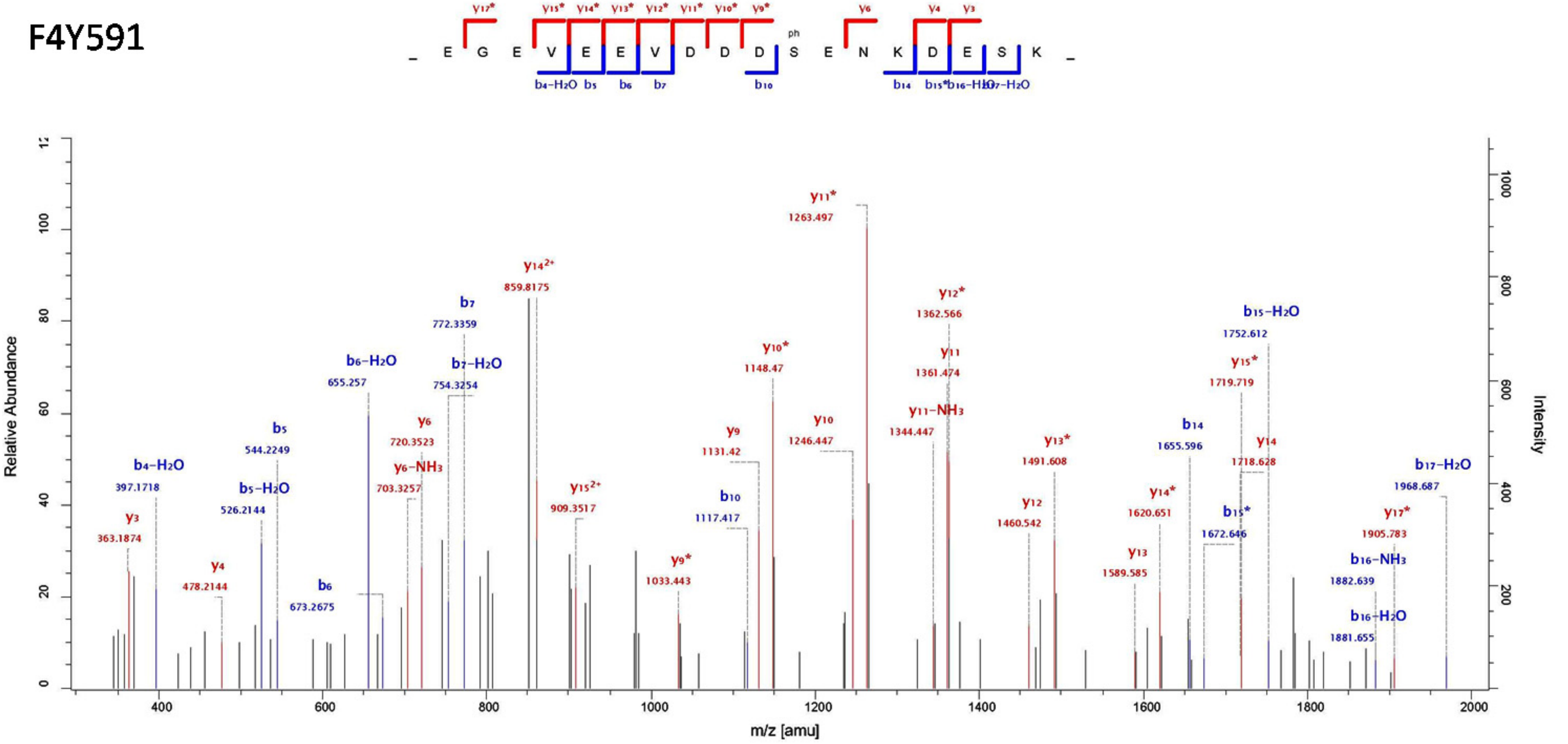

_ E G E V E E V D D D S E N K D E S K _
ph
y17* y15* y14* y13* y12* y11* y10* y9* y6 y4 y3
b4-H2O b5 b6 b7 b10 b14 b15* b16-H2O b17-H2O
Relative Abundance
Intensity
m/z [amu]
y3 363.1874
b4-H2O 397.1718
y4 478.2144
b5-H2O 526.2144
b5 544.2249
b6-H2O 655.257
b6 673.2675
y6-NH3 703.3257
y6 720.3523
b7-H2O 754.3254
b7 772.3359
y14 2+ 859.8175
y15 2+ 909.3517
y9* 1033.443
b10 1117.417
y9 1131.42
y10* 1148.47
y10 1246.447
y11* 1263.497
y11-NH3 1344.447
y11 1361.474
y12* 1362.566
y12 1460.542
y13* 1491.608
y13 1589.585
y14* 1620.651
b14 1655.596
b15* 1672.646
y14 1718.628
y15* 1719.719
b15-H2O 1752.612
b16-H2O 1881.655
b16-NH3 1882.639
y17* 1905.783
b17-H2O 1968.687

# Q8LRU5

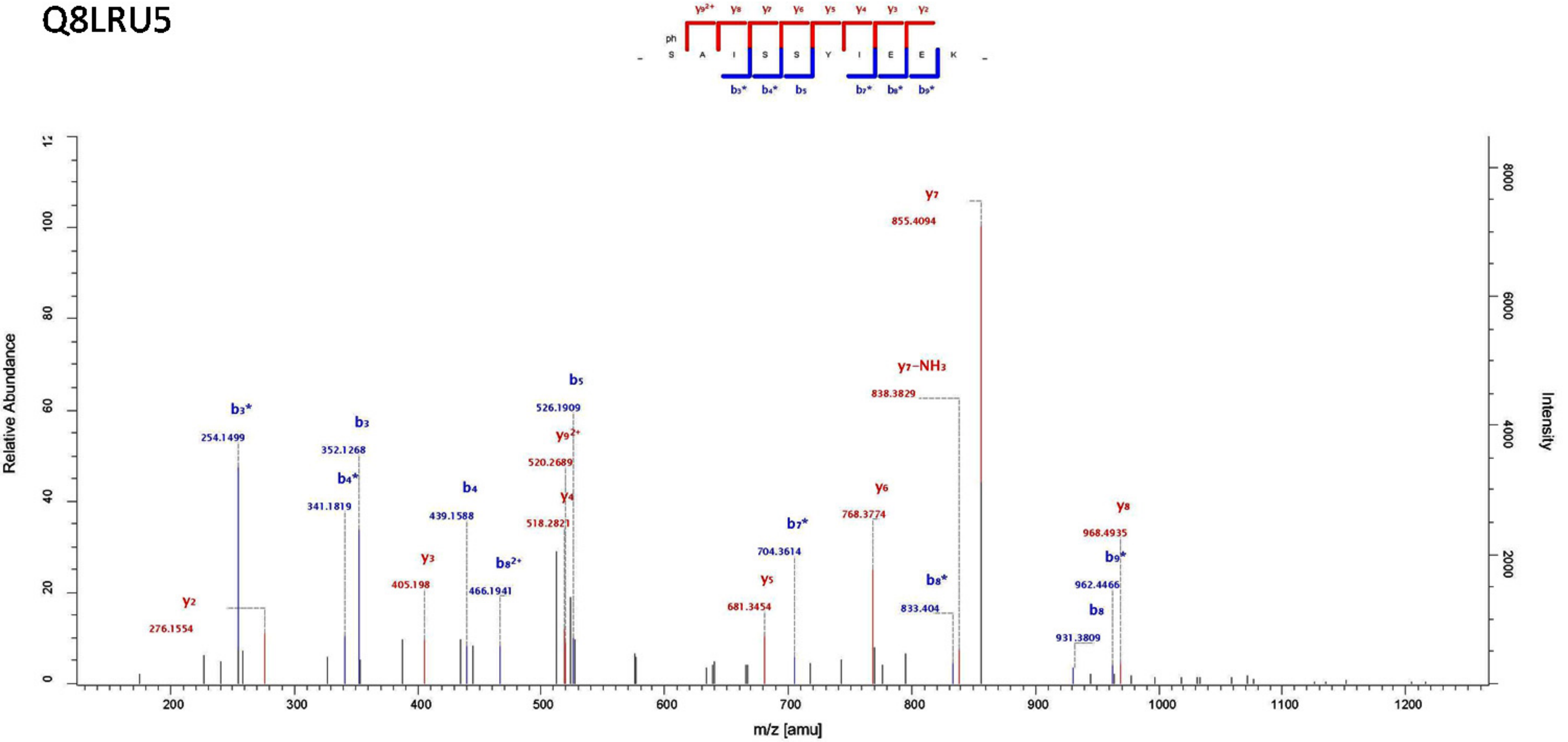

ph
S A I S S Y I E E K
y9 2+ y8 y7 y6 y5 y4 y3 y2
b3* b4* b5 b7* b8* b9*
Relative Abundance
Intensity
m/z [amu]
y7
855.4094
y7-NH3
838.3829
b5
526.1909
b3*
254.1499
b3
352.1268
y9 2+
520.2689
b4*
341.1819
b4
439.1588
y4
518.2821
y6
768.3774
b7*
704.3614
y8
968.4935
y3
405.198
b8 2+
466.1941
b9*
962.4466
y5
681.3454
b8*
833.404
y2
276.1554
b8
931.3809

# DOEY60

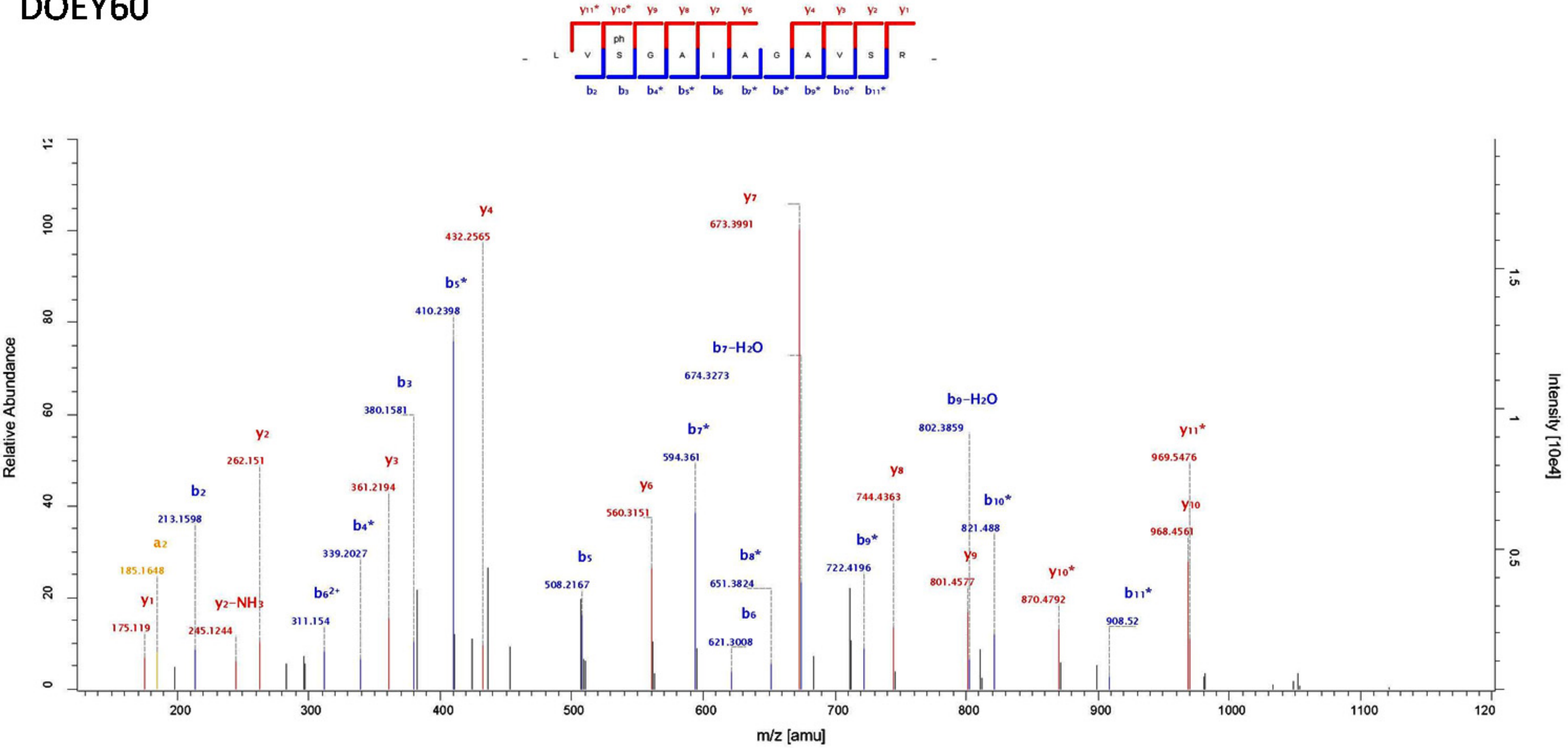

y11* y10* y9 y8 y7 y6 y4 y3 y2 y1
ph
L V S G A I A G A V S R
b2 b3 b4* b5* b6 b7* b8* b9* b10* b11*
Relative Abundance
Intensity [10e4]
m/z [amu]
y1 175.119
a2 185.1648
b2 213.1598
y2-NH3 245.1244
y2 262.151
b6 2+ 311.154
b4* 339.2027
y3 361.2194
b3 380.1581
b5* 410.2398
y4 432.2565
b5 508.2167
y6 560.3151
b7* 594.361
b6 621.3008
b8* 651.3824
y7 673.3991
b7-H2O 674.3273
b9* 722.4196
y8 744.4363
y9 801.4577
b9-H2O 802.3859
b10* 821.488
y10* 870.4792
b11* 908.52
y10 968.4561
y11* 969.5476

P08823

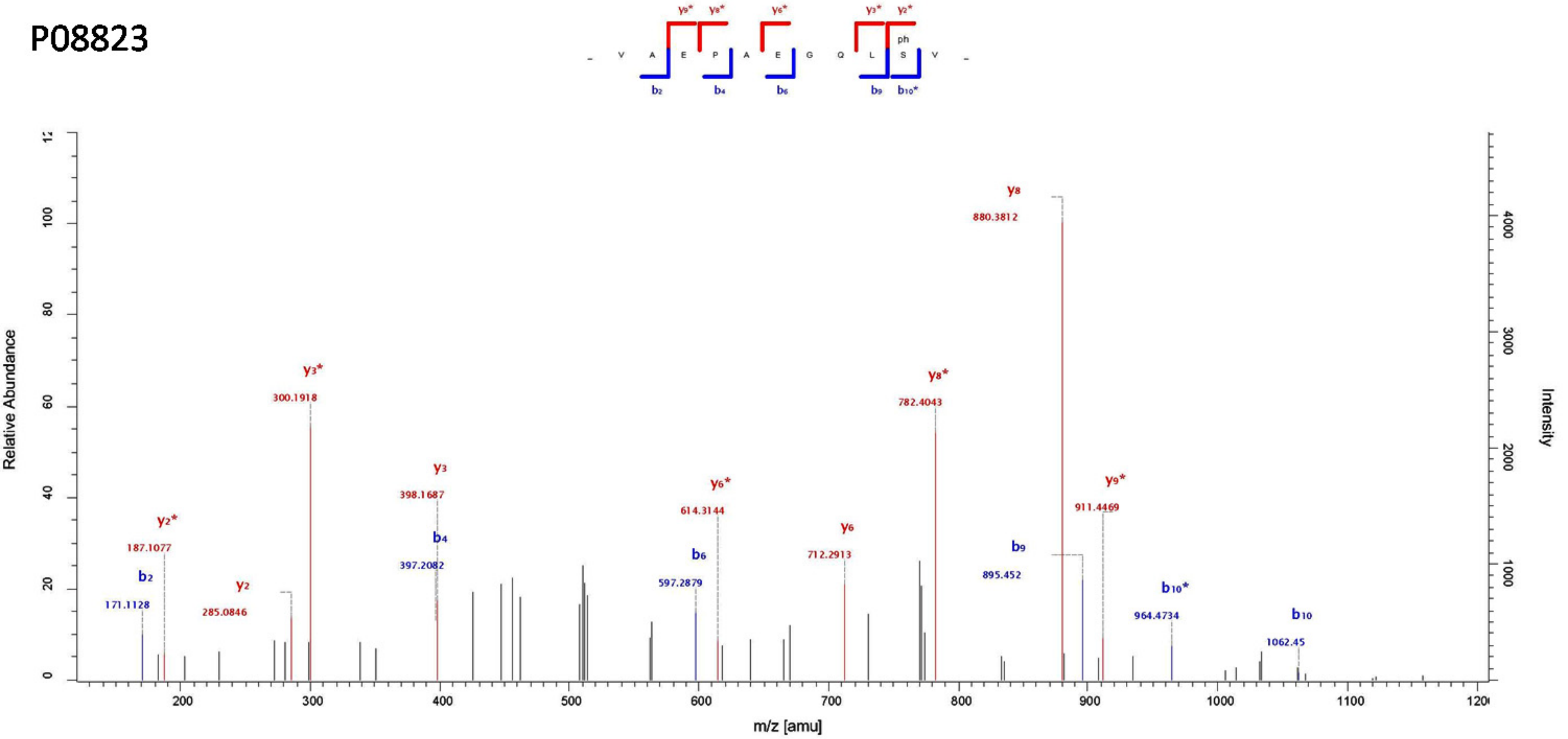

V A E P A E G Q L S V
ph
y9* y8* y6* y3* y2*
b2 b4 b6 b9 b10*
Relative Abundance
Intensity
m/z [amu]
y8
880.3812
y3*
300.1918
y8*
782.4043
y3
398.1687
b4
397.2082
y6*
614.3144
y9*
911.4469
y2*
187.1077
y6
712.2913
b9
895.452
b6
597.2879
b2
171.1128
y2
285.0846
b10*
964.4734
b10
1062.45

# Q0Q5D9

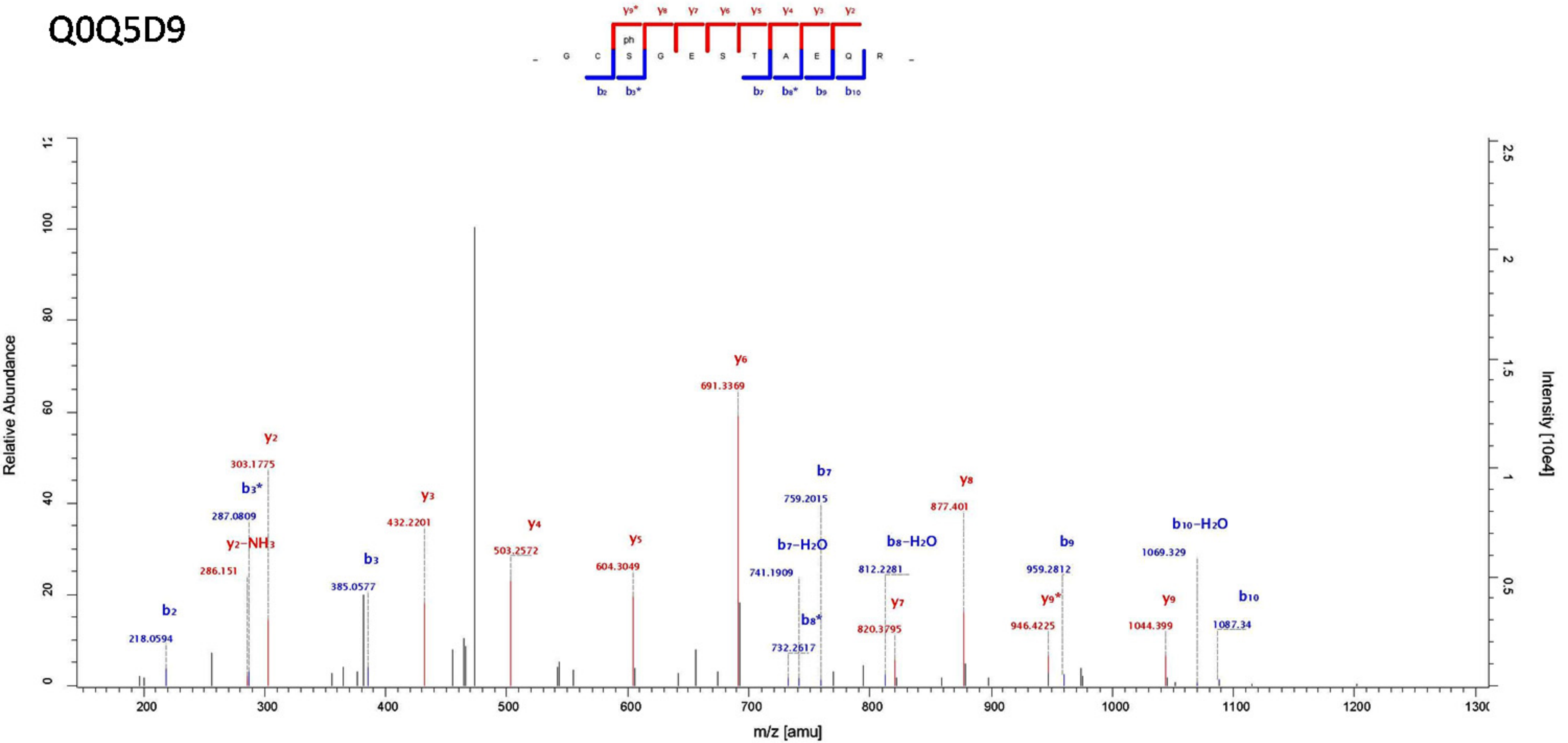

y9* y8 y7 y6 y5 y4 y3 y2
ph
G C S G E S T A E Q R
b2 b3* b7 b8* b9 b10
Relative Abundance
Intensity [10e4]
m/z [amu]
y6
691.3369
y2
303.1775
b3*
287.0809
y2-NH3
286.151
b2
218.0594
b3
385.0577
y3
432.2201
y4
503.2572
y5
604.3049
b7
759.2015
b7-H2O
741.1909
b8*
732.2617
b8-H2O
812.2281
y7
820.3795
y8
877.401
b9
959.2812
y9*
946.4225
b10-H2O
1069.329
y9
1044.399
b10
1087.34

# Q43657

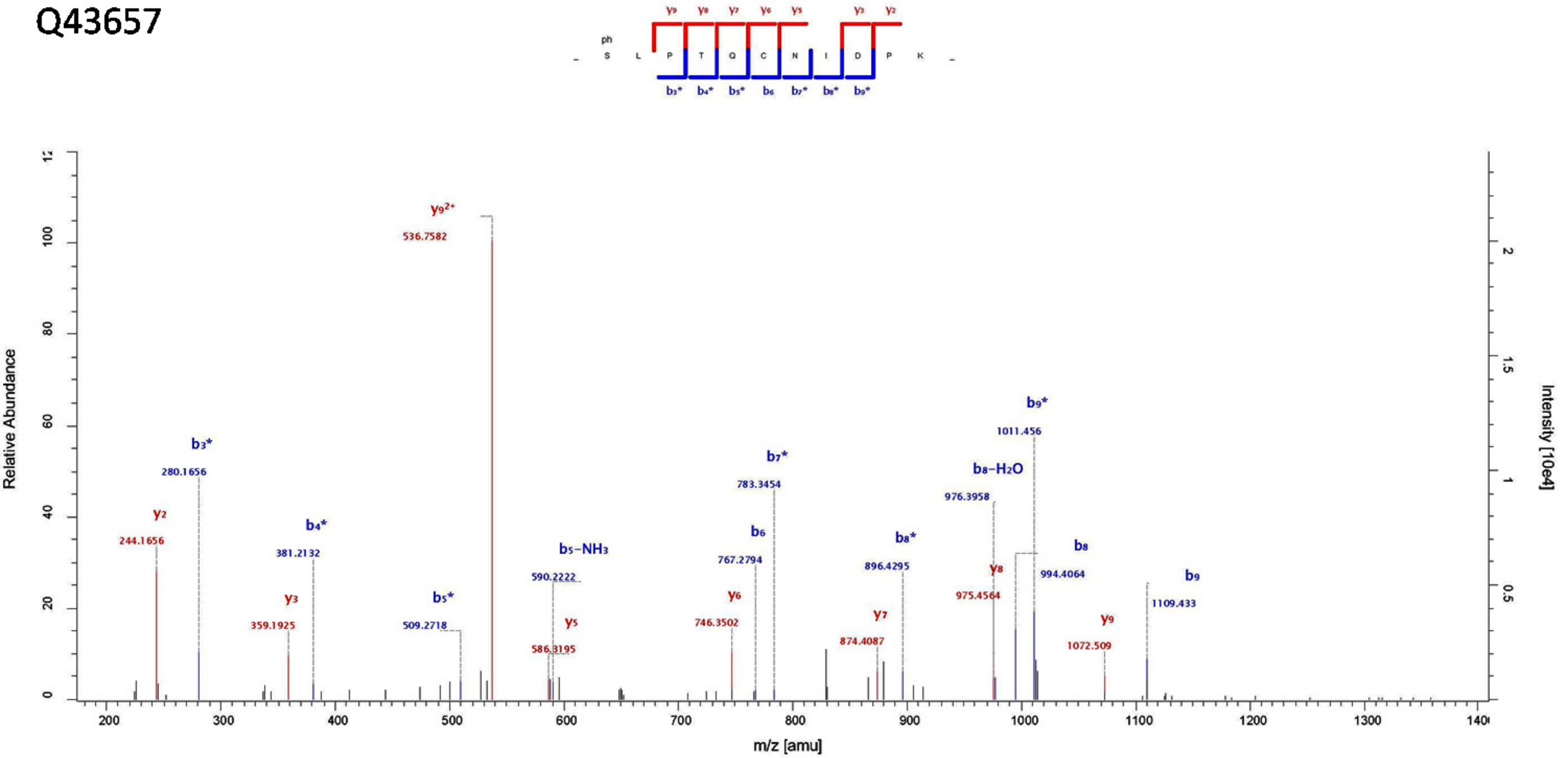

ph
S L P T Q C N I D P K
y9 y8 y7 y6 y5 y3 y2
b3* b4* b5* b6 b7* b8* b9*
Relative Abundance
Intensity [10e4]
m/z [amu]
y9 2+
536.7582
b3*
280.1656
y2
244.1656
b4*
381.2132
y3
359.1925
b5*
509.2718
b5-NH3
590.2222
y5
586.3195
b6
767.2794
b7*
783.3454
y6
746.3502
b8*
896.4295
y7
874.4087
b8-H2O
976.3958
y8
975.4564
b9*
1011.456
b8
994.4064
y9
1072.509
b9
1109.433
